# Taxonomic Revision of the Nearctic Genus *Drepanaphis* Del Guercio (Hemiptera, Aphididae: Drepanosiphinae)

**DOI:** 10.3390/insects15070553

**Published:** 2024-07-21

**Authors:** Kamila Malik, Agnieszka Bugaj-Nawrocka, Karina Wieczorek

**Affiliations:** Institute of Biology, Biotechnology and Environmental Protection, Faculty of Natural Sciences, University of Silesia in Katowice, Bankowa 9, 40-007 Katowice, Poland; kamila.malik@us.edu.pl (K.M.); agnieszka.bugaj-nawrocka@us.edu.pl (A.B.-N.)

**Keywords:** *Acer*, aphids, North America, SEM, sexual morphs

## Abstract

**Simple Summary:**

This study explores the aphid genus *Drepanaphis*, focusing on its diverse species and their relationships with host plants across North America. The research includes a comprehensive taxonomic revision, identifying 18 species with the addition of *Drepanaphis robinsoni* sp. nov. Detailed descriptions and illustrations cover 44 morphs, including alate viviparous females, oviparous females and males, accompanied by new identification keys. For the first time, sexual morphs of 15 species, particularly oviparous females, are documented. Current range maps for all species and microscopy images of key morphological features contribute to a more comprehensive understanding of this genus, which has received limited study in the past.

**Abstract:**

The Nearctic aphid genus *Drepanaphis* Del Guercio, 1909, the largest within the subfamily Drepanosiphinae (Hemiptera: Aphididae), is characterised by distinctive dorsal abdominal tubercles. This study presents a comprehensive taxonomic revision of the genus, expanding the recognised species to 18, including the newly described *Drepanaphis robinsoni* Malik sp. nov. Detailed descriptions and figures for 44 morphs, encompassing alate viviparous females, oviparous females and males, are provided, with new identification keys for all known species and morphs. The sexual morphs of 15 species, particularly oviparous females, are documented for the first time. Morphometric and principal component analyses (PCA) are employed to distinguish the studied taxa. This study identifies and corrects numerous misidentifications in museum collections, previously labelled as *D. acerifoliae*, *D. choanotricha*, *D. kanzensis*, *D. knowltoni*, *D. parva*, *D. sabrinae* or *D. tissoti*. Furthermore, it revalidates the distinct status of *D. nigricans* and *D. tissoti*, which had been synonymised in earlier works. Current range maps for all species and images of key morphological features obtained through light and scanning electron microscopy are also presented, providing a more complete understanding of this understudied genus.

## 1. Introduction

Aphids (Hemiptera: Aphididae) are a diverse group of insects, encompassing around 5600 species divided into 24 subfamilies [1]. They are widespread in temperate regions, with some species exhibiting seasonal alternation between unrelated groups of host plants, including angiosperms, gymnosperms and herbaceous plants [2]. Their life cycles, involving both sexual and asexual reproduction driven by adaptive radiation, contribute to their variable phenotypic features [3]. This great diversity makes aphids a fascinating research model with a complex role in ecosystems. On the one hand, aphids produce honeydew and engage in mutualistic relationships with ants [4]. They also contribute to bioaccumulating chemical elements from plants and contaminated soil [5]. However, they are significant crop and ornamental plant pests, causing damage through direct feeding or the transfer of plant pathogens [6].

The subfamily Drepanosiphinae is a widely distributed group of aphids, currently containing 40 species belonging to five genera. Research on the systematics of Drepanosiphinae has mainly focused on the external morphology and molecular biology of the genera *Drepanosiphoniella* Davatchi, Hille Ris Lambers and Remaudière, 1957 [7]; *Drepanosiphum* Koch, 1855 [8]; *Yamatocallis* Matsumura, 1917 [9]; and *Shenahweum* Hottes & Frison, 1931 [10,11,12,13,14,15]. The genus *Drepanaphis* Del Guercio, 1909 [16], is the most numerous genus within the subfamily, here revised to include 18 species with 44 known morphs. Species from this genus are characterised by distinct dorsal tubercles on the abdominal segments, very long antennae and reduced legs chaetotaxy. All *Drepanaphis* species are regarded as Nearctic, with *D. acerifoliae* (Thomas, 1878) [17] also introduced to Europe [18]. Most species in this genus are similar in the body size and wax arrangement of the dominant morph, alate viviparous females, making them difficult to distinguish. Species of this genus are mostly monophagous, feeding primarily on different maple trees (*Acer* spp.), although some are oligophagous. Exceptionally, the host plant of *D. monelli* (Davis, 1909) [19] is buckeye (*Aesculus glabra*).

Thomas described the first species of the current genus *Drepanaphis* in 1878 as *Siphonophora acerifoliae* [17]. At the end of the description of the new species, he included the following sentence: “It is possible that this Aphis should be placed in *Drepanosiphum*, or a new genus be formed for its reception”. Just a year later, in 1879, Monell classified *S. acerifoliae* in the genus *Drepanosiphum*, changing the species name to *Drepanosiphum acerifolii*. For years, most researchers hesitated to determine whether this species also belonged to the genus *Drepanosiphum*. Finally, on 15 September 1909, Del Guercio [16], in “Revista di Patologia Vegetale”, proposed a new genus for *S. acerifoliae*—*Drepanaphis*. At the same time, in September 1909, Davis proposed the genus *Phymatosiphum* for the same species in the “Annals of the Entomological Society of America”. Davis referred to the suggestions of H. Schouteden and H. F. Wilson that this was not a representative of *Drepanosiphum*. Interestingly, Del Guercio described the new genus from material he received from Davis himself. His footnote at the end of the article proves this: “Gli insetti esaminati ed in base ai quali ho stabilito il genere indicato mi sono stati spediti gentilmente dal chiaro collega J. J. Davis, al quale porgo anche in questa occasione sentiti ringraziamenti.” [transl. “The insects examined and on the basis of which I established the indicated genus were kindly sent to me by my good colleague J. J. Davis, to whom I also offer heartfelt thanks on this occasion.”]. Therefore, if Davis had not shared the material mentioned with Del Guercio, we would probably now be discussing the genus *Phymatosiphum*. Nevertheless, according to the International Code of Zoological Nomenclature (ICZN), Art. 21.3, Del Guercio was the first to describe the genus because its publication date is specified to the day.

Once the systematic position of this genus was established, new species were subsequently described. Davis described *Phymatosiphum monelli* (1909) [19], which Gillette synonymised as *Drepanaphis monelli* (1910) [20]. In 1931, Granovsky [21] described *D. keshenae*; in 1937, Miller [22] proposed *D. sabrinae*. In 1941, Smith [23] described *D. carolinensis*, *D. kanzensis*, *D. nigricans*, *D. parvus*, *D. rubrum* and *D. spicata*. However, in 1943, Smith and Knowlton [24] described two more species—*D. granovskyi* and *D. utahensis*—and at the same time concluded that *D. rubrum* is a synonym of *D. parvus*. Smith described two additional species—*D. tissoti* in 1944 [25] and *D. simpsoni* in 1959 [26]. Then, in 1968, Smith and Dillery [27] carried out the first revision of the genus *Drepanaphis*, describing four more new species—*D. choanotricha*, *D. idahoensis*, *D. knowltoni* and *D. saccharini*. The last species, described by Richards in 1969, was *D. pallida* [28].

As already mentioned, this genus was revised by Smith and Dillery in 1968 [27], and since then, this has been the most reliable source of information about the *Drepanaphis* species. The publication provided important information about the morphology of winged viviparous females, but little attention was given to the descriptions of oviparous females and males. The authors primarily studied interspecific relationships and categorised morphological groups to represent differences between species within a genus. However, they focused extensively on the features of the nymphs without considering the morphological characteristics of the adults. Due to the high similarity of winged forms and the lack of distinct morphological characters between species, the species identity of *D. nigricans* and *D. tissoti* has been questioned, as has that of *D. pallida* and *D. simpsoni* [29].

Therefore, the aim of this study is to perform a comprehensive revision of the genus *Drepanaphis* that considers the variability in species across different regions of North America. This study seeks to document the sexual morphs of 15 species for the first time, including the description of all oviparous females, and to provide comprehensive identification keys for all known species and morphs. A principal components analysis (PCA) is utilised to elucidate relationships among species within the various morphological groups identified in the genus, clarifying the status of the most similar species. Additionally, by examining museum specimens, a new species is described, *Drepanaphis robinsoni* sp. nov., which was previously confused with two other species. It also aims to correct numerous misidentifications of specimens previously mislabelled. Lastly, this study intends to present updated range maps for all species and provide images of key morphological features using light and scanning electron microscopy, thereby contributing to a more complete understanding of this historically understudied genus. 

## 2. Materials and Methods

### 2.1. Study Material and Light Microscopy

A total of 652 microscopic slides and 1382 individuals were examined (1055 alate viviparous females, 61 oviparous females, 42 males and 223 undetermined specimens). Freshly collected samples were preserved in 70% ethanol. Insects were slide mounted using the method of Wieczorek [30]; examined using light microscopes: a Nikon Ni-U, equipped with a phase contrast system and a Leica DM 3000 LED; and photographed using a Leica MC 190 HD camera (Leica Microsystems GmbH, Wetzlar, Germany). The measurements were taken according to Ilharco and van Harten [31] and are given in millimetres. Voucher specimens were deposited in the entomological collection of the University of Silesia in Katowice, Poland (DZUS). Actual host plant names are given according to the WFO Plant List [32]. Final figure processing was performed using Photoscape X 4.2 (photoscape.org, accessed on 20 June 2024). The drawings were prepared manually and then scanned and processed in Photoscape. The dimensions are the average value of several/dozen measurements of individual appendages.

The following abbreviations (in the descriptions, re-descriptions, tables and Supplementary Materials) were used: ABD—abdominal tergite or tergites; ANT—antennae or their lengths; ANT I–VI—antennal segments from I to VI or their lengths (ratios between antennal segments are given as “III/IV”); BASE—basal part of the last antennal segment or its length; BL—body length; DAT—dorsal abdominal tubercles; FEMUR I—fore femur length; FEMUR II—middle femur length; FEMUR III—hind femur length; HW—head width across compound eyes; HT II—second segment of hind tarsus or its length; PT—processus terminalis of the last antennal segment or its length; SIPH—siphunculi sclerite width; TIBIA III—hind tibia length; URS—ultimate segments of rostrum (IV + V) or their lengths.

The material studied was loaned from the following depositories: Biologické centrum IECA—The Biology Centre of the Czech Academy of Sciences; INHS Insect Collection—Illinois Natural History Survey Champaign, Illinois; MNHN—Muséum national d’Histoire naturelle, Paris, France; MZLU—Museum of Zoology, Lund University, Sweden; MZPW—Museum of the Zoological Institute of the Polish Academy of Sciences, Warsaw; NHMUK—Natural History Museum, London, United Kingdom; USNM—United States National Museum, Smithsonian Institution, Washington, DC, United States; ZMUC—Zoological Museum, University of Copenhagen, Copenhagen, Denmark; A. Jensen’s private collection.

The holotype of the new species is deposited at the NHMUK. Paratypes will be deposited at the NHMUK and DZUS.

Quoting the labels of the specimens, a double slash (//) is used to divide data on different labels. Notes and information about the collection are in square brackets [ ].

### 2.2. Scanning Electron Microscopy

Specimens for scanning electron microscopy (SEM) analysis (five individuals) were preserved in 70% ethanol. The samples were dehydrated using serial baths of 80%, 90% and 96% ethanol—20 min for 80% ethanol, 15 min for 90% ethanol, 10 min for 96% ethanol and two baths of absolute alcohol for 10 min each. Dehydrated samples were dried using a Leica EM CPD300 automated critical point dryer (Leica Microsystems, Vienna, Austria). Dry samples were mounted on aluminium stubs with double-sided adhesive carbon tape and sputter coated with a 30 nm gold layer in a Safematic CCU-010 high-vacuum sputter coater (Safematic GmbH, Zizers, Switzerland). The specimens were imaged with Hitachi SU8010 (Hitachi High-Technologies Corporation, Tokyo, Japan) and Phenom XL (Phenom-World B.V., Eindhoven, The Netherlands) field emission scanning electron microscopes. Final figure processing was performed using Photoscape X 4.2 (photoscape.org, accessed on 20 June 2024).

### 2.3. Statistical Analysis

A principal components analysis (PCA) was conducted based on the data recorded from individual specimens. Data sets (Tables S1 and S2) with morphometric variables, morphometric ratios and morphological characters for alate viviparous females (52 characters), males (51 characters) and oviparous females (46 characters) were tested with multiple correlation analysis, and the variables with the lowest redundancy values were finally selected. Six morphometric variables, ten morphometric ratios and eight morphological characters were selected for the 213 alate viviparous females of the genus *Drepanaphis* (see Table S3 for details). For the 30 males (see Table S4 for more information), four morphometric variables, 11/12 morphometric ratios and nine morphological characters were selected. In turn, for the 43 oviparous females (see Table S4 for more information), five morphometric variables, 13 morphometric ratios and six morphological characters were selected (all variables are listed in Tables S1 and S2). Before the PCA, each character was converted to zero mean and unit standard deviation within reduced data sets, so the same weight was given to all of them. The PAST software ver. 4.13 [33] was used for multivariate analyses. Since we have already analysed distinctiveness at the generic level with representatives of *Drepanaphis* and the closely related genera *Drepanosiphum* and *Drepanosiphoniella*, we will not repeat it here and will refer to the previous publication [12].

### 2.4. Occurrence Data and Preparation of Maps

The occurrence data were obtained from the scientific literature, specimens studied in museum collections, fieldwork in the USA in September 2022, iNaturalist (www.inaturalist.org, accessed on 12 June 2024) [34] and biodiversity databases (GBIF Occurrence Download [35], https://doi.org/10.15468/dl.nsv6vq, accessed on 12 June 2024). Museum curators were asked to provide information about their collections (photographs of the preparations were provided). Some specimens were also examined in the collections during the personal stay of the first author. Databases were searched based on keywords, i.e., the name of the species and its synonyms. All records with unspecified or unknown localities were excluded.

All localities of the studied species were georeferenced using Google Earth ver. 10.38.0.0 (Google Inc. [36], Mountain View, CA, USA) (geographical projection, decimal degrees, datum: WGS84). The ranges of host plants were based on data obtained from http://databasin.org, accessed on 12 June 2024 (Conservation Biology Institute (CBI) [37]; the maps are a digital representation of the tree species range maps from the Atlas of the United States Trees by Little [38]). Maps were prepared using Quantum GIS ver. 3.30.1 (QGIS Development Team [39]) using the WGS84 datum and EPSG: 4326 or 3857 (Web Mercator). The data on the distribution of individual species will be published in the GBIF.

## 3. Results

### 3.1. Taxonomy

**Figure 1:**
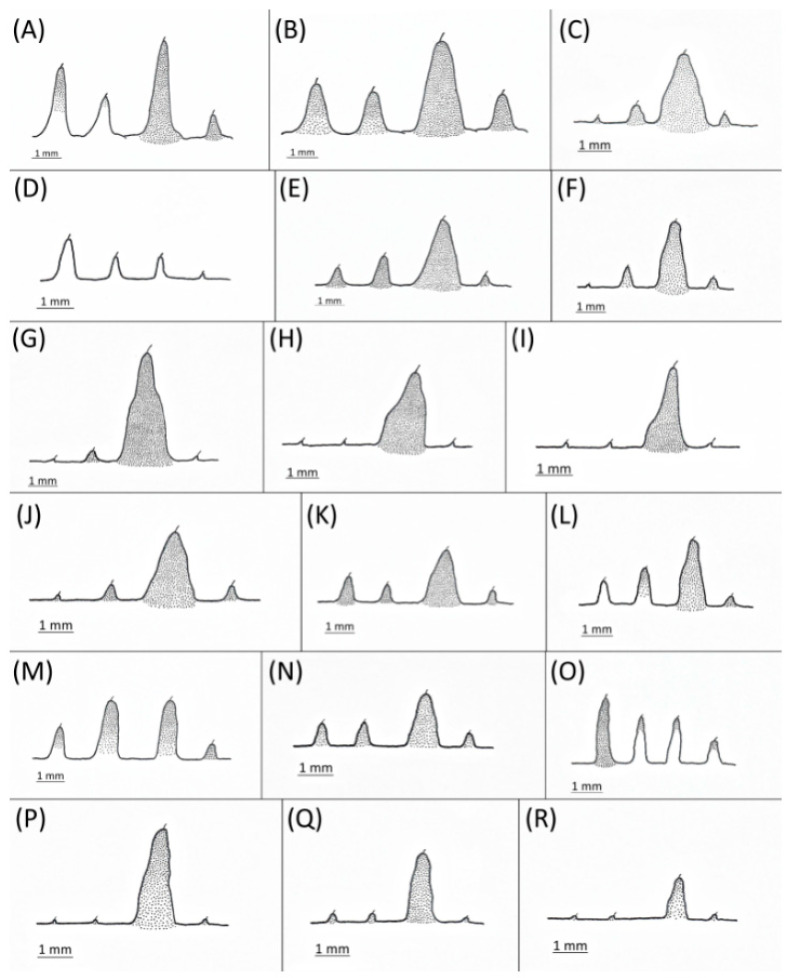
Lateral arrangement of dorsal abdominal tubercles of alate viviparous females of the genus *Drepanaphis*: (**A**) *D. acerifoliae*, (**B**) *D. carolinensis*, (**C**) *D. choanotricha*, (**D**) *D. granovskyi*, (**E**) *D. idahoensis*, (**F**) *D. kanzensis*, (**G**) *D. keshenae*, (**H**) *D. knowltoni*, (**I**) *D. monelli*, (**J**) *D. nigricans*, (**K**) *D. parva*, (**L**) *D. robinsoni* sp. nov., (**M**) *D. sabrinae*, (**N**) *D. saccharini*, (**O**) *D. simpsoni*, (**P**) *D. spicata*, (**Q**) *D. tissoti*, (**R**) *D. utahensis*.

**Figure 2:**
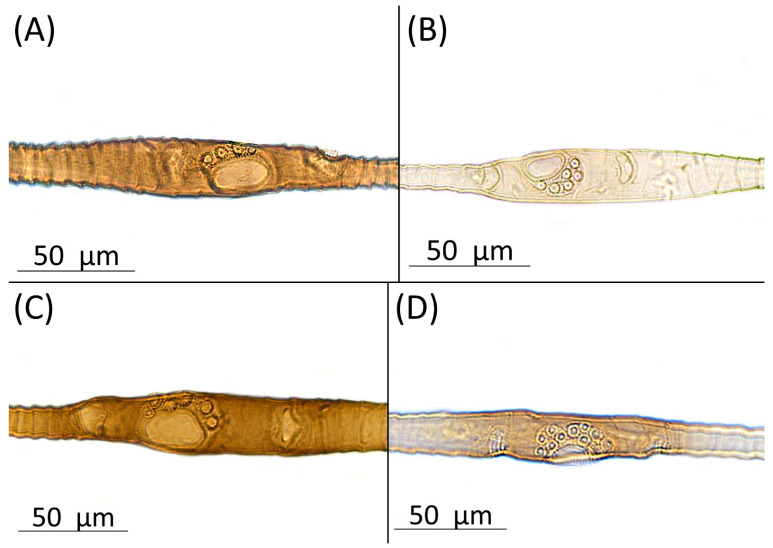
Antennal segment VI base with primary rhinarium (bigger) and accessory rhinaria (smaller) of alate viviparous females of the genus *Drepanaphis*: (**A**) *D. acerifoliae*, (**B**) *D. choanotricha*, (**C**) *D. sabrinae*, (**D**) *D. tissoti*.

**Figure 3:**
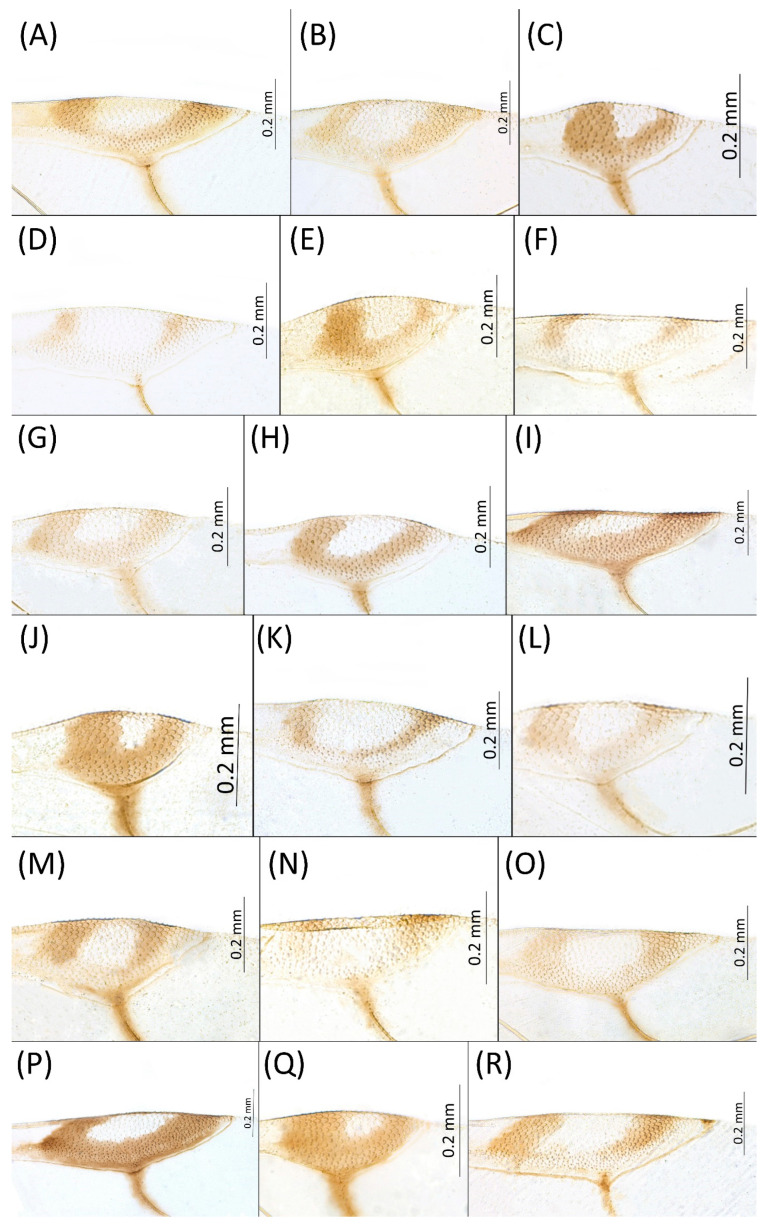
Pterostigma of the fore wing of alate viviparous females of the genus *Drepanaphis*: (**A**) *D. acerifoliae*, (**B**) *D. carolinensis*, (**C**) *D. choanotricha*, (**D**) *D. granovskyi*, (**E**) *D. idahoensis*, (**F**) *D. kanzensis*, (**G**) *D. keshenae*, (**H**) *D. knowltoni*, (**I**) *D. monelli*, (**J**) *D. nigricans*, (**K**) *D. parva*, (**L**) *D. robinsoni* sp. nov., (**M**) *D. sabrinae*, (**N**) *D. saccharini*, (**O**) *D. simpsoni*, (**P**) *D. spicata*, (**Q**) *D. tissoti*, (**R**) *D. utahensis*.

**Figure 4:**
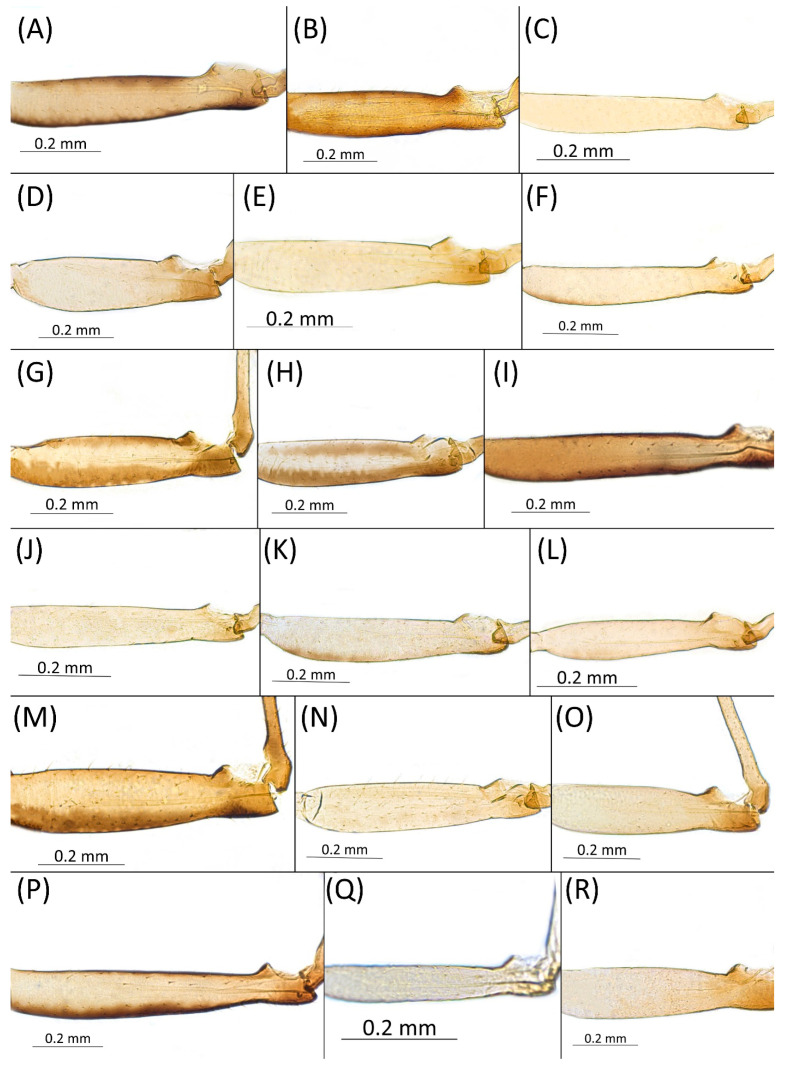
Fore femora of alate viviparous females of the genus *Drepanaphis*: (**A**) *D. acerifoliae*, (**B**) *D. carolinensis*, (**C**) *D. choanotricha*, (**D**) *D. granovskyi*, (**E**) *D. idahoensis*, (**F**) *D. kanzensis*, (**G**) *D. keshenae*, (**H**) *D. knowltoni*, (**I**) *D. monelli*, (**J**) *D. nigricans*, (**K**) *D. parva*, (**L**) *D. robinsoni* sp. nov., (**M**) *D. sabrinae*, (**N**) *D. saccharini*, (**O**) *D. simpsoni*, (**P**) *D. spicata*, (**Q**) *D. tissoti*, (**R**) *D. utahensis*.

**Figure 5:**
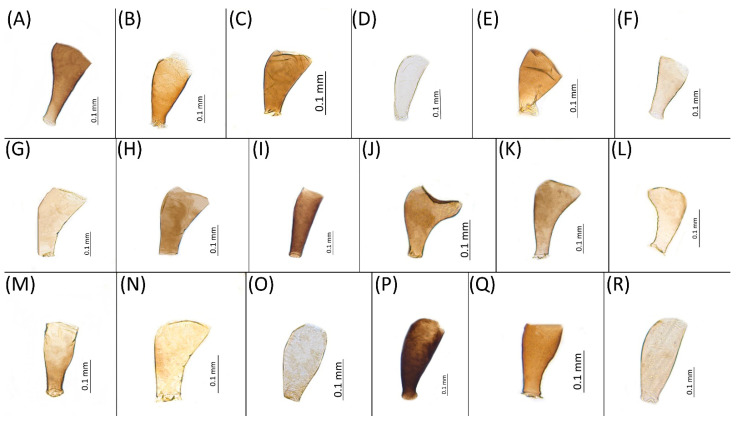
Siphunculi of alate viviparous females of the genus *Drepanaphis*: (**A**) flask-shaped in *D. acerifoliae*, (**B**) flask-shaped in *D. carolinensis*, (**C**) flask-shaped in *D. choanotricha*, (**D**) tubular in *D. granovskyi*, (**E**) flask-shaped in *D. idahoensis*, (**F**) flask-shaped in *D. kanzensis*, (**G**) flask-shaped in *D. keshenae*, (**H**) flask-shaped in *D. knowltoni*, (**I**) tubular in *D. monelli*, (**J**) flask-shaped in *D. nigricans*, (**K**) flask-shaped in *D. parva*, (**L**) flask-shaped in *D. robinsoni* sp. nov., (**M**) tubular in *D. sabrinae*, (**N**) flask-shaped in *D. saccharini*, (**O**) tubular in *D. simpsoni*, (**P**) flask-shaped in *D. spicata*, (**Q**) flask-shaped in *D. tissoti*, (**R**) tubular in *D. utahensis*.

**Genus *Drepanaphis* Del Guercio, 1909**
Type species *Siphonophora acerifoliae* Thomas, 1878, by original designation.
**Diagnosis:** Dominant morph is alate viviparous female, characterised by distinct dorsal abdominal tubercles, variably developed on ABD I–IV (Figure 1). Oviparous females apterous, males alate. All morphs with rounded secondary rhinaria on ANT III. Primary and accessory rhinaria on BASE ciliated (Figure 2). Pterostigma distinct, darkly pigmented, with small area inside without pigmentation or palely pigmented, large area inside without pigmentation (Figure 3). Fore femora pale, dark or darker dorsally (Figure 4). Siphunculi tubular or flask-shaped (Figure 5), placed on ABD VI, swollen at base, without subapical reticulation. Almost all species are associated with species of *Acer*, except for *D. monelli*, which is found on species of *Aesculus*. They usually do not form dense colonies and are not attended by ants.

**Shared morphological characters of alate viviparous females of the genus *Drepanaphis***

**Figure 6:**
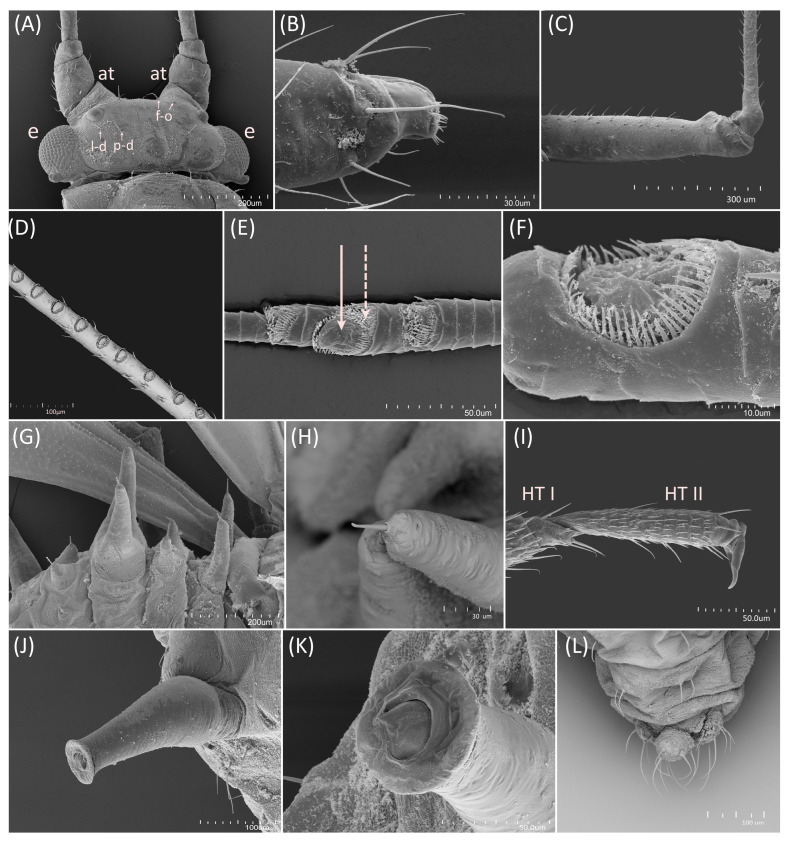
Scanning electron microscopy (SEM) of the most important generic features of the genus *Drepanaphis* on the basis of the type species *D. acerifoliae*: (**A**) dorsal view of head showing compound eyes (e), little-developed antennal tubercles (at), fronto-orbital setae (f-o), postero-dorsal head setae (p-d) and latero-dorsal head setae (l-d); (**B**) ultimate rostral segments with trichoid sensilla (solid arrow), apex of rostrum with basiconic sensilla (dotted arrow); (**C**) ultrastructure of fore femora; (**D**) ANT III with secondary rounded and ciliated rhinaria; (**E**) ANT VI sensilla, big multiporous placoid sensillum: primary rhinarium (solid arrow) and sunken coeloconic sensilla: accessory rhinaria (dotted arrow); (**F**) apical part of ANT V with big multiporous placoid sensillum; (**G**) arrangement of dorsal tubercles on abdomen; (**H**) basiconic sensilla at the end of tubercles; (**I**) hind tarsus with short first segment (HT I) and longer second segment (HT II) with claws; (**J**) lateral side of end of abdomen with siphunculus (SIPH); (**K**) apical part of siphunculus with wide flange; (**L**) dorsal side of end of abdomen with knobbed cauda.

Body slender, pale, with various pigmentation patterns on legs. Head separated from pronotum, abdominal segments not separated from one another. Head with little-developed antennal tubercles, with postero-dorsal, latero-dorsal and fronto-orbital setae (Figure 6A). Compound eyes with numerous ommatidia and well-developed triommatidia (Figure 6A). Rostrum ends between fore and middle coxae. Ultimate rostral segments with two pairs of primary setae and four to seven pairs of accessory setae, variable within species (Figure 6B). Antennae six-segmented. Antennal segment II shortest; antennal segments IV and V similar in length; processus terminalis longest. Antennae covered with pointed, short, colourless setae. On ANT I–II 0.01–0.02 mm long (on ANT I more abundant on apical part of segment); on ANT III–VI 0.005–0.01 mm long. ANT I with 8–12 setae, ANT II with 2–5 setae, ANT III with 40–50 setae, ANT IV with 12–18 setae, ANT V with 7–9 setae, BASE with 2–3 setae (type I trichoid sensilla), PT with 2 subapical and 1 apical setae (type II trichoid sensilla). ANT III with 2–22 rounded secondary rhinaria (small multiporous placoid sensilla; Figure 6D). Apical part of antennal segment V with one primary rhinarium (big multiporous placoid sensillum; Figure 6F). Base of antennal segment VI with 1 primary (major) rhinarium (big multiporous placoid sensillum) and 4–11 accessory rhinaria (small multiporous placoid sensilla) adhering to primary rhinarium, number of which may vary between species. Above and below major rhinarium, additional primary rhinaria present (Figure 6E). All rhinaria with ciliated cuticle edges (Figure 6D–F). Fore wings with radius strongly curved, media twice branched. Hind wings with media present. Dorsal abdominal tubercles (Figure 6G) with trichoid sensilla type I at ends (Figure 6H). Dorsal setae pointed, blunt or forked. Siphunculi flask-shaped or tubular (Figure 6J). Apex of siphunculi with well-developed, strong flange and well-developed operculum on siphuncular pore (Figure 6K). Fore femora dark dorsally or pale, hind femora pale, smudged or with dark stripes on distal parts. Legs covered by not numerous, 0.01–0.05 mm long, fine setae with pointed apices. Femora with smaller amount of setae, and tibiae with more abundant setae, especially at end (Figure 6C). First tarsal segments 4:4:4, empodial setae spatulate (Figure 6I). Cauda knobbed, with 4–6 long, fine and pointed setae (Figure 6L). Anal plate and genital plate covered by numerous fine and pointed setae.

**Shared morphological characters of oviparous females of the genus *Drepanaphis***

Body pear-shaped or oval with elongated end of abdomen. Dorsal abdominal tubercles absent. ANT III without or with single, small, rounded secondary rhinaria. BASE with 1 rounded primary rhinarium with ciliated edge and 6–7 very small accessory rhinaria, adhering to primary rhinarium. Dorsal setae arranged in marginal, pleural and spinal rows. ABD I–VI with blunt setae distributed on well-developed sclerites. ABD VII–VIII with pointed setae. Hind tibiae with rounded pseudosensoria, mostly arranged along almost their entire lengths. Cauda knobbed with numerous setae.

**Shared morphological characters of alate males of the genus *Drepanaphis***

General characters like in alate viviparous females. ANT III–V with numerous, small, rounded secondary rhinaria. BASE with 1 rounded primary rhinarium with ciliated edge and 4–5 very small accessory rhinaria, adhering to primary rhinarium. Abdomen with well-developed dorsal sclerotisation, especially on ABD IV–V. Dorsal abdominal tubercles smaller and less visible than in alate viviparous females. In some species inconspicuous. Cauda more or less knobbed, with five long, fine, pointed setae. Genitalia dusky, except for *D. granovskyi*, with genitalia distinctly darkly pigmented. In all known males parameres large, lobate, except *D. simpsoni*, with parameres much smaller and elongated. Parameres and basal part of phallus covered with numerous short setae. Distal part of sclerotised arms rather long and thin, whereas proximal part shorter and wider.

### 3.2. Keys to Species of the Genus Drepanaphis

#### 3.2.1. Key to the Identification of Alate Viviparous Females of the Genus *Drepanaphis*

**Figure 7:**
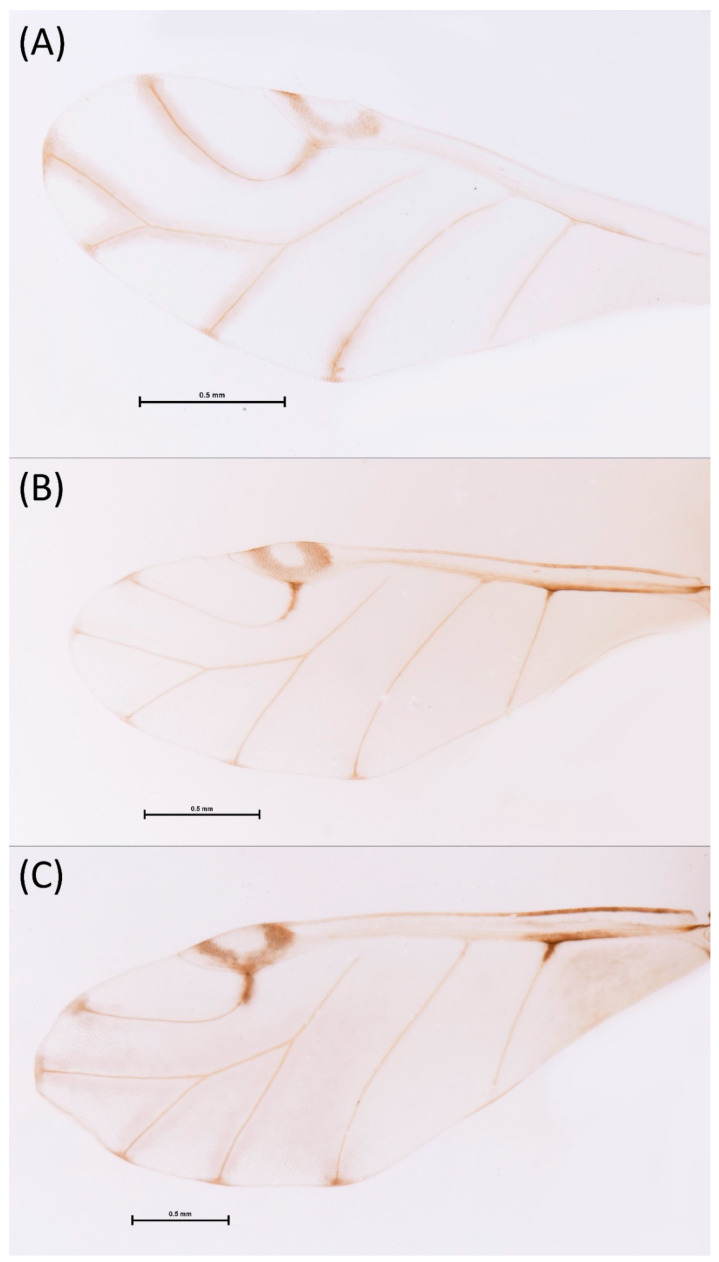
Wing pigmentation: (**A**) wing veins distinctly bordered in *D. acerifoliae*, (**B**) wing veins clear in *D. carolinensis*, (**C**) wing veins diffusely bordered in *D. parva*.

1. Femur I pigmented for its full length, especially dorsally (Figure 4A,B,G–I,M,P)……………………………………………………………………………2

- Femur I pale, slightly pigmented basally (Figure 4C–F,J–L,N,O,Q,R)………………………………………………………………………………8

2. Wing veins distinctly bordered (Figure 7A)………………………………………………………………………………3

- Wing veins clear (Figure 7B)…………………………………………………………………………………………………4

3. Conspicuous four pairs of dorsal abdominal tubercles. ABD I pale at base and darker at tips, ABD II pale, ABD III–IV dark brown; third pairs of tubercles biggest (Figure 1A)…………………………………………………………………………………………………………*D. acerifoliae*

- Conspicuous one pair of dark brown dorsal abdominal tubercles on tergite III (Figure 1G)……………………………………………………………………………………*D. keshenae*

4. Conspicuous four pairs of dorsal abdominal tubercles……………………………………5

- Conspicuous one pair of dorsal abdominal tubercles on tergite III………………………6

5. First and second pair of dorsal abdominal tubercles equal, third pair biggest, fourth pair smallest (Figure 1B); BASE with 4 accessory rhinaria; ANT III with 9–15 secondary rhinaria…………………………………………………………………………………………*D. carolinensis*

- Second and third pairs of dorsal abdominal tubercles equal, first and fourth pairs smallest (Figure 1M); BASE with 5–6 accessory rhinaria (Figure 2C); ANT III with 6–10 secondary rhinaria……………………………………………………………*D. sabrinae*

6. Fore femora > 0.8 mm long, frontal setae 0.09–0.12 mm long……………………*D. spicata*

- Fore femora < 0.8 mm long, frontal setae > 0.09 mm long………………………………7

7. Fore femora dark (Figure 4I), BASE always with four accessory rhinaria, on *Aesculus glabra*…………………………………………………………………………………*D. monelli*

- Fore femora darker dorsally (Figure 4H), BASE with four or five accessory rhinaria, on *Acer grandidentatum*…………………………………………………………………*D. knowltoni*

8. Wing veins diffusely bordered (Figure 7C)………………………………………………9

- Wing veins clear (Figure 7B)………………………………………………………………10

9. BL > 1.85 mm long; body with brown dorsal sclerotisation; SIPH shaded or dark (Figure 5K)………………………………………………………………………………*D. parva*

- BL < 1.85 mm long; body without brown dorsal sclerotisation; SIPH pale (Figure 5L)……………………………………………………………………………*D. robinsoni* sp. nov.

10. Two pairs of frontal setae…………………………………………………………………11

- One pair of frontal setae……………………………………………………………………13

11. Conspicuous one pair of dorsal abdominal tubercles on tergite III (Figure 1R)……………………………………………………………………………………*D. utahensis*

- Conspicuous more than one pair of dorsal abdominal tubercles……………………12

12. Entire body pale; DAT I–III short, first pair larger than second and third (Figure 1D)………………………………………………………………………………………*D. granovskyi*

- DAT I biggest, darker than others (Figure 1O)………………………………………*D. simpsoni*

13. Pterostigma distinct, darkly pigmented, with small area inside without pigmentation; SIPH completely dark; dorsal setae blunt………………………………………………………14

- Pterostigma palely pigmented with large area inside without pigmentation, SIPH shaded or pale, dorsal setae pointed……………………………………………………17

14. BASE with more than four accessory rhinaria…………………………………………15

- BASE with four accessory rhinaria………………………………………………………16

15. ANT III with 2–5 secondary rhinaria, frontal setae 0.06–0.08 mm long, dorsal setae 0.03–0.05 mm long…………………………………………………………*D. choanotricha*

- ANT III with 8–14 secondary rhinaria, frontal setae 0.05–0.06 mm long, dorsal setae 0.01–0.02 mm long……………………………………………………………………*D. tissoti*

16. ANT III with 6–9 secondary rhinaria, dorsal setae 0.02–0.04 mm long……*D. idahoensis*

- ANT III with 12–18 secondary rhinaria, dorsal setae 0.01–0.02 mm long……*D. nigricans*

17. Conspicuous four pairs of dorsal abdominal tubercles (Figure 1N)………*D. saccharini*

- Conspicuous three pairs of dorsal abdominal tubercles, on ABD III biggest, on DAT II and IV very small (Figure 1F)…………………………………………………*D. kanzensis*

#### 3.2.2. Key to the Identification of Known Oviparous Females of the Genus *Drepanaphis*

1. BASE with more than four accessory rhinaria………………………………………………2

- BASE with four accessory rhinaria…………………………………………………………4

2. ANT segment ratio PT/BASE < 12; >70 pseudosensoria on hind tibiae; legs and antennae very dark, siphunculi tubular………………………………………………*D. sabrinae*

- ANT segment ratio PT/BASE > 12, siphunculi flask-shaped……………………………3

3. ANT III > 0.9 mm long; > 30 pseudosensoria; SIPH/BL 0.07–0.09…………………*D. tissoti*

- ANT III < 0.9 mm long; < 30 pseudosensoria; SIPH/BL 0.12………………*D. choanotricha*

4. Two pairs of frontal setae………………………………………………………………………5

- One pair of frontal setae…………………………………………………………………………7

5. ANT III < 0.05 mm long; dorsal setae evidently forked………………………*D. granovskyi*

- ANT III > 0.05 mm long…………………………………………………………………………6

6. URS/ANT III < 0.13 mm long…………………………………………………………*D. utahensis*

- URS/ANT III > 0.13 mm long…………………………………………………………*D. simpsoni*

7. Legs and SIPH dark……………………………………………………………………………8

- Legs and SIPH pale……………………………………………………………………………11

8. SIPH/BL > 0.09; HT II/ANT VI < 0.1…………………………………………………*D. monelli*

- SIPH/BL < 0.09; HT II/ANT VI > 0.1……………………………………………………………9

9. URS/BASE < 0.07……………………………………………………………………*D. carolinensis*

- URS/BASE > 0.07………………………………………………………………………………10

10. ANT > 2.6 mm long; SIPH > 0.2 mm long…………………………………………*D. acerifoliae*

- ANT < 2.6 mm long; SIPH < 0.2 mm long…………………………………………………*D. keshenae*

11. ANT segment ratio PT/BASE > 12………………………………………………*D. nigricans*

- ANT segment ratio PT/BASE < 12…………………………………………………………12

12. SIPH/BL < 0.08; URS < 0.09………………………………………………………*D. kanzensis*

- SIPH/BL > 0.08; URS > 0.09…………………………………………………………………13

13. ANT segment ratio VI/III < 1; 22–23 pseudosensoria………………………*D. idahoensis*

- ANT segment ratio VI/III > 1; 59–71 pseudosensoria……………………………*D. spicata*

#### 3.2.3. Key to the Identification of Known Alate Males of the Genus *Drepanaphis*

1. Femur I pigmented for its entire length, two pairs of frontal setae, dorsal abdominal tubercles inconspicuous………………………………………………………*D. granovskyi*

- Femur I pigmented dorsally…………………………………………………………………2
- Femur I pale, slightly pigmented basally…………………………………………………7

2. Wing veins distinctly bordered, conspicuous one pair of dorsal abdominal tubercles on tergite III……………………………………………………………………………………3

- Wing veins clear……………………………………………………………………………4

3. Hind tibiae with dark area in apical part; PT/BASE < 0.09……………………*D. acerifoliae*

- Hind tibiae pale; PT/BASE > 0.09…………………………………………………*D. keshenae*

4. Dorsal abdominal tubercles inconspicuous, BL 2.8 mm long…………………*D. spicata*

- Conspicuous one pair of dorsal abdominal tubercles on tergite III……………………5

5. URS/ANT III > 0.12………………………………………………………………*D. monelli*

- URS/ANT III < 0.12…………………………………………………………………………6

6. ANT III with > 80 rhinaria, hind tibiae < 1.2 mm long; SIPH < 0.25………*D. carolinensis*

- ANT III with < 80 rhinaria, hind tibiae > 1.2 mm long; SIPH > 0.25…………*D. knowltoni*

7. Conspicuous more than one pair of dorsal abdominal tubercles, wing veins diffusely bordered, body with brown dorsal sclerotisation………………………………*D. parva*

- Wing veins clear…………………………………………………………………………………8

8. Dorsal abdominal tubercles inconspicuous, two pairs of frontal setae…………………9

- Conspicuous one pair of dorsal abdominal tubercles on tergite III………………………10

9. ANT segment ratio PT/BASE < 6; SIPH/BL < 0.09………………………………*D. simpsoni*

- ANT segment ratio PT/BASE > 6; SIPH/BL > 0.09………………………………*D. utahensis*

10. ANT segment ratio PT/BASE > 6; SIPH pale……………………………………*D. kanzensis*

- ANT segment ratio PT/BASE < 6; SIPH dark.………………………………………*D. choanotricha*

### 3.3. Checklist of Species in the Genus Drepanaphis

Family: Aphididae Latreille, 1802
  Subfamily: Drepanosiphinae Herrich-Schaeffer, 1857
  Genus: *Drepanaphis* Del Guercio, 1909
  *Drepanaphis* Del Guercio, 1909: 4: 49
  = *Phymatosiphum* Davis, 1909: 2: 196
  = *Drepanphis* Takahashi, 1923: 4: 66 (subsequent misspelling)
  1. *Drepanaphis acerifoliae* (Thomas, 1878)
  2. *Drepanaphis carolinensis Smith*, 1941
  3. *Drepanaphis choanotricha* Smith & Dillery, 1968
  4. *Drepanaphis granovskyi* Smith & Knowlton, 1943
  5. *Drepanaphis idahoensis* Smith & Dillery, 1968
  6. *Drepanaphis kanzensis* Smith, 1941
  7. *Drepanaphis keshenae* Granovsky, 1931
  8. *Drepanaphis knowltoni* Smith & Dillery, 1968
  9. *Drepanaphis monelli* (Davis, 1909)
  10. *Drepanaphis nigricans* Smith, 1941
  11. *Drepanaphis parva* Smith, 1941
  12. *Drepanaphis robinsoni* Malik sp. nov.
  13. *Drepanaphis sabrinae* Miller, 1937
  14. *Drepanaphis saccharini* Smith & Dillery, 1968
  15. *Drepanaphis simpsoni* Smith, 1959
  16. *Drepanaphis spicata* Smith, 1941
  17. *Drepanaphis tissoti* Smith, 1941 stat. rev.
  18. *Drepanaphis utahensis* Smith & Knowlton, 1943

### 3.4. Review of Species

#### 3.4.1. *Drepanaphis acerifoliae* (Thomas, 1878)

**Figure 8:**
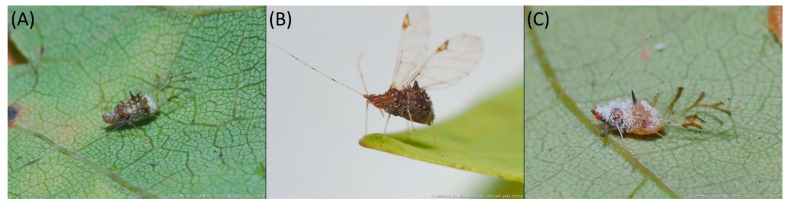
Live specimens of the genus Drepanaphis: (**A**) *D. acerifoliae*, (**B**) *D. carolinensis*, (**C**) *D. keshenae*. Image copyright V. Charny, under a Creative Commons 3.0 License.

**Figure 9:**
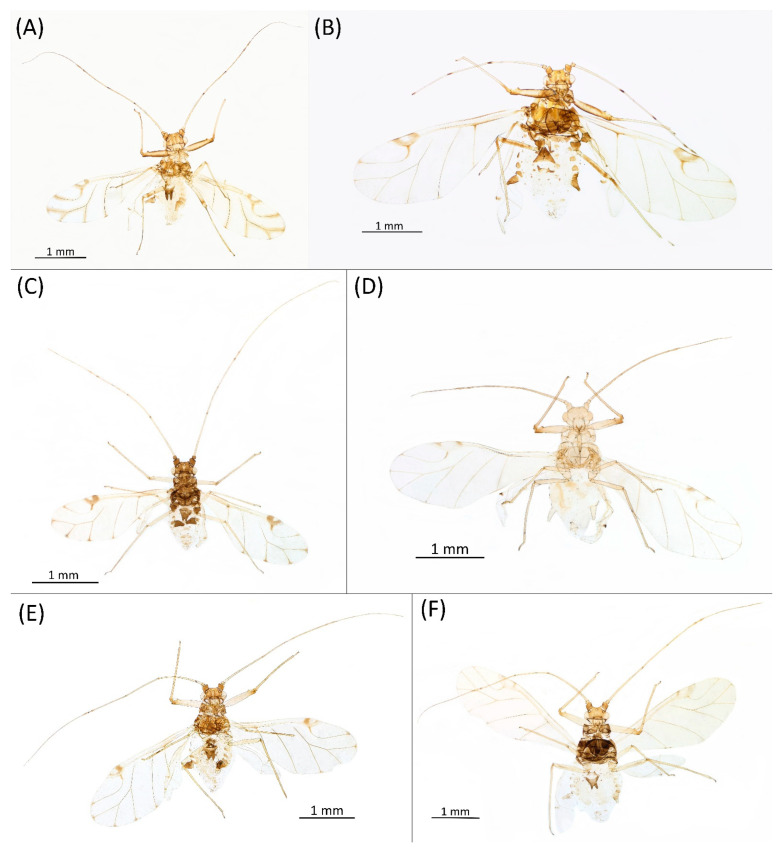
Alate viviparous females of the genus Drepanaphis: (**A**) *D. acerifoliae*, (**B**) *D. carolinensis*, (**C**) *D. choanotricha*, (**D**) *D. granovskyi*, (**E**) *D. idahoensis*, (**F**) *D. kanzensis*.

**Figure 10:**
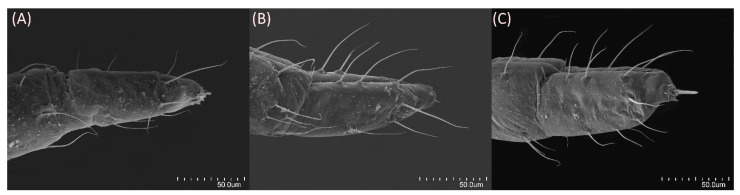
Ultimate rostral segments with trichoid sensilla of selected species of the genus Drepanaphis: (**A**) *D. acerifoliae*, (**B**) *D. monelli*, (**C**) *D. utahensis*.

**Figure 11:**
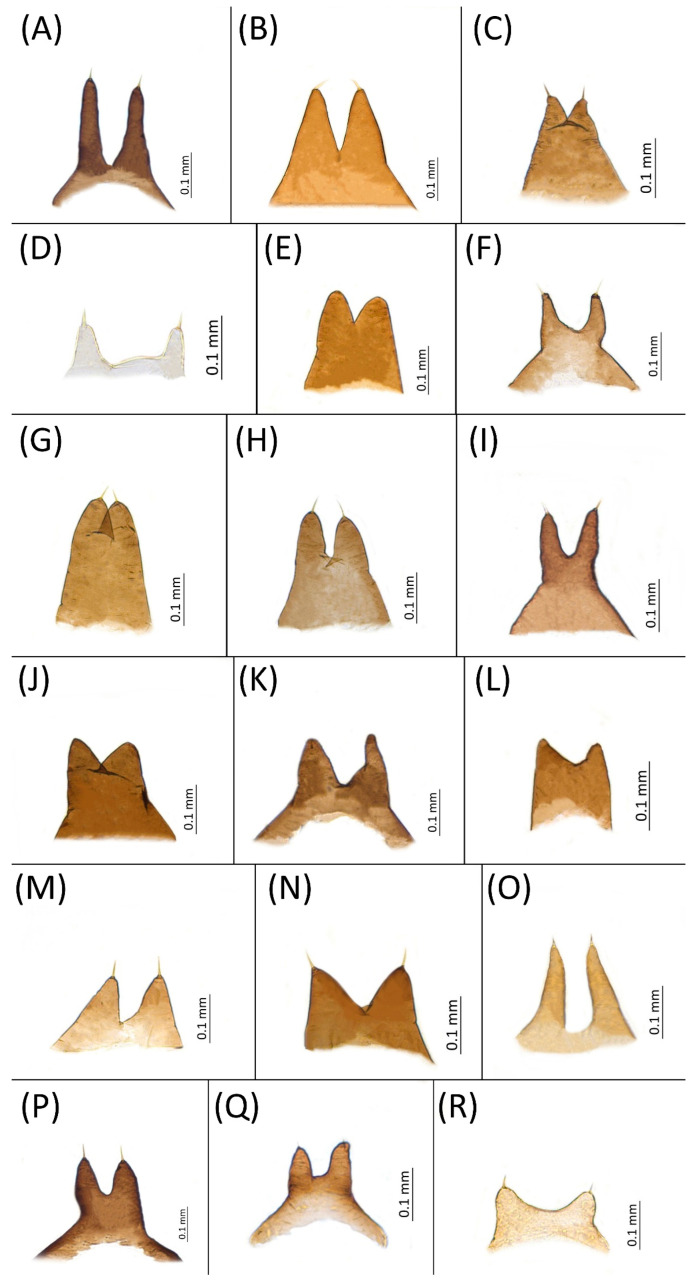
Shape of the third pair of dorsal abdominal tubercles of alate viviparous females of the genus Drepanaphis: (**A**) *D. acerifoliae*, (**B**) *D. carolinensis*, (**C**) *D. choanotricha*, (**D**) *D. granovskyi*, (**E**) *D. idahoensis*, (**F**) *D. kanzensis*, (**G**) *D. keshenae*, (**H**) *D. knowltoni*, (**I**) *D. monelli*, (**J**) *D. nigricans*, (**K**) *D. parva*, (**L**) *D. robinsoni sp. nov.*, (**M**) *D. sabrinae*, (**N**) *D. saccharini*, (**O**) *D. simpsoni*, (**P**) *D. spicata*, (**Q**) *D. tissoti*, (**R**) *D. utahensis*.

**Figure 12:**
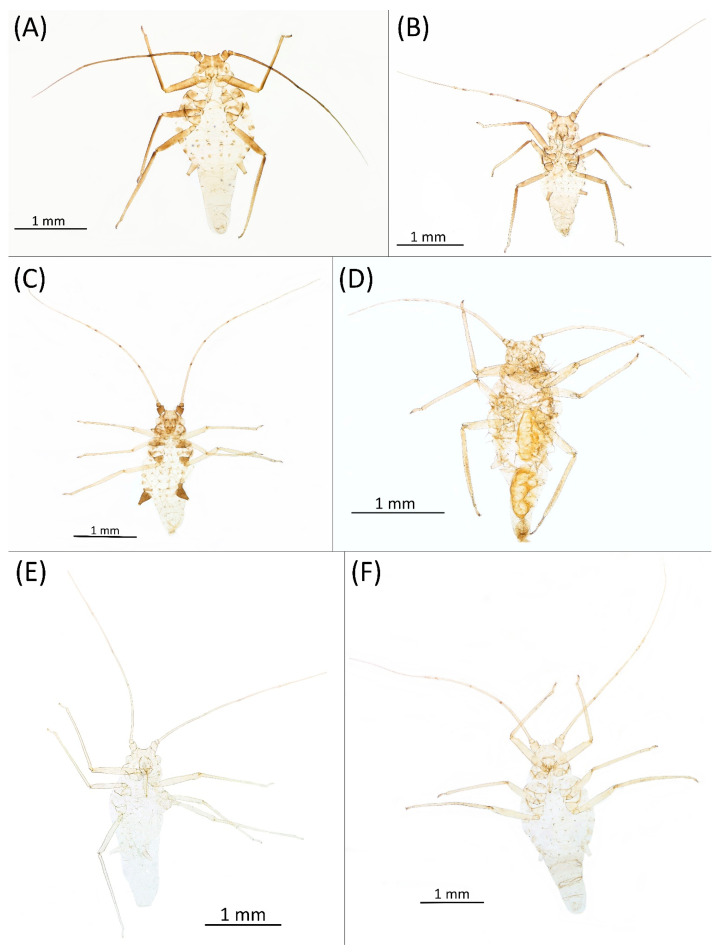
Oviparous females of the genus Drepanaphis: (**A**) *D. acerifoliae*, (**B**) *D. carolinensis*, (**C**) *D. choanotricha*, (**D**) *D. granovskyi*, (**E**) *D. idahoensis*, (**F**) *D. kanzensis*.

**Figure 13:**
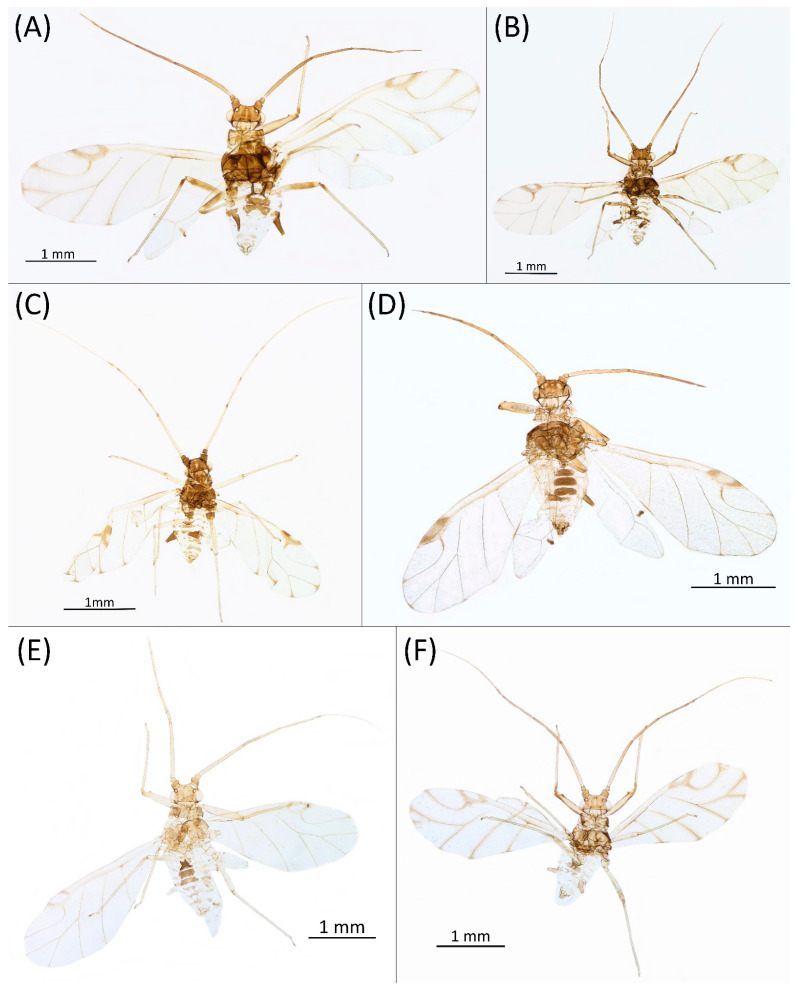
Alate males of the genus Drepanaphis: (**A**) *D. acerifoliae*, (**B**) *D. carolinensis*, (**C**) *D. choanotricha*, (**D**) *D. granovskyi*, (**E**) *D. kanzensis*, (**F**) *D. keshenae*.

**Figure 14:**
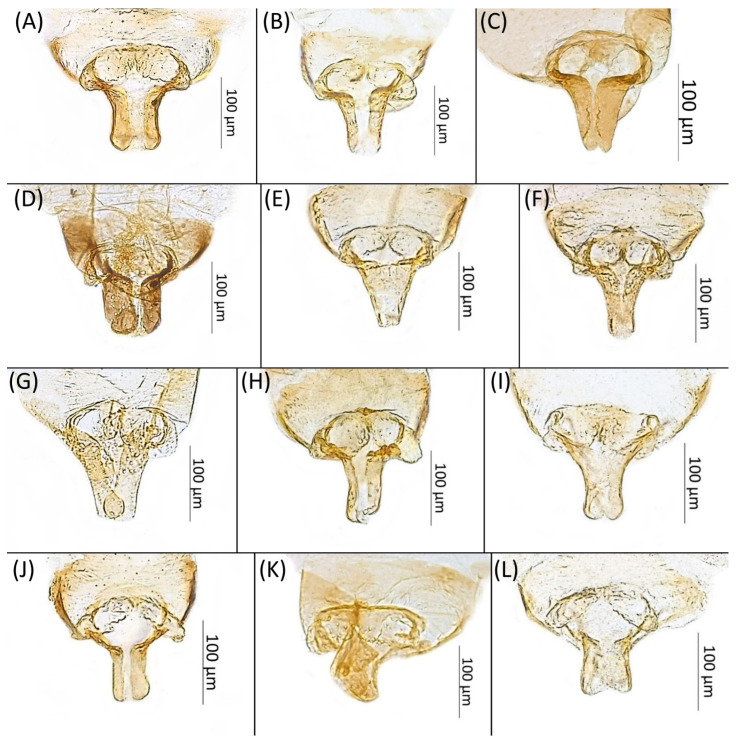
Genitalia of the known males of the genus Drepanaphis: (**A**) *D. acerifoliae*, (**B**) *D. carolinensis*, (**C**) *D. choanotricha*, (**D**) *D. granovskyi*, (**E**) *D. kanzensis*, (**F**) *D. keshenae*, (**G**) *D. knowltoni*, (**H**) *D. monelli*, (**I**) *D. parva*, (**J**) *D. simpsoni*, (**K**) *D. spicata*, (**L**) *D. utahensis*.

**Figure 15:**
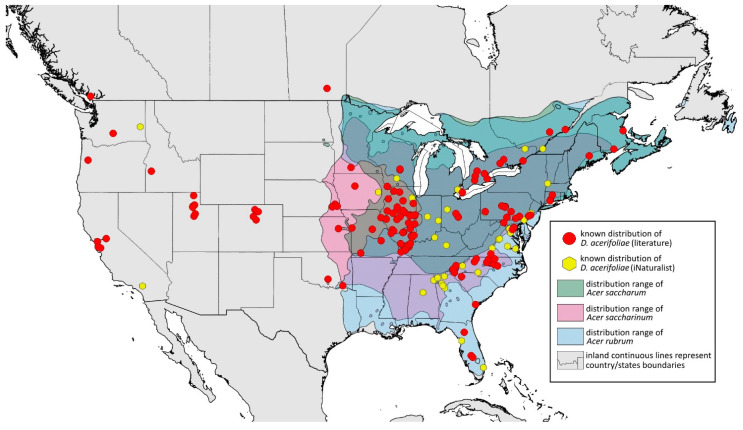
Known distribution of *Drepanaphis acerifoliae* in North America, with distribution ranges of its host plants.

Type species *Siphonophora acerifoliae* Thomas, 1878 by original designation
  Type species *Siphonophora acerifoliae* Thomas, 1878 by original designation
  = *Siphonophora acerifoliae* Thomas, 1878: 1(2): 4 [17]
  = *Siphonophora acericola* Thomas, 1878: 1(2): plate 1 (subsequent misspelling) [17]
  = *Drepanosiphum acerifolii* Monell, 1879: 5(1): 27 [40]
  = *Drepanosiphum acerifoliae* Davidson, 1909: 2(4): 303 [41]
  *Drepanaphis acerifoliae* Del Guercio, 1909: 2 4(4): 50 [16]
  = *Drepanaphis acerifolii* Davis, 1910: 3(5): 419 (subsequent misspelling) [42]
  = *Drepanaphis allegheneyensis* Miller, 1936: 68: 81 [43]
**Figure 1A, Figure 2A, Figure 3A, Figure 4A, Figure 5A, Figure 7A, Figure 8A, Figure 9A, Figure 10A, Figure 11A, Figure 11A, Figure 12A, Figure 13A, Figure 14A and Figure 15; Table 1, Table 2 and Table 3**
  **Material examined:** Paratype. Siphonophora acerifoliae Thomas, 757, Ft. Dodge, Iowa, Dubuque, Iowa, Peoria, Ill. Sept. I, 1887, SI. 7169, Ill. Nat.Hist. Sur.//Aphididae, Drepanaphis acerifoliae Thomas, See slide by Davis, Det. F. C. Hottes, Ill. Nat. Hist. Sur.//PARATYPE, Siphonophora acerifoliae Thomas//INHS Insect Collection 1058753—15 alate viv. fem. [INHS]. Lectotype. Aphididae, Drepanaphis acerifoliae (Thomas), acerifolae, Ft. Dodge + Dubuque, Iowa, also Peoria, Ill., 757, Sept. I, 1897, Det. F.C. Hottes, Ill. Nat. Hist Sur. ’28, SI 7168//Lecto-type, Siphonophora acerifoliae Thomas, Viviparous ♀ ♀//INHS, Insect Collection 457903—five alate viv. fem. [INHS].
  Additional material examined—Table S6.

**Alate viviparous female—re-description (n = 26)**
  **Colour. In life:** Head and thorax brown to dark brown with white wax stripes. Eyes red. Antennae pale with dark apices of ANT III–V. Fore femora darker than middle and hind femora, dark brown dorsally. Middle and hind femora pale brown to brown. Tibiae pale brown. Wing veins dark bordered, pterostigma dark brown. Abdomen covered by white wax dots. Tergites I–V brown, tergites I–II slightly lighter than tergites III–V. Tergites VI–VIII fully covered by wax. Siphunculi dark (Figure 8A).

**Pigmentation of mounted specimens:** Head and pronotum brown, rest of thorax dark brown (Figure 9A). ANT I–II brown, ANT III–VI pale brown with darker apices on ANT III–V and dark area with primary rhinarium on ANT VI. Pterostigma distinct, darkly pigmented, with small area inside without pigmentation (Figure 3A). Wing veins brown bordered (Figure 7A). Abdomen pale brown, marginal sclerites dark brown. DAT I pale at base, darker at tips; DAT II pale; DAT III–IV dark brown (Figure 1A). Siphunculi brown to dark brown; cauda, subgenital and anal plate pale. Fore femora darker dorsally (Figure 4A). Middle and hind femora brown with dark brown smudge. Tibiae brown with darker distal parts. Tarsi dark brown.

**Morphometric characters:** Head setae: two pairs of fronto-orbital setae, one pair of postero-dorsal setae, one pair of latero-dorsal setae on dorsal side, 0.04–0.05 mm long with pointed apices, one pair of pointed frontal setae on ventral side 0.09–0.1 mm long. ANT/BL 1.57–2.26; ANT/HW 9.23–13.74; PT/BASE 6.93–12.02. ANT III with 9–14 secondary rhinaria, BASE with 4 accessory rhinaria (Figure 2A), URS with 4–8 accessory setae (Figure 10A). Other ratios: ANT IV/ANT III 0.64–0.85; ANT V/ANT III 0.64–0.83; ANT VI/ANT III 1.12–1.74; URS/ANT III 0.08–0.13; URS/BASE 0.59–0.88; URS/SIPH 0.25–0.52; HT II/ANT III 0.09–0.14; HT II/BASE 0.62–0.9; TIBIA III/BL 0.49–0.73; SIPH/BL 0.1–0.15; SIPH/CAUDA 1.5–2.9. Dorsal abdominal segments with four pairs of distinct tubercles. DAT I 0.14–0.28 mm long, DAT II 0.07–0.14 mm long. DAT III largest, 0.23–0.37 mm long (Figure 11A). DAT IV smallest, 0.04–0.06 mm long. Pointed setae at ends of tubercles, 0.02–0.03 mm long. Dorsal setae with pointed apices, 0.04–0.06 mm long, on small sclerites on ABD I–V. Marginal sclerites with 3–10 setae. Siphunculi flask-shaped (Figure 5A).

**Oviparous female—re-description (n = 6)**
  **Colour. In life:** Head, thorax and abdomen reddish brown. Last segments of abdomen slightly darker. Marginal sclerites dark brown. Eyes red. Antennae dark brown. Siphunculi dark with lighter area on bases. Femora dark dorsally [10].
  **Pigmentation of mounted specimens:** Head brown, pronotum pale brown. ANT brown to dark brown with darker apices of segments. Cauda, subgenital and anal plate pale. Femora, tarsi and siphunculi brown. Tibiae dark brown with darker knee areas and distal parts. Abdominal sclerites brown (Figure 12A).

**Morphometric characters:** Head setae: two pairs of fronto-orbital setae, 0.1–0.12 mm long; one pair of latero-dorsal setae, one pair of postero-dorsal setae, 0.05–0.06 mm long with blunt apices on dorsal side; one pair of pointed frontal setae on ventral side, 0.1 mm long. ANT/BL 0.93–1.04. Other ratios: ANT VI/ANT III 1.28–1.83; PT/BASE 5.77–9.0; SIPH/BL 0.07–0.08; FEMUR III/BL 0.2–0.22; TIBIA III/BL 0.37–0.43; HT II/ANT VI 0.11–0.14; URS/ANT III 0.14–0.17; URS/BASE 0.71–0.92; URS/SIPH 0.44–0.5. ANT III without secondary rhinaria. URS with 4–8 accessory setae. Hind tibiae with 50–110 pseudosensoriae distributed along almost their entire lengths. Dorsal setae 0.07–0.14 mm long. Pleural and spinal setae on ABD I–V, placed on small dark sclerites. Marginal sclerites on ABD I–V bigger. Siphunculi tubular.
**Alate male—re-description (n = 3)**
  **Colour. In life:** Head and thorax brown to dark brown with white wax stripes. Eyes red. Antennae pale with dark apices of segments. Wing veins dark brown bordered. Abdomen covered by white wax dots. ABD I–II and VI–VII more intensively covered by wax. Siphunculi dark. Femora pale brown. Tibiae pale brown with darker knee areas.
  **Pigmentation of mounted specimens:** Head brown, thorax dark brown. Abdomen pale with brown spinal and marginal sclerites. ANT dark brown with darker apices of segments. ANT III slightly lighter at base. Wing veins brown bordered. Pterostigma distinct, darkly pigmented, with small area inside without pigmentation. Dorsal abdominal tubercles and siphunculi dark brown. Cauda and anal plate pale. Fore femora brown, darker dorsally. Middle and hind femora brown with dark brown smudge. Tibiae brown with darker knee areas and distal parts. Tarsi brown (Figure 13A).

**Morphometric characters:** Head setae: two pairs of fronto-orbital setae, one pair of postero-dorsal setae, one pair of latero-dorsal setae on dorsal side, 0.03–0.05 mm long with pointed apices; one pair of pointed frontal setae on ventral side, 0.07 mm long. ANT/BL 1.58. Other ratios: ANT VI/ANT III 1.4; PT/BASE 8.3; SIPH/BL 0.1–0.13; FEMUR III/BL 0.26–0.3; TIBIA III/BL 0.54–0.61; URS/ANT III 0.1–0.13; URS/SIPH 0.33–0.39. ANT III with 80–100 rhinaria, ANT IV with 38–50 rhinaria, ANT V with 17–21 rhinaria. URS with 6–8 accessory setae. DAT I inconspicuous or very small, 0.05–0.07 mm long. DAT III distinct, 0.15–0.18 mm long with setae 0.03 mm long at end. Dorsal setae 0.03–0.05 mm long, on small sclerites, bigger on ABD II–V. ABD IV–V with two spinal sclerites, each with two setae 0.03–0.04 mm long. Marginal sclerites with 3–10 setae 0.03–0.05 mm long. Siphunculi flask-shaped. Genitalia with basal part of phallus elongated, with broadly rounded apices (Figure 14A).

**Remarks:** The locus typicus is not designated since the type slides bear three locality names—Fort Dodge and Dubuque in Iowa and Peoria in Illinois (after Hottes and Frison [10], as well as Smith and Dillery [27]).
  **Host plants:** *Acer rubrum*, *Acer saccharinum*, *Acer saccharum*, also found on plantings of *Acer platanoides* (in North America), occasionally on *Acer nigrum*.
  **Distribution:** Canada: British Columbia (North Vancouver^); Manitoba (Winnipeg); New Brunswick (Middle Kouchibouguac‡, Rothesay‡); Ontario (Front of Yonge‡, Hamilton^, Havelock-Belmont-Methuen^, Leamington, London, North Perth‡, Perth South‡, Puslinch^, Smith-Ennismore-Lakefield‡); Quebec (Orsainville in Quebec City, Shawinigan (Lac Wapizagonke)‡). USA: California (Berkeley, Lodi, Palo Alto (vicinity of Stanford University), San Jose); Colorado (Boulder, Denver, Fort Collins, Greeley); Connecticut (Hamden, Hartford, New Haven); Florida (Gainesville, Highlands Hammock State Park, Lake Placid); Idaho (Eagle); Illinois (Albion, Alma*, Alton, Augerville, Bloomfield Precinct*, Cairo, Carbondale, Catlin, Danville, Dixon Springs, Edwardsville, Elizabethtown, Fairmount Township, Golconda, Grayville, Havana, Herod, Kankakee, Le Roy, Macomb, MURShall, Mattoon, Metropolis, Mount Carmel, Mount Carroll, Mount Pulaski*, Newton, Normal, Oregon, Pekin, Peoria, vicinity of Perry*, Rock Island, Quincy, Shawneetown, Starved Rock State Park, Springfield, Tonti, Urbana); Iowa (Dubuque, Fort Dodge); Kansas (Maple Hill*); Maine (Orono); Maryland (Beltsville, Laurel†); Minnesota (Saint James); Missouri (Columbia, Crane, Kansas City, Saint Louis, Steelville); Nebraska (Ashland, Lincoln, Weeping Water); North Carolina (Alamance, Tunnel Bypass Trail near Bryson City, Burlington, Chapel Hill, Cherokee, Franklin, Great Smoky Mountains National Park, Greensboro, Raleigh, Reidsville, Roaring Gap, Roxboro, Wilkesboro); Ohio (Columbus, Ostrander); Oregon (Corvallis); Pennsylvania (Bryn Athyn, Chambersburg, Houserville, Lancaster, Loganville, Miquon, New Bloomfield, Philipsburg, Pittsburgh, State College); South Carolina (Easley, Hardeeville); Tennessee (Cosby Horse Trail near Cosby); Utah (Bountiful, East Canyon Cashe Co., Logan, Payson, Provo, Salt Lake City); Virginia (Chatham); Washington (Yakima); Washington, D.C.; West Virginia (Martinsburg“); Wisconsin (Montello*, Shields in Marquette County). Europe: Hungary (Cegléd, Gazdagrét, Pesterzsébet, Tabán, Törökvész); Italy (Calendasco, Carlazzo, Milan (Bosco in Città, Sempione Parc), Nola); Serbia (Belgrade (Banjica, New Belgrade, Vrčin, Zemun near Danube), Novi Sad (Bistrica, Novo Naselje)); Spain (Astorga, León, Lleida) (Figure 15) ([10,17,21,23,24,27,41,44,45,46,47,48,49,50,51,52,53,54,55,56,57]; Centre for Biodiversity Genomics—Canadian Specimens [‡]; Illinois Natural History Survey Insect Collection [*]; International Barcode of Life project (iBOL) [^]; Natural History Museum of Denmark Entomology Collection [“]; new record in this publication [†]).

Additional distribution from iNaturalist (www.inaturalist.org, accessed on 12 June 2024): Canada: Ontario (LaSalle, Ottawa); Quebec (Dorval Island). USA: Alabama (Fort Payne, Hoover); California (Albany); Florida (Parkland); Georgia (Cashes Valley, Kennesaw, LaFayette); Illinois (New Lenox); Indiana (Zionsville); Iowa (Cedar Rapids); Kentucky (eastern outskirts of Louisville); Massachusetts (Williamstown); North Carolina (vicinity of Barnardsville, Charlotte, Durham); Ohio (Rest Area Southbound Wapakoneta); Pennsylvania (Bethel Park, Buckingham Springs, vicinity of Strickersville, Villanova University); Virginia (Dulles, Far Hills, Forest Lakes, Holly Knoll Cir near Great Falls, Great Falls Park, Herndon, Woodbridge); Wisconsin (Cross Plains). Europe: Spain (Santiago de Compostela).

#### 3.4.2. *Drepanaphis carolinensis* Smith, 1941

**Figure 16:**
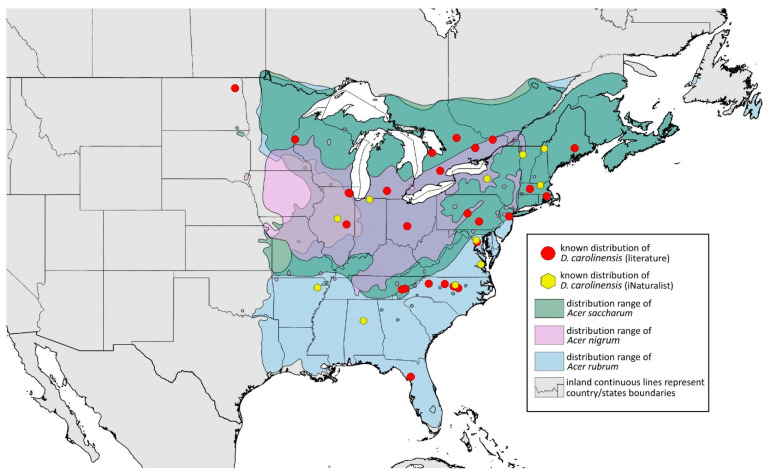
Known distribution of *Drepanaphis carolinensis* in North America, with distribution ranges of its host plants.

*Drepanaphis carolinensis* Smith, 1941: 57(2): 228, 231 [23]
**Figure 1B, Figure 3B, Figure 4B, Figure 5B, Figure 7B, Figure 8B, Figure 9B, Figure 11B, Figure 12B, Figure 13B, Figure 14B and Figure 16; Table 1, Table 2 and Table 3**
  **Material examined:** Holotype. Drepanaphis carolinensis Smith, Holotype Type No 55834. D.D.N.N.M.//N.C, Aphids, Host Acer, Raleigh, N.C. 193. Date 4–28-40. C.F. Smith—six alate viv. fem. (USNM) Paratype. Drepanaphis carolinensis Smith//N. C. Aphids, Host Maple, Raleigh, N.C. Date 4-26-40, 193, C. F. Smith//INHS, Insect Collection 1,058,855—four alate viv. fem. Paratype. Drepanaphis carolinensis Smith//N. C. Aphids, Host Acer, Raleigh, N.C. Date 4-30-40, 193, C. F. Smith//INHS, Insect Collection 1,058,858—four alate viv. fem. Paratype. Drepanaphis carolinensis Smith//N. C. Aphids, Host Sugar Maple, Milburnie, NC, Date 21 May 1940, C. F. Smith//INHS, Insect Collection 1,058,859—three alate viv. fem. Paratype. Drepanaphis carolinensis Smith//N. C. Aphids, Host Acer, Raleigh, N.C. Date 4-30-40, 193, C. F. Smith (IECA).
  Additional material examined—Table S6.
**Alate viviparous female—re-description (n = 16)**
  **Colour. In life:** Head, thorax and abdomen reddish brown. Head and pronotum with longitudinal white wax stripes. Abdomen covered by white wax dots, more intensively on ABD I–II and VI–VIII. Eyes red, antennal segments pale with dark apices. Wings clear with small area of dark brown pigmentation at end, radius veins brown. Pterostigma brown. Femora and siphunculi dark. Tibiae pale. DAT dark brown (Figure 8B).
  **Pigmentation of mounted specimens:** Head, pronotum, ANT I–II brown. Rest of thorax dark brown (Figure 9B). ANT III–VI pale brown with darker apices on ANT III–V and dark area with primary rhinarium on ANT VI. Wings clear with small area of dark brown pigmentation at end, radius veins dark brown (Figure 7B). Pterostigma distinct, darkly pigmented, with small area inside without pigmentation (Figure 3B). Abdomen pale brown, marginal sclerites dark brown. DAT (Figure 1B) and siphunculi dark brown. Cauda, subgenital and anal plate pale. Fore femora brown darker dorsally (Figure 4B). Middle and hind femora brown with darker smudge. Hind femora with darker stripes at margins. Tibiae brown with darker knee areas and distal parts. Tarsi brown.
  **Morphometric characters:** Head setae: two pairs of fronto-orbital setae, one pair of postero-dorsal setae, one pair of latero-dorsal setae on dorsal side, 0.02–0.04 mm long with pointed apices; one pair of pointed frontal setae on ventral side 0.07–0.08 mm long. ANT/BL 1.44–2.07; ANT/HW 9.3–13.25; PT/BASE 5.56–8.1. ANT III with 9–15 secondary rhinaria, BASE with 4 accessory rhinaria, URS with 4–8 accessory setae. Other ratios: ANT IV/ANT III 0.64–0.82; ANT V/ANT III 0.62–0.75; ANT VI/ANT III 1.02–1.6; URS/ANT III 0.09–0.12; URS/BASE 0.56–0.71; URS/SIHP 0.38–0.56; HT II/ANT III 0.11–0.14; HT II/BASE 0.6–0.89; TIBIA III/BL 0.5–0.66; SIPH/BL 0.08–0.14; SIPH/CAUDA 1.33–2.63. DAT with four pairs of distinct tubercles. DAT I 0.13–0.16 mm long, slightly larger than DAT II 0.1–0.13 mm long. DAT III largest, 0.23–0.32 mm long (Figure 11B). DAT IV smallest, 0.06–0.1 mm long. Dorsal setae with pointed apices, 0.02–0.04 mm long, on small sclerites on ABD I–V. Marginal sclerites with 3–6 setae. Siphunculi flask-shaped (Figure 5B).
**Oviparous female—description (n = 8)**
  **Colour. In life:** Unknown.
  **Pigmentation of mounted specimens:** Head and thorax brown, abdomen pale. ANT I–II brown. ANT III–VI pale brown with darker apices on ANT III–V. Siphunculi, subgenital, anal plate and cauda brown. Fore, middle and hind femora dark brown. Tibiae pale brown with darker knee areas and distal parts. Tarsi pale brown. Dorsal sclerites brown (Figure 12B).
  **Morphometric characters:** Head setae: two pairs of fronto-orbital setae, 0.07–0.1 mm long; one pair of postero-dorsal setae, 0.05–0.06 mm long; one pair of latero-dorsal setae, 0.03–0.04 mm long with blunt apices on dorsal side; one pair of pointed frontal setae on ventral side, 0.09–0.1 mm long. ANT/BL 0.94–1.42. Other ratios: ANT VI/ANT III 1.5–2.0; PT/BASE 5.14–7.0; SIPH/BL 0.06–0.08; FEMUR III/BL 0.2–0.26; TIBIA III/BL 0.38–0.5; HT II/ANT VI 0.1–0.14; URS/ANT III 0.14–0.17; URS/BASE 0.64–0.71; URS/SIPH 0.5–0.64. ANT III with one or without secondary rhinaria. URS with 4–8 accessory setae. Hind tibiae with 27–54 pseudosensoria distributed along almost their entire length. Dorsal setae 0.04–0.1 mm long. Marginal sclerites on ABD I–V distinct. Siphunculi tubular.
**Alate male—re-description (n = 3)**
  **Colour. In life:** Unknown.
  **Pigmentation of mounted specimens:** Head, ANT I–II, IV–VI brown; ANT III light brown with darker apices. Thorax, DAT and siphunculi dark brown. Abdominal sclerotisation dark brown. Wings clear with small area of dark brown pigmentation at end, radius veins brown. Pterostigma distinct, darkly pigmented, with small area inside without pigmentation. Cauda and anal plate pale. Fore femora brown darker dorsally. Middle and hind femora brown with darker smudge. Hind femora with darker stripes at margins. Tibiae brown with darker knee areas and distal parts. Tarsi brown (Figure 13B).
  **Morphometric characters:** Head setae: two pairs of fronto-orbital setae, one pair of postero-dorsal setae, one pair of latero-dorsal setae, 0.02–0.03 mm long on dorsal side; one pair of frontal setae on ventral side, 0.05 mm long. ANT/BL 1.53–1.55. Other ratios: ANT VI/ANT III 1.08–1.42; PT/BASE 6.64–7.67; SIPH/BL 0.09–0.1; FEMUR III/BL 0.25–0.3; TIBIA III/BL 0.53–0.63; URS/ANT III 0.09–0.1; URS/SIPH 0.45–0.5. ANT III with 69–102 rhinaria, ANT IV with 43–56 rhinaria, ANT V with 25–38 rhinaria. URS with 4–6 accessory setae. DAT I and II inconspicuous or very small, 0.03–0.04 mm long. DAT III distinct, 0.1–0.15 mm long; DAT IV on spinal sclerites, 0.04–0.05 mm long. Dorsal setae on abdomen with pointed apices, 0.02–0.03 mm long, on small sclerites. Spinal sclerites on ABD V with 4–5 setae, 0.03–0.04 mm long. Marginal sclerites with 3–6 setae. Siphunculi flask-shaped. Genitalia with basal part of phallus short, hook-shaped (Figure 14B).
  **Host plants:***Acer saccharum*, occasionally on *Acer nigrum* and *Acer rubrum*.
  **Distribution:** Canada: Ontario (Algonquin Provincial Park^, Bon Echo Provincial Park^, Guelph (Clairfields)^, Ottawa^, Owen Sound^). USA: Florida (Waccasassa River in Levy County); Illinois (Arlington Heights*, Palatine*, Urbana*); Maine (Orono); Massachusetts (Amherst, Taunton); Minnesota (Walsh); Michigan (Albion); New Jersey (Rahway†); North Carolina (Chapel Hill, Greensboro, Moravian Falls, Raleigh—locus typicus); Ohio (Columbus); Pennsylvania (Harrisburg, State College); Tennessee (Gatlinburg^, Great Smoky Mountains National Park); Washington, D.C.; Wisconsin (Saint Croix Falls) (Figure 16) ([23,24,27,58]; Illinois Natural History Survey Insect Collection [*]; International Barcode of Life project (iBOL) [^]; new record in this publication [†]).

Additional distribution from iNaturalist (www.inaturalist.org, accessed on 12 June 2024): USA: Alabama (Birmingham Botanical Gardens); Arkansas (Jonesboro); Illinois (Bloomington); Indiana (South Bend); Maryland (North Bethesda); Massachusetts (Groton); New Hampshire (Dixville); New York (Onondaga); North Carolina (Durham (Trinity Park)); Vermont (Essex Junction); Virginia (Williamsburg (York River State Park)).

#### 3.4.3. *Drepanaphis choanotricha* Smith & Dillery, 1968

**Figure 17:**
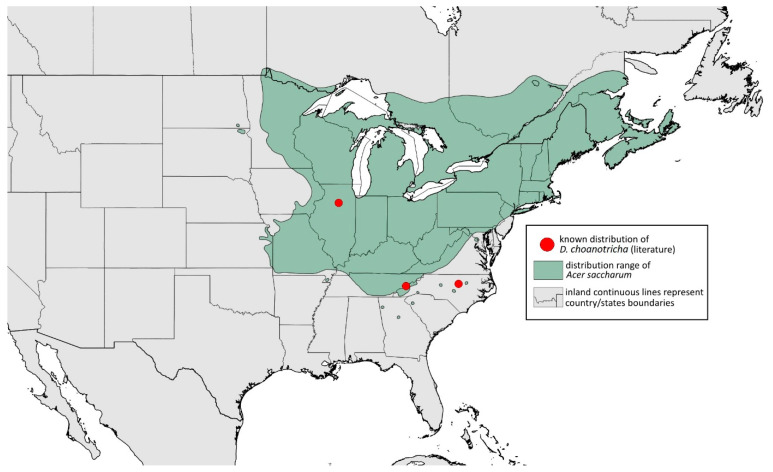
Known distribution of *Drepanaphis choanotricha* in North America, with distribution ranges of its host plants.

*Drepanaphis choanotricha* Smith & Dillery, 1968: 61(1): 186, 190 [27]
**Figure 1C, Figure 2B, Figure 3C, Figure 4C, Figure 5C, Figure 9C, Figure 11C, Figure 12C, Figure 13C, Figure 14C and Figure 17; Table 1, Table 2 and Table 3**
  **Material examined:** Holotype. Drepanaphis choanotricha Smith & Dillery Det. Smith & Dillery, 60-1060 Southern sugar maple, Paratype (blue)//Umstead PK. Raleigh, N. C. 9•11•60, Holotype Red, CFS—two alate viv. fem. [USNM] Paratype. Drepanaphis choanotricha Smith & Dillery Det. Smith & Dillery, 60-1060 Southern Sugar maple//Umstead PK. Raleigh, N. C. 9•11•60, Paratype//INHS, Insect Collection 1058862—four alate viv. fem. Paratype. Drepanaphis choanotricha Smith & Dillery, paratype, BM 1984-340, Det: Smith & Dillery//N.U.S.A, Pl. Southern Sugar maple, Loc. Umstead Pk, Raleigh, N.C., Date II.IX.1960, Leg. C. F. Smith, 60.1060//NHMUK 014314711—three alate viv. fem. Paratype. Drepanaphis choanotricha Smith & Dillery Det. Smith & Dillery, 60-1060 Southern Sugar maple//Umstead PK. Raleigh, N. C. 9•11•60, Paratype//08107//Museum Paris MNHN 25145—three alate viv. fem.
  Additional material examined—Table S6.
**Alate viviparous female–re-description (n = 13)**
  **Colour. In life:** Black with pale legs. Head bearing three longitudinal and one anterior transverse white wax stripes. Pronotum with two or three medial longitudinal stripes. Mesonotum with one pair of latero-anterior and one pair of medio-posterior wax dots. Metanotum with one pair of lateral wax dots. Abdomen with rows of white wax dots, denser at posterior end [27].
  **Pigmentation of mounted specimens**: Head, ANT I, II, thorax dorsal abdominal tubercles and siphunculi dark brown (Figure 1C and Figure 9C). ANT III–VI pale brown with darker apices on ANT III–V and dark area with primary rhinarium on ANT VI. Wings clear, with dark brown pigmentation of end of veins, radius veins brown. Pterostigma distinct, darkly pigmented, oval with small area inside without pigmentation (Figure 3C). Abdomen pale, dorsal sclerotisation brown. Cauda, subgenital and anal plate pale. Fore femora pale brown (Figure 4C).
  **Morphometric characters:** Head setae: two pairs of fronto-orbital setae, one pair of postero-dorsal setae, one pair of latero-dorsal setae on dorsal side, 0.02–0.05 mm long with blunt apices; one pair of pointed frontal setae on ventral side, 0.06–0.08 mm long. ANT/BL 1.79–3.35; ANT/HW 11.73–19.99; PT/BASE 9.04–16.31. ANT III with 2–5 secondary rhinaria, BASE with 5–6 accessory rhinaria (Figure 2B). URS with 6–10 accessory setae. Other ratios: ANT IV/ANT III 0.61–0.99; ANT V/ANT III 0.67–0.83; ANT VI/ANT III 1.81–3.48; URS/ANT III 0.12–0.16; URS/BASE 0.69–0.86; URS/SIPH 0.5–0.76; HT II/ANT III 0.11–0.15; HT II/BASE 0.58–0.79; TIBIA III/BL 0.57–0.74; SIPH/BL 0.1–0.15; SIPH/CAUDA 2.06–4.02. DAT II–IV clearly visible, DAT I inconspicuous. DAT II 0.04–0.06 mm long, DAT III 0.13–0.24 mm long (Figure 11C). DAT IV smallest, 0.01–0.03 mm long. Setae at ends of tubercles 0.03–0.04 mm long with blunt apices. Marginal sclerites with 2–4 blunt setae 0.02–0.04 mm long. Distinct spinal setae 0.04–0.05 mm long with blunt apices on small sclerites. Siphunculi flask-shaped (Figure 5C).
**Oviparous female—description (n = 2)**
  **Colour. In life:** Unknown.
  **Pigmentation of mounted specimens:** Head, thorax and coxa brown. ANT I, II and siphunculi dark brown. ANT III–VI pale brown with darker apices on ANT III–V. Abdomen pale. Dorsal sclerotisation brown. Fore, middle and hind femora; tarsi subgenital; anal plate; and cauda pale brown (Figure 12C).
  **Morphometric characters**: Head setae: two pairs of fronto-orbital setae, 0.1–0.12 mm long; one pair of postero-dorsal setae, one pair of latero-dorsal setae on dorsal side, 0.06–0.08 mm long with blunt apices; one pair of pointed frontal setae on ventral side, 0.1 mm long. ANT/BL 1.67–1.93. Other ratios: ANT VI/ANT III 2.34–2.7; PT/BASE 12.25–13.3; SIPH/BL 0.12; FEMUR III/BL 0.27–0.29; TIBIA III/BL 0.48–0.51; HT II/ANT VI 0.05–0.06; URS/ANT III 0.13–0.16; URS/BASE 0.75–0.83; URS/SIPH 0.38–0.48. ANT III without secondary rhinaria. URS with 6–7 accessory setae. Hind tibiae with 23–29 pseudosensoria, more abundant in distal parts of tibiae. Dorsal setae 0.1–0.13 mm long. ABD I–VI with setae on small dark sclerites. ABD I–V with marginal sclerites. Siphunculi flask-shaped.
**Alate male—re-description (n = 1)**
  **Colour. In life:** Unknown.
  **Pigmentation of mounted specimens:** Head, ANT I, II, thorax, coxa, dorsal abdominal tubercles and siphunculi dark brown. ANT III–VI pale brown with darker apices on ANT III–V. Wings clear with distinct area of dark brown pigmentation at end, radius veins brown. Pterostigma distinct, darkly pigmented, with small area inside without pigmentation. Abdomen pale, dorsal sclerotisation brown. Cauda and anal plate brown. Fore, middle, hind femora and tarsi pale brown (Figure 13C).
  **Morphometric characters:** Head setae: two pairs of fronto-orbital setae, one pair of postero-dorsal setae, one pair of latero-dorsal setae on dorsal side, 0.03–0.04 mm long with pointed apices; one pair of pointed frontal setae on ventral side, 0.07–0.08 mm long. ANT/BL 2.34–2.36. Other ratios: ANT VI/ANT III 2.35–2.51; PT/BASE 12.9–13.4; SIPH/BL 0.12; FEMUR III/BL 0.28–0.31; TIBIA III/BL 0.58–0.59; URS/ANT III 0.13; URS/SIPH 0.53. ANT III with 43–44 rhinaria, ANT IV with 21–23 rhinaria, ANT V with 16–18 rhinaria. BASE with seven very small accessory rhinaria. URS with six accessory setae. DAT III 0.05 mm long with pointed setae 0.02 mm long at end. Dorsal setae 0.02–0.04 mm long with pointed apices. Spinal sclerites on ABD IV with two setae, ABD V with one seta. Marginal sclerites with 3–6 setae, spinal setae on small sclerites. Siphunculi flask-shaped. Genitalia with basal part of phallus elongated, robust, with pilled inner edges (Figure 14C).
  **Host plant:** *Acer saccharum*.
  **Distribution:** USA: Illinois (Starved Rock State Park); North Carolina (Raleigh (William B. Umstead State Park)—locus typicus); Tennessee (along Low Gap Trail in Great Smoky Mountains National Park*) (Figure 17) ([27]; Illinois Natural History Survey Insect Collection [*]).

#### 3.4.4. *Drepanaphis granovskyi* Smith & Knowlton, 1943

**Figure 18:**
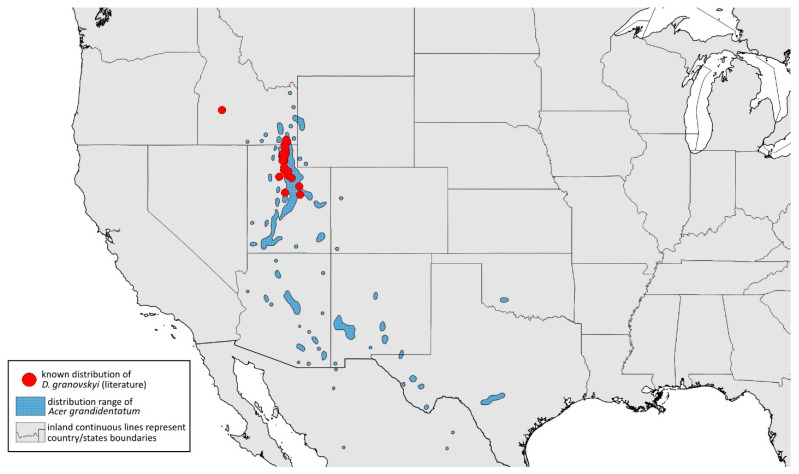
Known distribution of *Drepanaphis granovskyi* in North America, with distribution ranges of its host plants.

*Drepanaphis granovskyi* Smith & Knowlton, 1943: 59(2): 172, 173 [24]
**Figure 1D, Figure 3D, Figure 4D, Figure 5D, Figure 9D, Figure 11D, Figure 12D, Figure 13D, Figure 14D and Figure 18; Table 1, Table 3 and Table 4**
  **Material examined:** Type. Drepanaphis granovskyi S-K//Mt. Acer grandidentatum, Liberty, Ut., Aug. 13, 1942, GF. Knowlton—three alate viv. fem. (USNM) Paratype. Drepanaphis granovskyi S-K//Mt. *Acer grandidentatum*, Spanish Fork, Ut., Aug. 10, 1942, Whitish freen, C F Knowlton//INHS, Insect Collection 1058866—three alate viv. fem.
  Additional material examined—Table S6.
**Alate viviparous female—re-description (n = 13)**
  **Colour. In life:** Pale white, appendages clear; without conspicuous wax [27].
  **Pigmentation of mounted specimens:** Head, thorax, ANT pale brown (Figure 9D). Wings clear with palely pigmented pterostigma, with large area inside without pigmentation (Figure 3D). Abdomen, dorsal abdominal tubercles (Figure 1D) and siphunculi pale. Fore femora pale brown (Figure 4D).
  **Morphometric characters:** Head setae: two pairs of pointed fronto-orbital setae, 0.02–0.05 mm long; one pair of postero-dorsal setae, one pair of latero-dorsal setae on dorsal side, 0.01–0.02 mm long with pointed apices; two pairs of pointed frontal setae on ventral side, 0.02–0.05 mm long. ANT/BL 1.23–1.47; ANT/HW 8.19–10.79; PT/BASE 3.48–8.08. ANT III with 9–13 secondary rhinaria, BASE with 4 accessory rhinaria. URS with 4–6 accessory setae. Other ratios: ANT IV/ANT III 0.55–0.72; ANT V/ANT III 0.5–0.75; ANT VI/ANT III 0.81–1.51; URS/ANT III 0.1–0.14; URS/BASE 0.57–0.96; URS/SIPH 0.33–0.54; HT II/ANT III 0.11–0.18; HT II/BASE 0.82–1.0; TIBIA III/BL 0.46–0.54; SIPH/BL 0.08–0.12; SIPH/CAUDA 1.17–2.62. Dorsal abdominal segments with distinct three pairs of tubercles. DAT I biggest, 0.09–0.12 mm long; DAT II 0.04–0.06 mm long; DAT III 0.03–0.06 mm long. DAT IV inconspicuous (Figure 1D and Figure 11D). Setae at ends of tubercles, 0.02–0.03 mm long. Abdominal dorsal setae 0.02–0.04 mm long with pointed apices. Siphunculi tubular (Figure 5D).
**Oviparous female—description (n = 1)**
  **Colour. In life:** Unknown.
  **Pigmentation of mounted specimens:** Body in general pale brown or with slightly darker hind tibiae and slightly lighter abdomen (Figure 12D).
  **Morphometric characters:** Head setae: two pairs of fronto-orbital setae, 0.07–0.09 mm long; one pair of postero-dorsal setae, 0.09 mm long; one pair of latero-dorsal setae, 0.05–0.06 mm long on dorsal side with forked apices. Two pairs of pointed frontal setae on ventral side, 0.04–0.06 mm long. ANT/BL 0.89–0.91. Other ratios: ANT VI/ANT III 1.5–1.83; PT/BASE 5.0–5.82; SIPH/BL 0.08–0.093; FEMUR III/BL 0.2–0.21; TIBIA III/BL 0.39; URS/ANT III 0.19; URS/BASE 0.73; URS/SIPH 0.4. ANT III without secondary rhinaria. URS with four accessory setae. Hind tibiae with 42–45 pseudosensoria, more abundant in middle part and on ends of tibiae. Dorsal setae 0.08–0.11 mm long with forked apices. Siphunculi tubular.
**Alate male—description (n = 1)**
  **Colour. In life:** Unknown.
  **Pigmentation of mounted specimens:** Head, thorax, ANT I dark brown. Pronotum, ANT II, III pale brown; ANT IV–VI brown. Wings clear, pterostigma distinct, darkly pigmented, with small area inside without pigmentation. Abdomen pale, spinal sclerites and siphunculi dark brown. Cauda and anal plate dark brown. Fore femora brown (Figure 13D).
  **Morphometric characters:** Head setae: two pairs of fronto-orbital setae, one pair of postero-dorsal setae, one pair of latero-dorsal setae on dorsal side, two pairs of frontal setae on ventral side, 0.02–0.04 mm long with pointed apices. ANT/BL 0.96. Other ratios: SIPH/BL 0.09; URS/SIPH 0.47. ANT III with 66–68 rhinaria, ANT IV with 34–35 rhinaria, ANT V with 19–20 rhinaria. URS with four accessory setae. DAT absent. Dorsal setae 0.02–0.03 mm long with pointed apices. Spinal sclerites on ABD II–V with two pointed setae 0.02 mm long. Marginal sclerites with 1–3 setae, most distinct on ABD IV. Siphunculi tubular. Genitalia with basal part of phallus short, robust, rectangular (Figure 14D).
  **Host plant:** *Acer grandidentatum*.
  **Distribution:** USA: Idaho (Birch Creek (Cub River Canyon), Franklin, Mink Creek, Strawberry Creek); Utah (Avon Canyon, Beaver Canyon, Big Cottonwood Canyon, Blacksmith Fork Canyon, Bountiful, Brigham Canyon, East Canyon (Cache County), Eden, Farmington Canyon, Green Canyon, Heber, Liberty—locus typicus, Logan Canyon, Mantua, Mount Nebo, North Ogden, Richmond, Rolapp, Sardine Canyon, Wellsville Canyon, Willow Creek) (Figure 18) [24,27].

#### 3.4.5. *Drepanaphis idahoensis* Smith & Dillery, 1968

**Figure 19:**
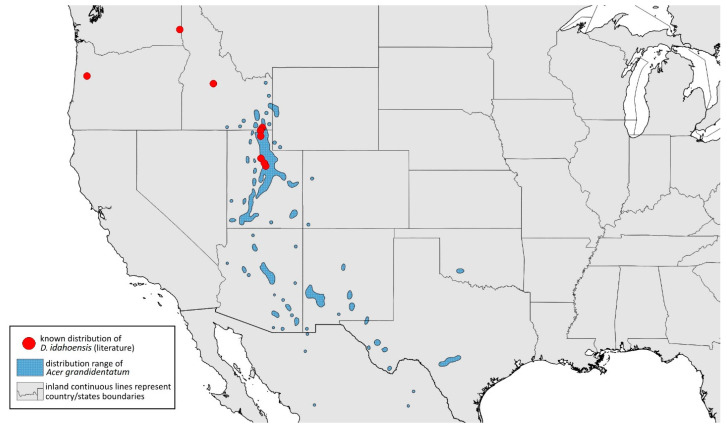
Known distribution of *Drepanaphis idahoensis* in North America, with distribution ranges of its host plants.

*Drepanaphis idahoensis* Smith & Dillery, 1968: 61(1): 186, 193 [27]
**Figure 1E, Figure 3E, Figure 4E, Figure 5E, Figure 9E, Figure 11E, Figure 12E and Figure 19; Table 1 and Table 2**
  **Material examined:** Holotype. Drepanaphis idahoensis Smith & Dillery Det. Smith & Dillery, 60-887, Acer grandidentatum, Paratype (blue)//Cub River Cany, Ida., 8•16•60, Al. dK transverse area between cornicles, Holotype (red) K-S—two alate viv. fem. (USNM) Paratype. Drepanaphis idahoensis Smith & Dillery Det. Smith & Dillery, 60-887, Acer grandidentatum, Paratype//Cub River Cany, Ida., 8•16•60, Al. dK transverse area between cornicles//Museum Paris MNHN 25147—two alate viv. fem.
**Additional material examined—Table S6.**
**Alate viviparous female—re-description (n = 10)**
  **Colour. In life:** Body entirely frosted with white wax except for dark, U-shaped line more or less connecting DAT III to siphunculi. Legs pale [27].
  **Pigmentation of mounted specimens:** Head, thorax, ANT I brown (Figure 9E). ANT II–VI pale brown with slightly darker apices on ANT III–V and dark area with primary rhinarium on ANT VI. Wings clear with distinct area of dark brown pigmentation at end of veins. Pterostigma distinct, darkly pigmented, with small area inside without pigmentation (Figure 3E). Abdomen pale with brown abdominal sclerites. Dorsal abdominal tubercles (Figure 1E) and siphunculi dark brown. Cauda, subgenital and anal plate pale. Fore femora pale brown (Figure 4E).
  **Morphometric characters:** Head setae: two pairs of fronto-orbital setae, one pair of postero-dorsal setae, one pair of latero-dorsal setae on dorsal side, 0.02–0.03 mm long with blunt apices; one pair of pointed frontal setae on ventral side, 0.05–0.06 mm long. ANT/BL 1.8–2.54; ANT/HW 11.65–20.98; PT/BASE 5.31–11.99. ANT III with 6–9 secondary rhinaria, BASE with 4 accessory rhinaria. URS with 6–8 accessory setae. Other ratios: ANT IV/ANT III 0.70–0.85; ANT V/ANT III 0.68–0.99; ANT VI/ANT III 0.83–2.02; URS/ANT III 0.09–0.11; URS/BASE 0.58–0.75; URS/SIPH 0.34–0.49; HT II/ANT III 0.08–0.12; HT II/BASE 0.6–0.86; TIBIA III/BL 0.6–0.81; SIPH/BL 0.11–0.16; SIPH/CAUDA 1.7–2.45. DAT I 0.03–0.06 mm long; DAT II 0.05–0.1 mm long; DAT III biggest, 0.18–0.26 mm long. DAT IV inconspicuous (Figure 11E). Dorsal setae 0.02–0.03 mm long with blunt apices, on ABD I–V on small sclerites. Marginal sclerites with 2–4 blunt setae. Siphunculi flask-shaped (Figure 5E).
**Oviparous female—description (n = 1)**
  **Colour. In life:** Unknown.
  **Pigmentation of mounted specimens:** Body in general pale brown or yellowish with slightly lighter abdomen (Figure 12E).
  **Morphometric characters:** Head setae: two pairs of blunt fronto-orbital setae, 0.09–0.1 mm long; one pair of blunt postero-dorsal setae, 0.08 mm long; one pair of blunt latero-dorsal setae on dorsal side, 0.04 mm long; one pair of pointed frontal setae on ventral side, 0.07 mm long. ANT/BL 1.23–1.24. Other ratios: ANT VI/ANT III 0.86–0.92; PT/BASE 3.92–4.58; SIPH/BL 0.08–0.09; FEMUR III/BL 0.25–0.26; TIBIA III/BL 0.51; HT II/ANT VI 0.13–0.16; URS/ANT III 0.14; URS/BASE 0.77; URS/SIPH 0.53. ANT III without secondary rhinaria. URS with eight accessory setae. Hind tibiae with 22–23 pseudosensoria abundant, distributed in central part of tibiae. Dorsal setae 0.08–0.11 mm long. Siphunculi tubular.
  **Male:** Unknown.
  **Remarks:** Some specimens from Lawrence in Kansas come from the collection of the J.B. Wallis/R.E. Roughley Museum of Entomology, Canada, and the Museum of Zoology, Lund University, Sweden. However, we did not have the opportunity to verify the slides and confirm whether they are indeed individuals representing this species. Moreover, *Acer nigrum* is listed as a host plant on those slides.
  **Host plant:** *Acer grandidentatum*, occasionally found on *Acer negundo* and *Acer saccharum*.
  **Distribution:** USA: Idaho (Cub River Canyon—locus typicus, Franklin, Stanley (between Thompson Creek and Tennell Creek‴)); Oregon (Corvallis (Benton County)); Utah (Cub River Canyon, Hobble Creek Canyon, Providence, Salt Lake City, Vivian Park in Provo Canyon); Washington (Pullman (Whitman County)) (Figure 19) ([27]; NMNH Extant Specimen Records (USNM, US) [‴]).

#### 3.4.6. *Drepanaphis kanzensis* Smith, 1941

**Figure 20:**
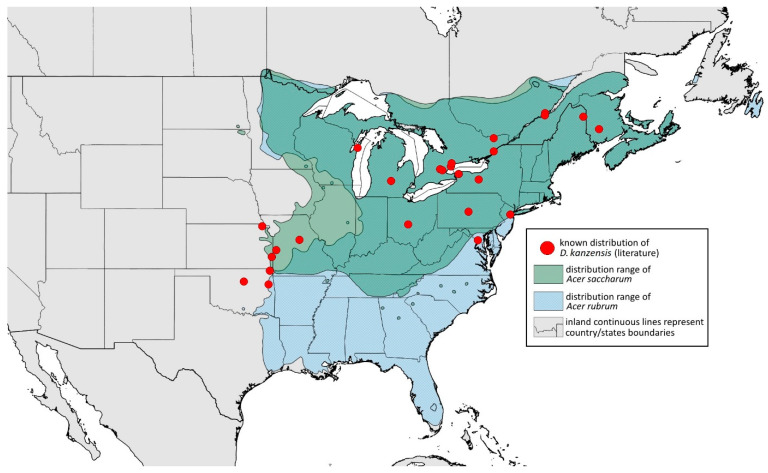
Known distribution of *Drepanaphis kanzensis* in North America, with distribution ranges of its host plants.

*Drepanaphis kanzensis* Smith, 1941: 72(2): 228, 232 [23]
  = *Drepanaphis kansensis* Leonard, 1959: 32(1): 12 [59]
**Figure 1F, Figure 3F, Figure 4F, Figure 5F, Figure 9F, Figure 11F, Figure 12F, Figure 13E, Figure 14E and Figure 20; Table 1, Table 2 and Table 3**
  **Material examined:** Drepanaphis kanzensis Smith Holotype Type No 55838. D.D.N.N.M.//Ka. Aphids, Host Sugar maple, Ft. Scott, Date 6-17 1940, C.F. Smith. Type—five alate viv. fem. (USNM) Paracotype. Drepanaphis kanzensis C. F. Smith//Ka. Aphids, Host Sugar maple, Ft. Scott, Ka, Date 6-17-40, C.F. Smith//08109//Museum Paris MNHN 25148—five alate viv. fem.
  Additional material examined—Table S6.
**Alate viviparous female—re-description (n = 17)**
  **Colour. In life:** Head, thorax and abdomen covered by white wax. ANT and legs pale, eyes red. Dorsal abdominal tubercles dark brown. Wings clear with pale pterostigma.
  **Pigmentation of mounted specimens:** Head and pronotum brown, rest of thorax dark brown (Figure 9F). ANT I–II brown, ANT III–VI pale brown with darker apices on ANT III–V and dark area with primary rhinarium on ANT VI. Wings clear with palely pigmented pterostigma, with large area inside without pigmentation (Figure 3F). Abdomen pale with brown sclerites. Dorsal abdominal tubercles dark brown (Figure 1F). Siphunculi pale brown with darker smudge. Cauda, subgenital and anal plate pale. Fore femora pale brown (Figure 4F).
  **Morphometric characters:** Head setae: two pairs of fronto-orbital setae, one pair of postero-dorsal setae, one pair of latero-dorsal setae on dorsal side, 0.03–0.05 mm long with pointed apices; one pair of pointed frontal setae on ventral side, 0.08–0.09 mm long. ANT/BL 1.44–2.56; ANT/HW 9.35–14.43; PT/BASE 5.6–12.98. ANT III with 10–15 secondary rhinaria, BASE with 4 accessory rhinaria. URS with 6–8 accessory setae. Other ratios: ANT IV/ANT III 0.6–0.8; ANT V/ANT III 0.59–0.84; ANT VI/ANT III 0.76–1.086; URS/ANT III 0.087–0.108; URS/BASE 0.56–0.68; URS/SIPH 0.38–0.63; HT II/ANT III 0.16–0.24; HT II/BASE 0.59–0.87; TIBIA III/BL 0.52–0.73; SIPH/BL 0.084–0.137; SIPH/CAUDA 0.94–2.33. Dorsal abdominal segments with distinct three pairs of tubercles. DAT I inconspicuous. DAT II 0.09–0.11 mm long, DAT III biggest 0.2–0.28 mm long (Figure 11F), DAT IV smallest 0.05–0.06 mm long. Pointed setae at end of tubercles 0.03–0.04 mm long. Dorsal setae 0.04–0.05 mm long with pointed apices, on small sclerites. Marginal sclerites with 2–5 setae. ABD VIII with two spinal sclerites, each with two setae. Siphunculi flask-shaped (Figure 5F).
**Oviparous female—description (n = 5)**
  **Colour. In life:** Unknown.
  **Pigmentation of mounted specimens:** Head, thorax, ANT pale brown. ANT with darker apices on ANT III–V. Abdomen and siphunculi pale. Dorsal sclerotisation pale brown. Femora, tibiae pale brown. Tibiae with slightly darker apical areas. Tarsi brown (Figure 12F).
  **Morphometric characters:** Head setae: two pairs of fronto-orbital setae, one pair of postero-dorsal setae, one pair of latero-dorsal setae on dorsal side, 0.07–0.1 mm long with blunt apices; one pair of pointed frontal setae on ventral side, 0.1 mm long. ANT/BL 0.75–1.4; PT/BASE 7.43–10.66. Other ratios: ANT VI/ANT III 1.51–2.17; SIPH/BL 0.05–0.07; FEMUR III/BL 0.21–0.28; TIBIA III/BL 0.38–0.52; HT II/ANT VI 0.07–0.1; URS/ANT III 0.098–0.13; URS/BASE 0.62–0.69; URS/SIPH 0.44–0.53. ANT III without secondary rhinaria. URS with eight accessory setae. Hind tibiae with 41–67 pseudosensoria distributed in central part of tibiae. Dorsal setae 0.09–0.13 mm long. Siphunculi tubular.
**Alate male—description (n = 3)**
  **Colour. In life:** Unknown.
  **Pigmentation of mounted specimens:** ANT pale with darker apices on ANT III–V and dark area with primary rhinarium on ANT VI. Wings clear with palely pigmented pterostigma, with large area inside without pigmentation. Abdomen pale with brown to dark brown sclerotisation. Siphunculi pale; cauda and anal plate brown. Legs pale, tibiae with slightly darker apical parts (Figure 13E).
  **Morphometric characters:** Head setae: two pairs of fronto-orbital setae, one pair of postero-dorsal setae, one pair of latero-dorsal setae on dorsal side, 0.03–0.04 mm long with pointed apices; one pair of pointed frontal setae on ventral side, 0.05–0.06 mm long. ANT/BL 1.45–2.36. Other ratios: ANT VI/ANT III 0.92–1.5; PT/BASE 6.38–10.0; SIPH/BL 0.09–0.11; FEMUR III/BL 0.28–0.33; TIBIA III/BL 0.54–0.67; URS/ANT III 0.08–0.09; URS/SIPH 0.35–0.42. ANT III with 94–127 rhinaria, ANT IV with 38–62 rhinaria, ANT V with 18–32 rhinaria. URS with six accessory setae. DAT III 0.13–0.15 mm long with pointed setae 0.03 mm long at end. DAT I, II and III inconspicuous. Dorsal setae 0.03–0.04 mm long with pointed apices. Spinal sclerites with two setae on ABD I, V, VI, VII, ABD VIII with four setae. Marginal sclerites with 3–4 setae. Siphunculi tubular. Genitalia with basal part of phallus elongated, triangular (Figure 14E).
  **Remarks:** One slide with two individuals, previously misidentified as *Drepanaphis kanzensis* from the NHMUK collection (BM 1958-454; NHMUK 014314720) is correctly identified as *Drepanaphis idahoensis*. The material was collected in Hobble Creek Canyon, Utah, USA, from *Acer grandidentatum*.
  **Host plant:** *Acer rubrum*, *Acer saccharum*.
  **Distribution:** Canada: New Brunswick (Fredericton); Ontario (Brockville°, Guelph°, Ottawa, Puslinch, Toronto°, Unionville); Quebec (Orsainville (Zoo), Sainte-Foy). USA: Kansas (Fort Scott—locus typicus, Hiawatha); Maine (Presque Isle); Michigan (East Lansing); Missouri (Butler, Columbia); New Jersey (Rahway†); New York (Geneva, Lockport“, Niagara County); Ohio (Columbus); Pennsylvania (State College (Botany Bldg.)); Wisconsin (Sturgeon Bay); Washington DC (Figure 20) ([23,24,27,58]; International Nucleotide Sequence Database Collaboration [°]; Natural History Museum of Denmark Entomology Collection [“]; new record in this publication [†]).

#### 3.4.7. *Drepanaphis keshenae* Granovsky, 1931

**Figure 21:**
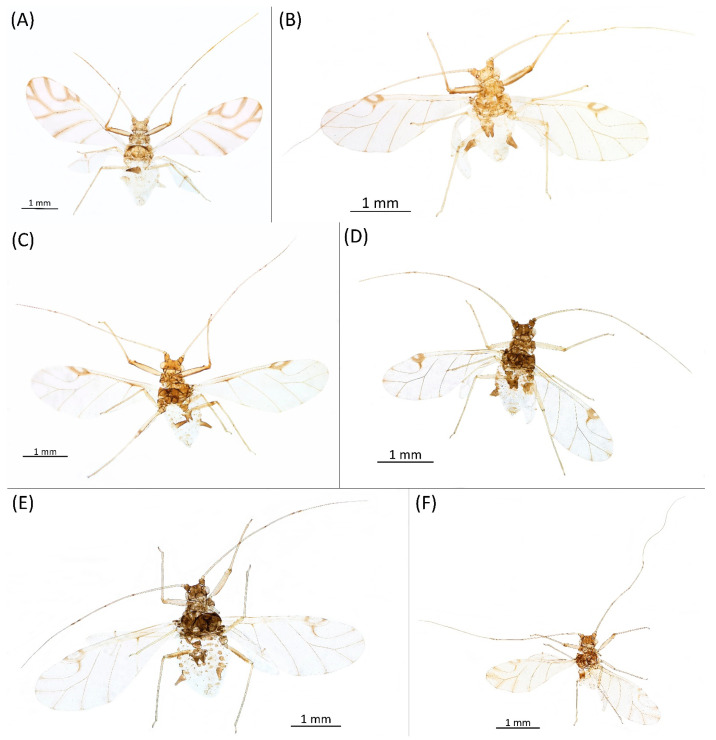
Alate viviparous females of the genus Drepanaphis: (**A**) *D. keshenae*, (**B**) *D. knowltoni*, (**C**) *D. monelli*, (**D**) *D. nigricans*, (**E**) *D. parva*, (**F**) *D. robinsoni* sp. nov.

**Figure 22:**
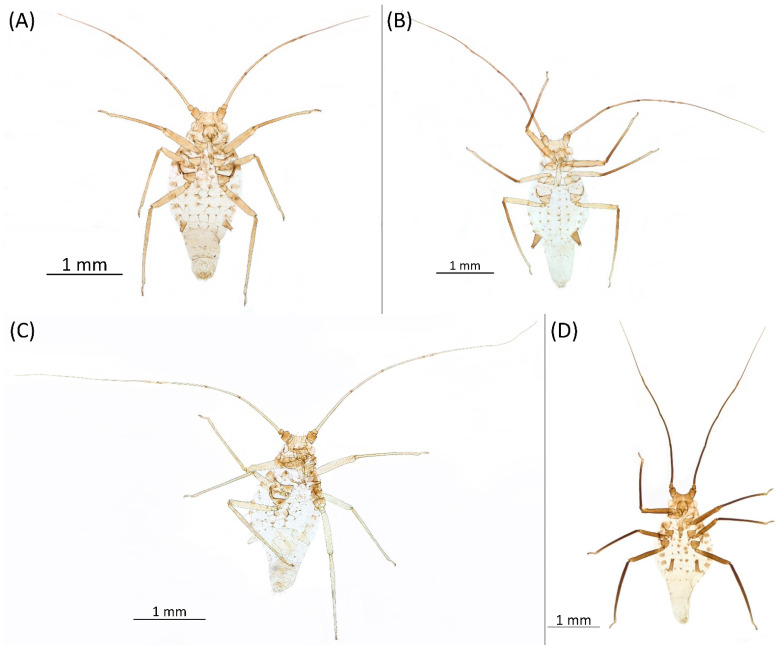
Oviparous females of the genus Drepanaphis: (**A**) *D. keshenae*, (**B**) *D. monelli*, (**C**) *D. nigricans*, (**D**) *D. sabrinae*.

**Figure 23:**
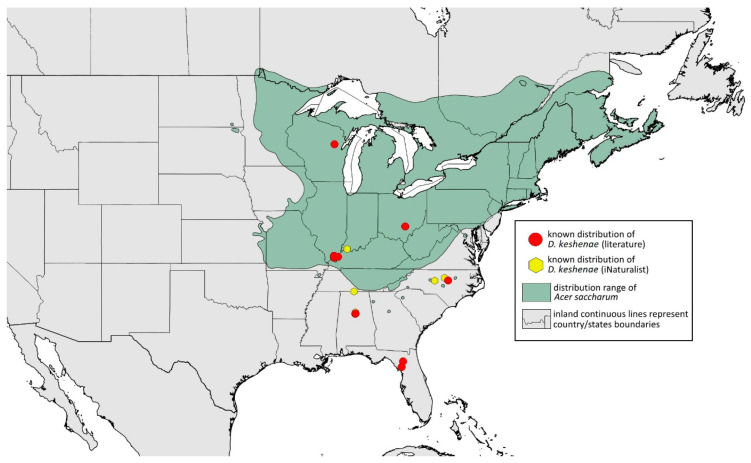
Known distribution of *Drepanaphis keshenae* in North America, with distribution ranges of its host plants.

*Drepanaphis keshenae* Granovsky, 1931: 19: 246, 248 [21]
**Figure 1G, Figure 3G, Figure 4G, Figure 5G, Figure 8C, Figure 11G, Figure 13F, Figure 14F, Figure 21A, Figure 22A and Figure 23; Table 1, Table 2 and Table 3**
  **Material examined:** Lectotype. APHIDIDAE. Drepanaphis keshenae Granovskyi, Det. Granovsky 1929, Sl. 7617, Det. F. C. Hottes, 29, ILL. NAT. HIST. SUR, lectotype//Elizabethtown Ill., VI-20-1929, coll. Frison + Hottes. On *Acer saccharum*. ILL. NAT. HIST. SUR.//INHS, Insect Collection 459,587—three alate viv. fem.
  Additional material examined—Table S6.
**Alate viviparous female—re-description (n = 11)**
  **Colour. In life:** Head and thorax covered with white wax. Antennae pale with dark apices of segments. Eyes red. Abdomen covered with white wax, apart from ABD V with distinct black dorsal tubercles. Fore femora dark; middle and hind femora, tibiae and tarsi pale brown. Wing veins distinctly brown bordered. Siphunculi dark (Figure 8C).
  **Pigmentation of mounted specimens:** Head, thorax, ANT I brown (Figure 21A). ANT II–VI pale brown to brown with darker apices on ANT III–V. Wing veins distinctly brown bordered. Pterostigma distinct, darkly pigmented, with small area inside without pigmentation (Figure 3G). Abdomen pale with brown sclerotisation. DAT dark brown (Figure 1G). Siphunculi pale brown to brown. Cauda, subgenital and anal plate pale. Fore femora darker dorsally (Figure 4G). Middle and hind femora, tibiae and tarsi pale brown to brown. Hind femora with brown smudge.

**Morphometric characters:** Head setae: two pairs of fronto-orbital setae, one pair of postero-dorsal setae, one pair of latero-dorsal setae on dorsal side, 0.02–0.04 mm long with pointed apices; one pair of pointed frontal setae on ventral side 0.07 mm long. ANT/BL 1.5–3.12; PT/BASE 7.2–13.82. ANT III with 10–16 secondary rhinaria, BASE with 4 accessory rhinaria. URS with 6–8 accessory setae. Other ratios: ANT IV/ANT III 0.56–0.79; ANT V/ANT III 0.61–0.81; ANT VI/ANT III 0.99–2.15; URS/ANT III 0.09–0.13; URS/BASE 0.61–0.9; URS/SIPH 0.33–0.64; HT II/ANT III 0.08–0.13; HT II/BASE 0.62–1.00; TIBIA III/BL 0.53–0.82; SIPH/BL 0.096–0.15; SIPH/CAUDA 1.75–3.38. DAT III distinct, 0.24–0.47 mm long (Figure 11G) with pointed setae, 0.02–0.03 mm long at ends. DAT II 0.04 mm long or inconspicuous. DAT I and IV inconspicuous. Dorsal setae 0.02–0.03 mm long, with pointed apices, on ABD I–V on small sclerites. Siphunculi flask-shaped (Figure 5G).
**Oviparous female—description (n = 2)**
  **Colour. In life:** Unknown.
  **Pigmentation of mounted specimens:** Head, thorax, ANT brown. ANT with darker apices on ANT III–V. Abdomen pale with brown sclerotisation. Siphunculi dark brown. Legs brown, tibiae with slightly darker apical parts (Figure 22A).

**Morphometric characters:** Head setae: two pairs of blunt fronto-orbital setae 0.09 mm long, one pair of blunt postero-dorsal setae 0.08 mm long, one pair of blunt latero-dorsal setae 0.05 mm long on dorsal side, one pair of pointed frontal setae 0.08 mm long on ventral side. ANT/BL 1.04–1.15. Other ratios: ANT VI/ANT III 1.78–2.08; PT/BASE 7.09–7.83; SIPH/BL 0.06–0.07; FEMUR III/BL 0.22–0.23; TIBIA III/BL 0.44–0.46; HT II/ANT VI 0.11–0.13; URS/ANT III 0.16–0.24; URS/BASE 0.73–1.1; URS/SIPH 0.48–0.86. ANT III without secondary rhinaria. URS with 6–8 accessory setae. Hind tibiae with 56–67 pseudosensoria distributed along almost their entire lengths. Dorsal setae 0.7–0.11 mm long. Siphunculi tubular, 0.14 mm long.
**Alate male—description (n = 4)**
  **Colour. In life:** Unknown.
  **Pigmentation of mounted specimens:** Head, thorax, ANT I, II, IV, V, VI brown. ANT III pale brown with darker apices of segments. Wing veins brown bordered. Pterostigma distinct, darkly pigmented, with small area inside without pigmentation. Abdomen pale with brown sclerites. Dorsal abdominal tubercles dark brown. Siphunculi, cauda and anal plate brown. Fore femora brown, darker dorsally. Middle and hind femora, tibiae and tarsi brown. Hind femora with brown smudge (Figure 13F).
  **Morphometric characters:** Head setae: two pairs of fronto-orbital setae, one pair of postero-dorsal setae, one pair of latero-dorsal setae on dorsal side, 0.02–0.03 mm long with pointed apices; one pair of pointed frontal setae on ventral side, 0.07 mm long. ANT/BL 1.74–2.2. Other ratios: ANT VI/ANT III 1.22–1.93; PT/BASE 9.2–11.1; SIPH/BL 0.1–0.14; FEMUR III/BL 0.3–0.31; TIBIA III/BL 0.59–0.64; URS/ANT III 0.1–0.17; URS/SIPH 0.42–0.76. ANT III with 71–101 rhinaria, ANT IV with 29–46 rhinaria, ANT V with 25–31 rhinaria. URS with six accessory setae. DAT III 0.08–0.24 mm long with pointed setae 0.02–0.03 mm long at end. Dorsal setae 0.02–0.03 mm long with pointed apices. Siphunculi tubular. Genitalia with basal part of phallus elongated, finger-like (Figure 14F).
  **Remarks:** Smith and Dillery [27] claimed they could not locate any of the slides from Keshena, Wisconsin. Therefore, they suggested Elizabethtown, Illinois, as locus typicus. Additionally, there is a slide no. 95/58 (Biologické centrum AV ČR) from Logan, Utah, dated 16.09.1962, from *Acer grandidentatum*, but it is dark and unverifiable.
  **Host plant:** *Acer saccharum*.
  **Distribution:** USA: Alabama (Aldridge Gardens in Hoover, Old Rocky Ridge (Jefferson County)); Florida (High Springs, Waccasassa River (Levy County)); Illinois (Bell Smith Springs Scenic Area, Dixon Springs, Eddyville (Bell Smith Springs National Natural Landmark), Elizabethtown); North Carolina (Raleigh); Ohio (Hocking County); Wisconsin (Keshena) (Figure 23) [23,27].

Additional distribution from iNaturalist (www.inaturalist.org, accessed on 12 June 2024): Alabama (Birmingham Botanical Gardens, Hoover); Indiana (Evansville); North Carolina (Asheboro, Durham); Tennessee (vicinity of Ardmore).

#### 3.4.8. *Drepanaphis knowltoni* Smith & Dillery, 1968

**Figure 24:**
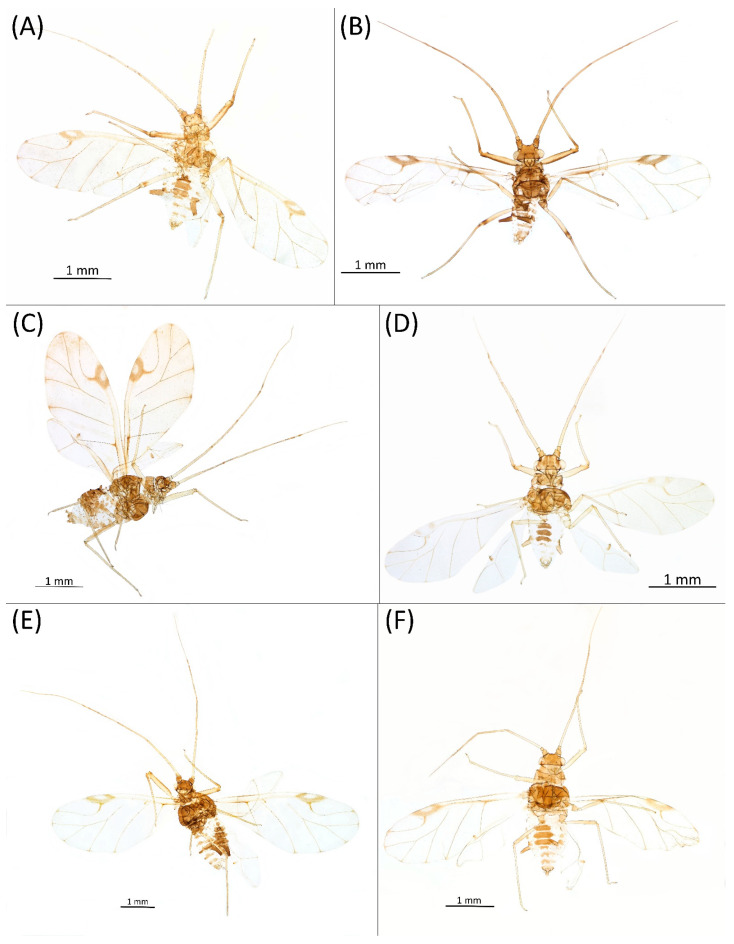
Alate males of the genus Drepanaphis: (**A**) *D. knowltoni*, (**B**) *D. monelli*, (**C**) *D. parva*, (**D**) *D. simpsoni*, (**E**) *D. spicata*, (**F**) *D. utahensis*.

**Figure 25:**
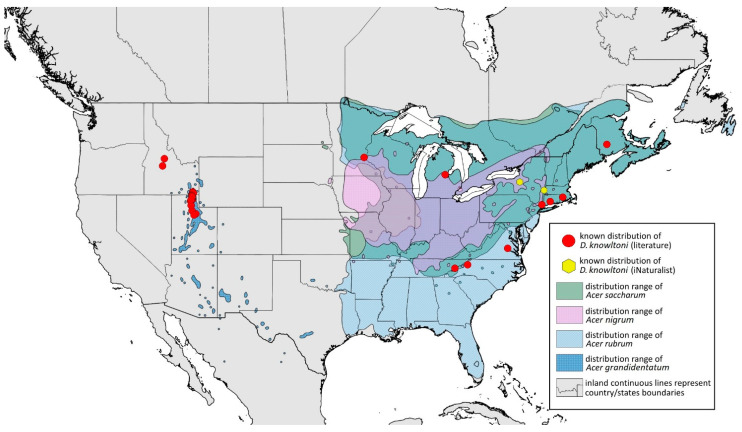
Known distribution of *Drepanaphis knowltoni* in North America, with distribution ranges of its host plants.

*Drepanaphis knowltoni* Smith & Dillery, 1968: 61(1): 186, 195 [27]
**Figure 1H, Figure 3H, Figure 4H, Figure 5H, Figure 11H, Figure 14G, Figure 21B, Figure 24A and Figure 25; Table 1 and Table 3**
  **Material examined:** Holotype. Drepanaphis knowltoni Smith & Dillery Det. Smith & Dillery, 60-886, Acer grandidentanum//Cub River Cany., Ida. 8•15•60, Alate whitish, holotype—K-S—one alate viv. fem. (USNM). Paratype. Drepanaphis knowltoni Smith & Dillery Det. Smith & Dillery, 60-886, Acer grandidentanum//Cub River Cany., Ida. 8•15•60, Alate whitish, holotype—K-S//Museum Paris MNHN 25149—one alate viv. fem.
  Additional material examined—Table S6.
**Alate viviparous female—re-description (n = 24)**
  **Colour. In life:** Body white with wax; may be entirely white, or thoracic lobes and V-shaped area connecting siphunculi with DAT III may be exposed and dark. Front femora, DAT III and siphunculi always dark [27].
  **Pigmentation of mounted specimens:** Head, thorax, ANT I brown (Figure 21B). ANT II–VI pale brown, with darker apices on ANT III–V and dark area with primary rhinarium on ANT VI. Wing veins clear with small area of dark brown pigmentation on end. Pterostigma distinct, darkly pigmented, with small area inside without pigmentation (Figure 3H). Abdomen pale with brown sclerotisation. DAT III (Figure 1H) and siphunculi dark brown. Cauda, subgenital and anal plate pale. Fore femora brown, darker dorsally (Figure 4H). Middle and hind femora, tibiae and tarsi pale brown to brown. Hind femora with brown stripes at margins.
  **Morphometric characters:** Head setae: two pairs of fronto-orbital setae, one pair of postero-dorsal setae, one pair of latero-dorsal setae on dorsal side; one pair of pointed frontal setae on ventral side; 0.02–0.05 mm long with pointed apices. ANT/BL 1.73–2.59; PT/BASE 7.1–12.93. ANT III with 10–15 secondary rhinaria, BASE with 4–5 accessory rhinaria. URS with 6–8 accessory setae. Other ratios: ANT IV/ANT III 0.64–0.79; ANT V/ANT III 0.69–0.85; ANT VI/ANT III 1.23–2.20; URS/ANT III 0.09–0.12; URS/BASE 0.47–0.72; URS/SIPH 0.29–0.42; HT II/ANT III 0.09–0.14; HT II/BASE 0.56–0.85; TIBIA III/BL 0.59–0.82; SIPH/BL 0.12–0.18; SIPH/CAUDA 2.2–3.6. DAT III distinct 0.18–0.28 mm long (Figure 11H), with setae 0.03–0.04 mm long at end. Dorsal setae 0.03–0.04 mm long, with pointed apices. ABD VI with marginal sclerites and 2–5 setae each. Siphunculi flask-shaped (Figure 5H).
  **Oviparous female:** Unknown.
**Alate male—re-description (n = 2)**
  **Colour. In life:** Unknown.
  **Pigmentation of mounted specimens:** Head, thorax, ANT I, II brown. ANT III–VI pale brown, with darker apices on ANT III–V. Wings clear, pterostigma distinct, darkly pigmented, with small area inside without pigmentation. Abdomen pale with brown sclerotisation. Siphunculi, cauda and anal plate brown. Fore femora pale brown, darker dorsally. Middle and hind femora, tibiae, apices of tarsi pale brown. Hind femora with brown stripes at margins. Tibiae with slightly darker apical parts (Figure 24A).

**Morphometric characters:** Head setae: two pairs of fronto-orbital setae, one pair of postero-dorsal setae, one pair of latero-dorsal setae on dorsal side, 0.03–0.04 mm long with pointed apices; one pair of pointed frontal setae on ventral side, 0.06 mm long. ANT/BL 1.64–1.91. Other ratios: ANT VI/ANT III 1.03–1.78; PT/BASE 6.36–11.65; SIPH/BL 0.12; III FEMUR/BL 0.29–0.31; TIBIA III/BL 0.62–0.65; URS/ANT III 0.1; URS/SIPH 0.37. ANT III with 71–79 rhinaria, ANT IV with 29 rhinaria, ANT V with 22 rhinaria. BASE with five accessory rhinaria. URS with eight accessory setae. DAT III 0.1–0.11 mm long with pointed setae 0.04–0.05 mm long at end. Dorsal setae 0.04–0.05 mm long with pointed apices. Spinal sclerites with 2 setae, marginal sclerites with 3–5 setae. Siphunculi tubular. Genitalia with basal part of phallus elongated, robust with capitate apices (Figure 14G).
  **Male:** Unknown.
  **Host plants:** *Acer grandidentatum*, *Acer nigrum*, *Acer rubrum*, *Acer saccharum*.
  **Distribution:** Canada: New Brunswick (Fredericton). USA: Connecticut (Wallingford); Idaho (Cub Creak, Cub River Canyon—locus typicus, Deer Cliff Lodge, Franklin, Mink Creek, Stanley, Strawberry Creek, Thomas Spring); Michigan (Midland); Minnesota (Saint Paul); New York (Mount Kisco); North Carolina (Grandfather Mountain); Rhode Island (Providence); Tennessee (vicinity of Cosby* (0.4 mi up Low Mount Cammerer Trail, Great Smoky Mountains National Park)); Utah (Blacksmith Fork Canyon, Cub River Canyon, Daniel’s Canyon, Logan Canyon, Mantua, Parley’s Canyon, Providence, Provo Canyon, Richmond, Smithfield Canyon, Weber Canyon, Wellsville Canyon); Virginia (Richmond) (Figure 25) [27]; Illinois Natural History Survey Insect Collection [*].

Additional distribution from iNaturalist (www.inaturalist.org, accessed on 12 June 2024): New York (Columbia, Syracuse).

#### 3.4.9. *Drepanaphis monelli* (Davis, 1909)

**Figure 26:**
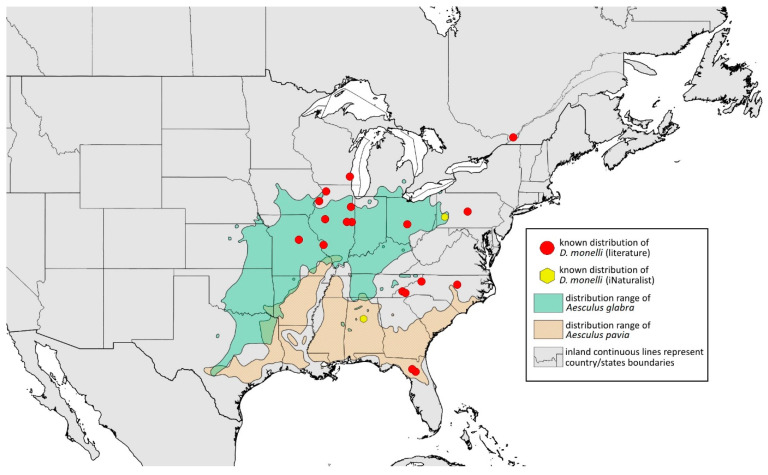
Known distribution of *Drepanaphis monelli* in North America, with distribution ranges of its host plants.

= *Phymatosiphum monelli* Davis, 1909: 2(3): 197 [19]
  *Drepanaphis monelli* Gillette, 1910: 3(4): 371 [20]
  = *Drepanosiphum monelli* Burnham, 1938: 70(9): 184 [60]
**Figure 1I, Figure 3I, Figure 4I, Figure 5I, Figure 10B, Figure 11I; Figure 14H, Figure 21C, Figure 22B, Figure 24B and Figure 26; Table 1, Table 2 and Table 3**
  **Material examined:** Type. Phymatosiphum monelli n.g.e t n.sp., Type, Occ.# 40469. 2n. dt. Zab. Nat. Hist. sz. 3120, John J. Davis.//Phymatosiphum 670 monelli n. sp. -Type- Byckeye, St. Louis, Mo. 30 June’08. J. T. Monell Col. Mounted from alcoholic specimens. John J. Davis.//INHS, Insect Collection 1058879—three alate viv. fem.
  Additional material examined—Table S6.
**Alate viviparous female—re-description (n = 11)**
  **Colour. In life:** Powdery white over entire body, except dark fuscous thoracic lobes. Brownish-yellow, U-shaped line more or less connecting DAT III to siphunculi. Front femora, DAT III and siphunculi dark [27].
  **Pigmentation of mounted specimens:** Head, thorax, ANT I dark brown (Figure 21C). ANT II–VI pale brown with darker apices on ANT III–V. Pterostigma distinct, darkly pigmented, with small area inside without pigmentation (Figure 3I). Abdomen pale with brown sclerotisation. Dorsal abdominal tubercles (Figure 1I) and siphunculi dark brown. Cauda, subgenital and anal plate pale. Fore, middle and hind femora pale brown to brown. Fore femora darker dorsally (Figure 4I); hind femora with brown stripes at margins. Fore tibiae with darker apical part.
  **Morphometric characters:** Head setae: two pairs of fronto-orbital setae, one pair of postero-dorsal setae, one pair of latero-dorsal setae on dorsal side, 0.02–0.03 mm long; one pair of pointed frontal setae on ventral side, 0.06 mm long. ANT/BL 1.9–2.74; PT/BASE 5.51–13.91. ANT III with 9–12 secondary rhinaria, BASE with 4 accessory rhinaria. URS with 6–12 accessory setae (Figure 10B). Other ratios: ANT IV/ANT III 0.66–0.94; ANT V/ANT III 0.72–0.92; ANT VI/ANT III 1.11–2.24; URS/ANT III 0.1–0.13; URS/BASE 0.71–0.86; URS/SIPH 0.34–0.48; HT II/ANT III 0.09–0.13; HT II/BASE 0.62–0.86; TIBIA III/BL 0.55–0.82; SIPH/BL 0.11–0.16; SIPH/CAUDA 1.65–2.78. DAT III distinct, 0.16–0.22 mm long (Figure 11I), with setae 0.02–0.03 mm long at end. ABD I–V with dorsal setae 0.02–0.03 mm long with pointed apices, on small sclerites. Marginal sclerites with 3–5 setae. Siphunculi tubular (Figure 5I).
**Oviparous female—description (n = 5)**
  **Colour. In life:** Unknown.
  **Pigmentation of mounted specimens:** Head, thorax pale brown. ANT brown to dark brown with darker apices on ANT III–V. Abdomen pale with brown sclerotisation. Siphunculi dark brown. Femora and tarsi pale brown. Tibiae dark brown, lighter on ends (Figure 22B).
  **Morphometric characters:** Head setae: two pairs of fronto-orbital setae, one pair of postero-dorsal setae, one pair of latero-dorsal setae on dorsal side, 0.07–0.12 mm long with blunt apices; one pair of pointed frontal setae on ventral side, 0.01 mm long. ANT/BL 1.38–1.83. Other ratios: ANT VI/ANT III 1.64–2.21; PT/BASE 6.29–10.85; SIPH/BL 0.09–0.11; III FEMUR/BL 0.25–0.29; III TIBIAE/BL 0.48–0.55; HT II/ANT VI 0.07–0.1; URS/ANT III 0.14–0.19; URS/BASE 0.75–0.86; URS/SIPH 0.44–0.6. ANT III without secondary rhinaria. URS with 8–10 accessory setae. Hind tibiae with 32–62 pseudosensoria more abundant in middle part of tibiae. Dorsal setae 0.08–0.11 mm long. Siphunculi flask-shaped.
**Alate male—re-description (n = 4)**
  **Colour. In life:** Unknown.
  **Pigmentation of mounted specimens:** Head, thorax, ANT I, II dark brown. ANT III–VI brown with darker apices on ANT III–V. Wings clear with small area of dark brown pigmentation on end. Pterostigma distinct, very darkly pigmented, with small area inside without pigmentation. Abdomen pale with brown sclerotisation. Siphunculi dark brown, cauda and anal plate brown. Fore femora brown, darker dorsally. Middle and hind femora, tibiae and tarsi pale brown. Hind femora with dark brown stripes on ends. Tibiae with slightly darker apical parts (Figure 24B).
  **Morphometric characters:** Head setae: two pairs of fronto-orbital setae, one pair of postero-dorsal setae, one pair of latero-dorsal setae on dorsal side, 0.03–0.05 mm long with pointed apices; one pair of pointed frontal setae on ventral side, 0.06–0.07 mm long. ANT/BL 1.73–2.13. Other ratios: ANT VI/ANT III 1.36–1.66; PT/BASE 7.16–11.92; SIPH/BL 0.11–0.13; FEMUR III/BL 0.29–0.33; TIBIA III/BL 0.58–0.69; URS/ANT III 0.11–0.12; URS/SIHP 0.48–0.79. ANT III with 91–98 rhinaria, ANT IV with 38–45 rhinaria, ANT V with 21–26 rhinaria. URS with eight accessory setae. DAT III distinct, 0.1–0.12 mm long, with pointed setae, 0.03–0.04 mm long at end. Dorsal setae 0.03 mm long on small sclerites. ABD IV–V with two setae on spinal sclerites. Marginal sclerites with 3–4 setae. Siphunculi tubular. Genitalia with basal part of phallus smooth, elongated, hook-shaped (Figure 14H).
  **Host plants:** *Aesculus glabra*, occasionally found on *Aesculus pavia* and *Acer saccharum*.
  **Distribution:** Canada: Quebec (Senneville). USA: Florida (Gainesville, High Springs); Illinois (Havana, Kankakee, Mount Carroll, Oakwood, Rock Island, Urbana); Missouri (Columbia, Saint Louis—locus typicus); North Carolina (Bryson City, Cullowhee, Grandfather Mountain, Raleigh (Umstead Park)); Ohio (Columbus); Pennsylvania (State College); Wisconsin (Milwaukee) (Figure 26) [19,21,23,27,49,58].

Additional distribution from iNaturalist (www.inaturalist.org, accessed on 12 June 2024): Alabama (Birmingham Botanical Gardens); Pennsylvania (Penn Hills).

#### 3.4.10. *Drepanaphis nigricans* Smith, 1941

**Figure 27:**
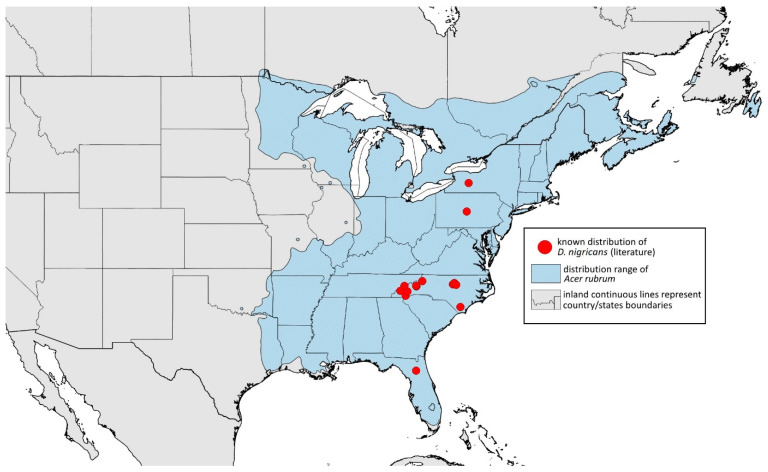
Known distribution of *Drepanaphis nigricans* in North America, with distribution ranges of its host plants.

*Drepanaphis nigricans* Smith, 1941: 57(2): 228, 236 [23]
**Figure 1J, Figure 3J, Figure 4J, Figure 5J, Figure 11J, Figure 21D, Figure 22C and Figure 27; Table 2 and Table 4**
  **Material examined:** Holotype. Drepanaphis nigricans Smith Holotype Type No 55835. D.D.N.N.M.//N.C. 41-151, Acer rubrum, Busick, N.C., (Park Way), 2 July 1941, CF. Smith—five alate viv. fem. (USNM). Paratype. Drepanaphis nigricans C. F. Smith//N. C. Aphids, Host Acer rubrum, Blowing Roak, N. C., Date June 12 1940, C. F. Smith, Black light spots//INHS, Insect Collection 1058449—three alate viv. fem. Paracotype. Drepanaphis nigricans C. F. Smith//N. C. 41-151, Acer rubrum, Busick, NC., Park Way, July 2, 1941, CF Smith//Museum Paris MNHN 25153—six alate viv. fem.
  Additional material examined—Table S6.
**Alate viviparous female—re-description (n = 51)**
  **Colour. In life:** Black with pale legs, wings with noticeable dark spots at ends of veins. Head and pronotum with three longitudinal white wax stripes, median pronotal stripe interrupted, often faint. Mesonotum with two transverse rows of four small wax dots anteriorly (medial pairs sometimes missing) and one pair of elongated dots posteriorly. Metanotum with two wax dots laterally. Abdomen with many wax dots, most dense at posterior end [27].
  **Pigmentation of mounted specimens:** Head, thorax, ANT I dark brown (Figure 21D). ANT II–VI pale brown with darker apices on ANT III–V. Wings clear with dark pigmentation at end of veins. Pterostigma distinct, darkly pigmented, oval with small area inside without pigmentation (Figure 3J). Abdomen pale with brown dorsal sclerotisation. Dorsal abdominal tubercles (Figure 1J) and siphunculi dark brown. Cauda, subgenital and anal plate brown. Fore femora pale brown (Figure 4J).
  **Morphometric characters:** Head setae: two pairs of fronto-orbital setae, one pair of postero-dorsal setae, one pair of latero-dorsal setae on dorsal side, 0.01–0.02 mm long; one pair of pointed frontal setae on ventral side, 0.06–0.07 mm long. ANT/BL 2–3.38; PT/BASE 7.5–15.8. ANT III with 11–20 secondary rhinaria, BASE with 4 accessory rhinaria. URS with 6–10 accessory setae. Other ratios: ANT IV/ANT III 0.63–0.79; ANT V/ANT III 0.64–0.84; ANT VI/ANT III 1.18–2.26; URS/ANT III 0.09–0.15; URS/BASE 0.6–0.94; URS/SIPH 0.45–0.7; HT II/ANT III 0.09–0.13; HT II/BASE 0.6–1.02; TIBIA III/BL 0.55–0.86; SIPH/BL 0.077–0.14; SIPH/CAUDA 1.03–2.63. DAT I inconspicuous. DAT II 0.02–0.04 mm long, DAT III biggest 0.14–0.25 mm long (Figure 11J), DAT IV 0.2–0.6 mm long. Ends of tubercles with very short setae, about 0.01 mm long. ABD I–V with dorsal setae 0.01–0.02 mm long, on small sclerites. Marginal sclerites with 3–6 setae. Siphunculi flask-shaped (Figure 5J).
**Oviparous female—description (n = 1)**
  **Colour. In life:** Unknown.
  **Pigmentation of mounted specimens:** Head and thorax pale brown. ANT I–II brown; ANT III–VI pale brown. ANT II–VI with darker apices on ANT III–V and dark area with primary rhinarium on ANT VI. Siphunculi, cauda, subgenital and anal plate pale brown. Legs pale (Figure 22C).
  **Morphometric characters:** Head setae: two pairs of fronto-orbital setae 0.08–0.1 mm long, one pair of postero-dorsal setae 0.08 mm long, one pair of latero-dorsal setae on dorsal side 0.04–0.05 mm long with blunt apices; one pair of pointed frontal setae on ventral side, 0.09–0.1 mm long. ANT/BL 1.69–1.71. Other ratios: ANT VI/ANT III 2.29–2.45; PT/BASE 12.38–12.72; SIPH/BL 0.08–0.09; FEMUR III/BL 0.26–0.28; TIBIA III/BL 0.51–0.52; HT II/ANT VI 0.06; URS/ANT III 0.14; URS/BASE 0.77; URS/SIPH 0.53. ANT III with 3–4 secondary rhinaria. URS with 12 accessory setae. Hind tibiae with 53–62 pseudosensoria more abundant in middle part of tibiae, closer to distal part of femur. Dorsal setae 0.07–0.09 mm long, on ABD VIII slightly shorter. Siphunculi flask-shaped.
  **Male:** Unknown.
  **Host plant:** *Acer rubrum*.
  **Distribution:** USA: Florida (Gainesville); New York (Sparta); North Carolina (Blowing Rock, Bolton, Busick—locus typicus, Cashiers, Chapel Hill, Durham, Great Smoky Mountains National Park* (Goldmine Loop Trail), Mount Mitchell, Raleigh (Umstead Park), Sunburst); Pennsylvania (State College); Tennessee (Great Smoky Mountains National Park* (Low Mount Cammerer Trail)) (Figure 27) ([23,27]; Illinois Natural History Survey Insect Collection [*]).

#### 3.4.11. *Drepanaphis parva* Smith, 1941

**Figure 28:**
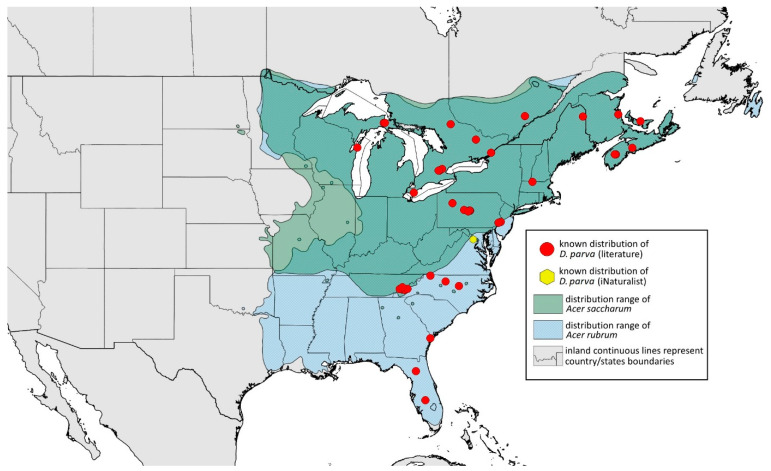
Known distribution of *Drepanaphis parva* in North America, with distribution ranges of its host plants.

*Drepanaphis parva* Smith, 1941: 57(2): 228, 237 [23]
  = *Drepanaphis rubrum* Smith, 1941: 57(2): 228, 238 [23]
  = *Drepanaphis parvus* Smith & Knowlton, 1943: 59(2): 173 [24]
**Figure 1K, Figure 3K, Figure 4K, Figure 5K, Figure 7C, Figure 11K, Figure 14I, Figure 21E, Figure 24C and Figure 28; Table 3 and Table 4**
  **Material examined:** Holotype. Drepanaphis parvus Smith Holotype Type No 55836. D.D.N.N.M.//N. C. Aphids, Host Acer, Greensboro, N. C. 193, Date 5-3-40, C. F. Smith—three alate viv. fem. (USNM). Paratype. Drepanaphis parvus C. F. Smith//N. C. Aphids, Host Acer rubrum, Raleigh, NC, Date May 12 1941, C. F. Smith//INHS, Insect Collection 1058907—six alate viv. fem. Paracotype. Drepanaphis parvus C. F. Smith//N. C. Aphids, Host Acer, Greensboro, N. C. 193, Date 5-3-40, C. F. Smith//INHS, Insect Collection 1058906—six alate viv. fem. Paracotype. Drepanaphis parvus C. F. Smith//N. C. Aphids, Host Acer, Greensboro, N. C. 193, Date 5-3-40, C. F. Smith//Museum Paris MNHN 22493—six alate viv. fem.
  Additional material examined—Table S6.
**Alate viviparous female—re-description (n = 15)**
  **Colour. In life:** Head and thorax reddish brown; head and pronotum with five longitudinal white wax stripes; pronotal wax pattern as in *D. acerifoliae*. Abdomen green–grey–brown with white tip, white wax dots in longitudinal rows in line with those on thorax [27].
  **Pigmentation of mounted specimens:** Head, ANT I, thorax brown to dark brown (Figure 21E). ANT II–VI pale brown with darker apices on ANT III–V and dark area with primary rhinarium on ANT VI. Wing veins diffusely bordered. Pterostigma distinct, darkly pigmented, with small area inside without pigmentation (Figure 3K and Figure 7C). Abdomen pale with DAT I–IV (Figure 1K) and sclerotisation dark. Siphunculi brown, cauda, subgenital and anal plate pale brown. Fore femora pale brown (Figure 4K). Hind femora with darker smudge.
  **Morphometric characters:** Head setae: two pairs of fronto-orbital setae, one pair of postero-dorsal setae, one pair of latero-dorsal setae on dorsal side, 0.02–0.03 mm long with blunt apices; one pair of pointed frontal setae on ventral side, 0.05–0.06 mm long. ANT/BL 1.41–1.94; PT/BASE 6.36–11.31. ANT III with 9–15 secondary rhinaria, BASE with 4 accessory rhinaria. URS with 4–8 accessory setae. Other ratios: ANT IV/ANT III 0.55–0.79; ANT V/ANT III 0.52–1.16; ANT VI/ANT III 1.05–1.7; URS/ANT III 0.08–0.11; URS/BASE 0.67–0.77; URS/SIPH 0.35–0.43; HT II/ANT III 0.09–0.14; HT II/BASE 0.67–1.08; TIBIA III/BL 0.46–0.59; SIPH/BL 0.08–0.13; SIPH/CAUDA 1.61–2.46. DAT I 0.07–0.11 mm long; DAT II 0.05–0.08 mm long; DAT III biggest, 0.14–0.19 (Figure 11K); DAT IV smallest, 0.04–0.07 mm long. Dorsal setae 0.01–0.03 mm with blunt apices, on small sclerites. ABD VII–VIII with setae pointed and slightly longer, 0.03–0.05 mm long. Siphunculi flask-shaped (Figure 5K).
  **Oviparous female:** Unknown.
**Alate male—re-description (n = 1)**
  **Colour. In life:** Unknown.
  **Pigmentation of mounted specimens:** Head, ANT I, thorax brown. ANT II–VI pale brown. ANT III–V with slightly darker apices on ends and dark area with primary rhinarium on ANT VI. Wing veins diffusely bordered. Pterostigma distinct, darkly pigmented, with small area inside without pigmentation. Abdomen pale with dark tubercles and sclerotisation. Cauda and anal plate brown. Legs pale; hind femora with brown smudge (Figure 24C).
  **Morphometric characters:** Head setae: two pairs of fronto-orbital setae, one pair of postero-dorsal setae, one pair of latero-dorsal setae on dorsal side, 0.03–0.05 mm long with pointed apices; one pair of pointed frontal setae on ventral side, 0.05–0.06 mm long. ANT/BL 1.83–1.93. Other ratios: ANT VI/ANT III 1.47–1.71; PT/BASE 11.69–13.46; SIPH/BL 0.08–0.09; FEMUR III/BL 0.29–0.3; TIBIA III/BL 0.61; URS/ANT III 0.11; URS/SIPH 0.69. ANT III with 91–97 rhinaria, ANT IV with 38–41 rhinaria, ANT V with 21–26 rhinaria. URS with eight accessory setae. DAT I–II inconspicuous. DAT III 0.13–0.15 mm long, DAT IV 0.04–0.05 mm long. Dorsal setae 0.04–0.06 mm long, with pointed apices. ABD I–V with 2–4 setae on spinal sclerites. Marginal sclerites with 2–6 setae. Genitalia with basal part of phallus robust, elongated, hook-shaped (Figure 14I).
  **Host plants:** *Acer rubrum*, *Acer saccharum*.
  **Distribution:** Canada: New Brunswick (Middle Kouchibouguac‡); Nova Scotia (Jakes Landing^, Kejimkujik Main Parkway‡, Upper Hammonds Plains‡); Ontario (Callander, Cambridge°, Corwhin^, Front of Yonge‡ (44°30′00.0″ N 75°54′00.0″ W), Griffith^, Kitchener‡, Marentette Beach in Wheatley^); Prince Edward Island (Stanhope); Quebec (Shawinigan (Lac Wapizagonke‡)). USA: Florida (Gainesville, Sebring (Highlands Hammock State)); Georgia (Savannah); Maine (Presque Isle); Massachusetts (Mount Grace (Warwick)); North Carolina (Andrew’s Bald in Great Smoky Mountains National Park* (35°32′16.8″ N 83°29′38.4″ W), Blue Ridge Parkway, Greensboro—locus typicus, Purchase Knob in Great Smoky Mountains National Park* (35°35′16.2″ N 83°03′53.4″ W), Raleigh, Sparta‴); Michigan (Chippewa Township° (46°18′00.0″ N 85°06′00.0″ W), Eckerman Corner^); Pennsylvania (Bethayres, Center Hall, Cooksburg, Miquon, Philipsburg, Pleasant Gap, State College); Tennessee (Bote Mountain Trail in Great Smoky Mountains National Park* (35°34′37.1″ N 83°44′02.3″ W), Gatlinburg°); Wisconsin (Sturgeon Bay) (Figure 28) ([23,24,27]; Centre for Biodiversity Genomics—Canadian Specimens [‡]; Illinois Natural History Survey Insect Collection [*]; International Barcode of Life project (iBOL) [^]; International Nucleotide Sequence Database Collaboration [°]; NMNH Extant Specimen Records (USNM, US) [‴]).

Additional distribution from iNaturalist (www.inaturalist.org, accessed on 12 June 2024): Pennsylvania (Wyndmoor); Virginia (Dulles).

#### 3.4.12. *Drepanaphis robinsoni* Malik sp. nov.

**Figure 29:**
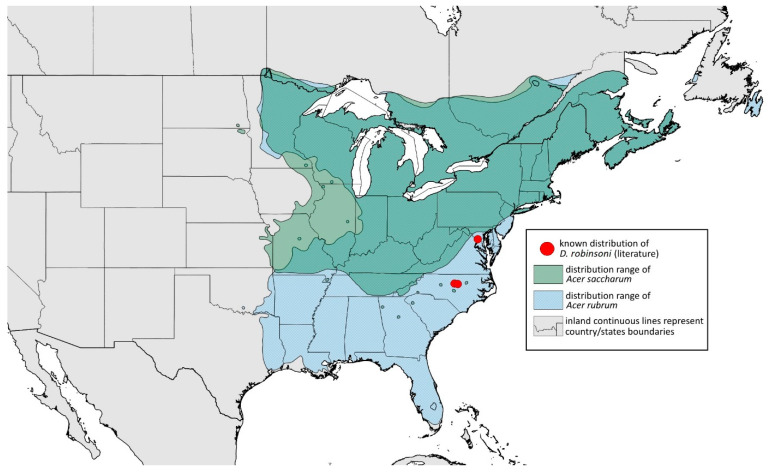
Known distribution of *Drepanaphis robinsoni* in North America, with distribution ranges of its host plants.

urn:lsid:zoobank.org:act:3C2B4AAD-E06A-410B-940D-AEA4E96662FC
**Figure 1L, Figure 3L, Figure 4L, Figure 5L, Figure 11L, Figure 21F and Figure 29; Table 4**
  **Material examined:** Holotype. *Drepanaphis choanotricha* Dillery & Smith [MS], Al. Viv ♀, US/153 HLGS det.//Acer saccharum, Umstead Park, Raleigh N.C., U.S.A, 19•VI•1966, HLGS leg. BM 1982-492//NHMUK 14314713—one alate viv. fem. PARATYPES *Drepanaphis choanotricha* Dillery & Smith [MS], Al. Viv ♀, US/153 HLGS det.//Acer saccharum, Umstead Park, Raleigh N.C., U.S.A, 19•VI•1966, HLGS leg. BM 1982-492//NHMUK 14314714—one alate viv. fem. *Drepanaphis parva* Smith, BM 1984-340 Det: Dillery & Smith//N.U.S.A, Acer rubrum, Chapel Hill N.C., 2•VII•1965, Dillery and Smith leg. 65.127//NHMUK 12821447—three alate viv. fem. *Drepanaphis parva* Smith, BM 1984-340 Det.: C. F. Smith//N.U.S.A, Acer rubrum, Raleigh N.C., 5•VII•1959, Leg: C. F. Smith. 59.394 O//NHMUK 12821448—three alate viv. fem; SA02-385-05-001 DZUS—one alate viv. fem., 22 September 2022, Washington, D.C., USA, Acer rubrum, K. Malik leg. & det.
  Additional material examined—Table S6.
**Alate viviparous female—description (n = 11)**
  **Colour. In life:** Unknown.
  **Pigmentation of mounted specimens:** Head, ANT I, thorax brown (Figure 21F). ANT II–V pale brown with darker apices on ANT III–V and dark area with primary rhinarium on ANT VI. Wings clear with smudge-bordered ends of veins. Pterostigma distinct, darkly pigmented, with small area inside without pigmentation (Figure 3L). Abdomen pale with DAT I pale, DAT II and IV brown and DAT III dark brown (Figure 1L). Siphunculi, cauda, subgenital and anal plate pale. Legs pale brown, fore femora slightly darker at margins (Figure 4L).
  **Morphometric characters:** Head setae: two pairs of fronto-orbital setae, one pair of postero-dorsal setae, one pair of latero-dorsal setae on dorsal side, 0.01–0.02 mm long with blunt apices; one pair of pointed frontal setae on ventral side, 0.05–0.06 mm long. ANT/BL 2–3.2; PT/BASE 9.39–15.7. ANT III with 9–13 secondary rhinaria, BASE with 4 accessory rhinaria. URS with six accessory setae. Other ratios: ANT IV/ANT III 0.66–0.81; ANT V/ANT III 0.69–0.85; ANT VI/ANT III 1.54–2.67; URS/ANT III 0.09–0.12; URS/BASE 0.67–0.84; URS/SIPH 0.44–0.62; HT II/ANT III 0.09–0.12; HT II/BASE 0.64–0.86; TIBIA III/BL 0.54–0.85; SIPH/BL 0.09–0.17; SIPH/CAUDA 1.63–2.12. DAT I 0.06–0.08 mm long; DAT II 0.05–0.11 mm long; DAT III biggest, 0.14–0.22 mm long (Figure 11L); DAT IV smallest, 0.04–0.06 mm long. Dorsal setae 0.02–0.03 mm long with blunt apices. Marginal sclerites with 3–4 setae. Siphunculi flask-shaped (Figure 5L).
  **Oviparous female:** Unknown.
  **Male:** Unknown.
  **Remarks:** The new species *D. robinsoni* was designated based on two other incorrectly designated species of the *Drepanaphis* genus. These species include *D. choanotricha* (NHMUK 14314713, NHMUK 14314714) and *D. parva* (NHMUK 12821445, NHMUK 12821447, INHS Insect Collection 1058904). The fresh material of this new species was also collected in Washington, D.C.
  **Diagnosis:** Alate viviparous females of the new species can be easily distinguished from the closely related *D. parva* by their pale siphunculi and lack of sclerotisation on the abdomen and from *D. choanotricha* by the presence of only four accessory rhinaria.
  **Etymology:** We are pleased to name this new species in honour of Arthur Grant Robinson, an eminent Canadian aphidologist, who collected specimens of this species.
  **Host plants:** *Acer rubrum*, *Acer saccharum*.
  **Distribution:** USA: North Carolina (Chapel Hill, Doughton, Raleigh—locus typicus (Umstead Park)); Washington, D.C. (Figure 29).

**Table 4:** Measurements (in mm) of alate viviparous females of *Drepanaphis* (part 2).

Character	*D. nigricans* n = 51	*D. parva* n = 15	*D. robinsoni* sp. nov. n = 11	*D. sabrinae* n = 14	*D. saccharini* n = 15	*D. simpsoni* n = 12	*D. spicata* n = 19	*D. tissoti* n = 25	*D. utahensis* n = 26
BL	1.04–2.12	1.85–2.7	1.22–1.73	1.75–2.76	1.58–2.40	1.69–2.41	2.12–3	0.99–1.8	1.55–2.8
HW	0.2–0.33	0.29–0.39	0.25–0.29	0.33–0.39	0.29–0.33	0.3–0.36	0.3–0.43	0.21–0.29	0.27–0.39
ANT I-VI	3.29–5.16	3.22–4.9	3.15–3.91	3.2–5	3.65–4.57	2.62–3.43	4.7–5.86	3.11–4.19	2.32–4.17
ANT III	0.6–1.04	0.81–1.31	0.73–0.88	0.77–1.19	0.80–1.1	0.69–0.85	1.04–1.37	0.7–1.11	0.74–1
ANT IV	0.52–0.73	0.52–0.87	0.52–0.67	0.59–0.99	0.58–0.89	0.51–0.61	0.78–1.07	0.46–0.63	0.48–0.78
ANT V	0.56–0.78	0.51–0.84	0.57–0.72	0.61–0.97	0.58–0.85	0.44–0.58	0.76–1.04	0.47–0.71	0.44–0.73
ANT VI	1.02–1.99	0.95–1.85	1.51–1.95	1.04–1.85	1.16–1.68	0.73–0.97	1.54–2.33	1.03–2.11	0.76–1.5
BASE	0.1–0.16	0.12–0.16	0.1–0.14	0.13–0.2	0.1–0.14	0.12–0.15	0.12–0.19	0.09–0.13	0.12–0.17
PT	0.9–1.84	0.83–1.7	1.03–1.83	0.9–1.66	1.04–1.56	0.6–0.85	1.37–2.17	0.9–1.98	0.62–1.33
FEMUR I length	0.45–0.72	0.53–0.66	0.45–0.55	0.6–0.88	0.53–0.79	0.5–0.59	0.74–1.03	0.44–0.6	0.51–0.87
FEMUR I width	0.07–0.13	0.08–0.15	0.09–0.12	0.11–0.18	0.11–0.15	0.14–0.17	0.09–0.18	0.05–0.09	0.1–0.18
FEMUR III	0.41–0.65	0.4–0.67	0.39–0.47	0.51–0.83	0.46–0.7	0.49–0.65	0.65–0.9	0.4–0.59	0.5–0.73
TIBIA III	0.84–1.34	1.03–1.45	0.9–1.05	1.11–1.61	1.03–1.51	0.92–1.04	1.36–1.89	0.82–1.16	1.05–1.48
HT II	0.08–0.12	0.1–0.13	0.08–0.09	0.10–0.13	0.10–0.12	0.10–0.12	0.11–0.14	0.08–0.1	0.1–0.14
URS	0.08–1	0.09–0.11	0.08–0.09	0.12–0.13	0.09–0.11	0.08–0.09	0.1–0.12	0.08–0.1	0.08–0.1
SIPH	0.12–0.22	0.21–0.3	0.14–0.21	0.19–0.34	0.19–0.21	0.16–0.21	0.24–0.43	0.13–0.21	0.18–0.3
CAUDA	0.07–0.96	0.09–0.15	0.08–0.1	0.09–0.13	0.08–0.14	0.1–0.13	0.1–0.15	0.07–0.11	0.1–0.17

#### 3.4.13. *Drepanaphis sabrinae* Miller, 1937

**Figure 30:**
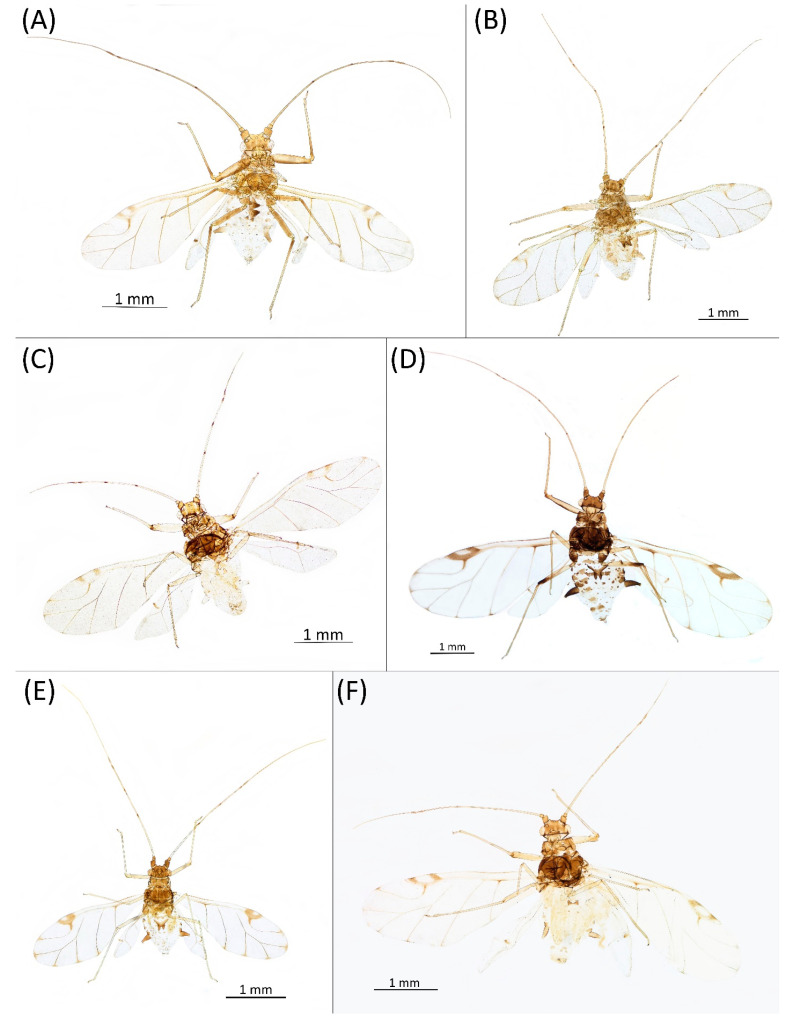
Alate viviparous females of the genus *Drepanaphis*: (**A**) *D. sabrinae*, (**B**) *D. saccharini*, (**C**) *D. simpsoni*, (**D**) *D. spicata*, (**E**) *D. tissoti*, (**F**) *D. utahensis*.

**Figure 31:**
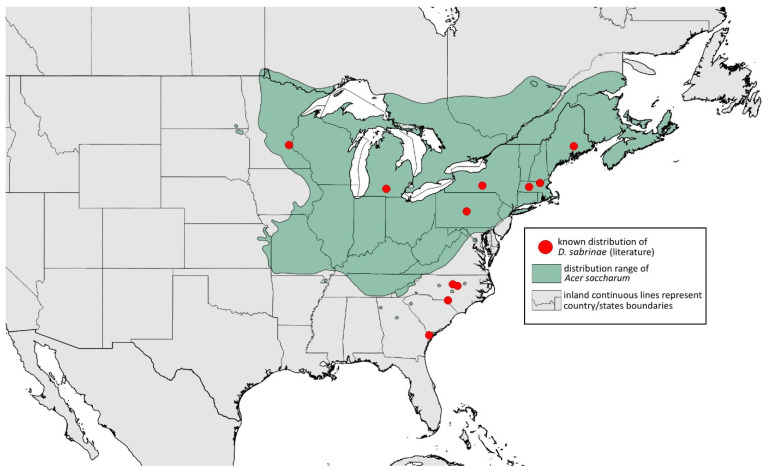
Known distribution of *Drepanaphis sabrinae* in North America, with distribution ranges of its host plants.

*Drepanaphis sabrinae* Miller, 1937: 69(5): 111 [22]
**Figure 1M, Figure 2C, Figure 3M, Figure 4M, Figure 5M, Figure 11M, Figure 22D, Figure 30A and Figure 31; Table 2 and Table 4**
  **Material examined:** See Table S6.
**Alate viviparous female—re-description (n = 14)**
  **Colour. In life:** Abdomen orange brown, sometimes yellowish. Small flecks of white wax present, three to five on head; four antero-medial, two antero-lateral and two postero-medial on mesonotum; two lateral on metanotum; some on abdomen. Pronotum with three longitudinal wax stripes, median stripe interrupted and faint [27].
  **Pigmentation of mounted specimens:** Head, ANT, thorax and legs brown to dark brown (Figure 30A). ANT II–VI with darker apices on ANT III–V and dark area with primary rhinarium on ANT VI. Wings clear, pterostigma distinct, darker pigmentation on ends, with small area inside without pigmentation (Figure 3M). Abdomen pale with dark dorsal tubercles, lighter on bases, darker on tips (Figure 1M), and pale brown sclerotisation. Siphunculi pale brown to brown, slightly darker on ends (Figure 5M). Fore femora dark dorsally (Figure 4M). Tibiae with slightly darker knee apical parts on ends. Cauda, subgenital and anal plate pale.

**Morphometric characters:** Head setae: two pairs of fronto-orbital setae, one pair of postero-dorsal setae, one pair of latero-dorsal setae on dorsal side, 0.02–0.04 mm long with pointed apices; one pair of pointed frontal setae on ventral side, 0.07–0.01 mm long. ANT/BL 1.18–2.51; PT/BASE 5.82–9.66. ANT III with 6–10 secondary rhinaria, BASE with 5–6 accessory rhinaria (Figure 2C). URS with 8–14 accessory setae. Other ratios: ANT IV/ANT III 0.65–0.88; ANT V/ANT III 0.67–0.93; ANT VI/ANT III 1.25–1.9; URS/ANT III 0.11–0.14; URS/BASE 0.68–0.95; URS/SIPH 0.42–0.65; HT II/ANT III 0.09–0.14; HT II/BASE 0.53–0.77; TIBIA III/BL 0.52–0.78; SIPH/BL 0.09–0.16; SIPH/CAUDA 2.01–2.54. DAT I 0.09–0.15 mm long; DAT II and III equal, 0.12–0.23 mm long (Figure 11M); DAT IV smallest, 0.03–0.07 mm long. Dorsal setae 0.03–0.04 mm long with pointed apices, on small sclerites. Marginal sclerites with 2–5 setae. Siphunculi tubular (Figure 5M).
**Oviparous female—description (n = 2)**
  **Colour. In life:** Unknown.
  **Pigmentation of mounted specimens:** Head and thorax brown, abdomen pale. ANT I–II brown. ANT III–VI dark brown with lighter apices on ANT VI. Abdomen pale with dark sclerotisation. Siphunculi, subgenital, anal plate and cauda brown. Fore, middle, hind femora and siphunculi brown. Tibiae dark brown. Tarsi brown (Figure 22D).
  **Morphometric characters:** Head setae: two pairs of fronto-orbital setae 0.08–0.09 mm long, one pair of postero-dorsal setae mm long, one pair of latero-dorsal setae 0.05–0.7 mm long with pointed apices on dorsal side; one pair of pointed frontal setae on ventral side 0.09 mm long. ANT/BL 1.33–1.39. Other ratios: ANT VI/ANT III 1.5–1.76; PT/BASE 7.19–7.57; SIPH/BL 0.08–0.1; FEMUR III/BL 0.24; TIBIA III/BL 0.46–0.47; HT II/ANT VI 0.09; URS/ANT III 0.15–0.16; URS/BASE 0.75–0.86; URS/SIPH 0.5–0.6. ANT III with one or without secondary rhinaria. URS with 8–10 accessory setae. Hind tibiae with 70–76 pseudosensoria distributed along almost their entire lengths. Dorsal setae 0.04 -0.09 mm long. Siphunculi tubular.
  **Male:** Unknown.
  **Remarks:** Smith [23] lists Hardeeville as a place of occurrence, along with Raleigh and Chapel Hill, and located all of them in North Carolina. However, the locality of Hardeeville occurs only in South Carolina and is thus classified in this work as such a locality for this species.
  **Host plant:** *Acer saccharum*.
  **Distribution:** USA: Maine (Orono); Massachusetts (Amherst—locus typicus, Groton); Michigan (Albion (College Campus)); Minnesota (Saint Paul); New York (Ithaca); North Carolina (Chapel Hill, Laurinburg (Jaycee Park†), Raleigh (Fred Fletcher Park)); Pennsylvania (State College); South Carolina (Hardeeville) (Figure 31) ([23,27]; new record in this publication [†]).

#### 3.4.14. *Drepanaphis saccharini* Smith & Dillery, 1968

**Figure 32:**
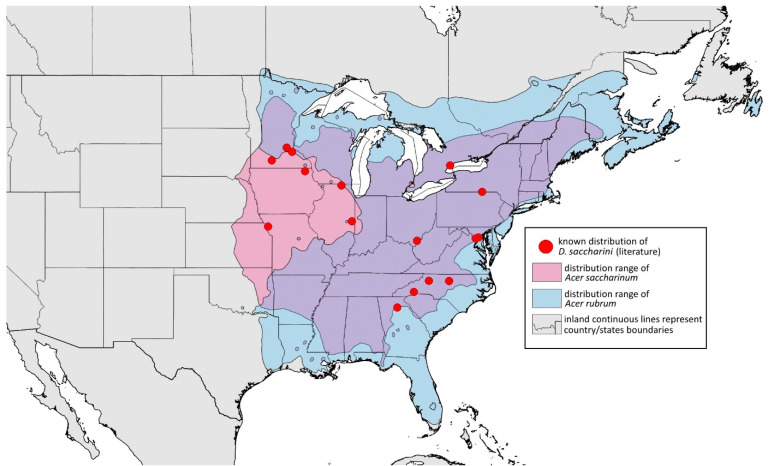
Known distribution of *Drepanaphis saccharini* in North America, with distribution ranges of its host plants.

*Drepanaphis saccharini* Smith & Dillery, 1968: 61(1): 186, 198 [27]
**Figure 1N, Figure 3N, Figure 4N, Figure 5N, Figure 30B and Figure 32; Table 2**
  **Material examined:** Holotype. Drepanaphis saccharini S & D Det. Smith & Dillery, 60-887, Silver Maple//St. James, Minn, 8•3•60, Holotype, S•S—one alate viv. fem. (USNM). Paratype. Drepanaphis saccharini S & D Det. Smith & Dillery, 60-887, Silver Maple, Paratype//St. James, Minn, 8•3•60, S•S//INHS, Insect Collection 1058912—one alate viv. fem. Paratype. Drepanaphis saccharini Smith & Dillery, paratype, Det: Smith & Dillery, BM 1984-340//N.U.S.A., Pl. Silver maple, Loc. St. James, Minn., Date 3.VIII.1960, Leg. S. S. S//NHMUK 12821470—three alate viv. fem.
  Additional material examined—Table S6.
**Alate viviparous female—re-description (n = 15)**
  **Colour. In life:** Abdomen greenish with white tip [27].
  **Pigmentation of mounted specimens:** Head, ANT I and thorax brown (Figure 30B). ANT II–VI pale brown with darker apices on ANT III–V and dark area with primary rhinarium on ANT VI. Wings clear, pterostigma palely pigmented, with large area inside without pigmentation (Figure 3N). Abdomen pale with dorsal abdominal tubercles dark (Figure 1N) and sclerotisation pale brown. Siphunculi, cauda, subgenital and anal plate pale brown. Legs pale brown. Fore femora slightly darker on margins (Figure 4N), hind femora with smudge.
  **Morphometric characters:** Head setae: two pairs of fronto-orbital setae, one pair of postero-dorsal setae, one pair of latero-dorsal setae on dorsal side, 0.02–0.05 mm long with pointed apices; one pair of pointed frontal setae on ventral side, 0.08 mm long. ANT/BL 1.2–2.4; PT/BASE 8.68–14.04. ANT III with 6–10 secondary rhinaria, BASE with 4 accessory rhinaria. URS with 6–8 accessory setae. Other ratios: ANT IV/ANT III 0.69–0.87; ANT V/ANT III 0.69–0.83; ANT VI/ANT III 1.27–1.97; URS/ANT III 0.11–0.13, URS/BASE 0.78–0.91; URS/SIPH 0.46–0.57; HT II/ANT III 0.1–0.14; HT II/BASE 0.8–1.1; TIBIA III/BL 0.56–0.66. SIPH/BL 0.09–0.12; SIPH/CAUDA 1.48–2.34. DAT I and II equal 0.03–0.08 mm long; DAT III biggest, 0.08–0.17 mm long (Figure 11N); DAT IV smallest, 0.02–0.04 mm long. ABD I–V with dorsal setae 0.03–0.04 mm long with pointed apices, on small sclerites. Marginal sclerites with 2–6 setae. Siphunculi flask-shaped (Figure 5N).
  **Remarks:** Sexual generation remains unknown.
  **Host plants:** *Acer saccharinum*, *Acer rubrum*.
  **Distribution:** Canada: Ontario (Toronto). USA: Georgia (Oakwood); Illinois (Chemung, Oakwood); Iowa (Decorah); Kansas (Wathena); Maryland (Beltsville, Plummers Island); Minnesota (Cannon Falls, Saint James—locus typicus, Savage); New York (Chemung); North Carolina (Burlington, Hendersonville, Moravian Falls); Ohio (Gallipolis) (Figure 32) [27].

#### 3.4.15. *Drepanaphis simpsoni* Smith, 1959

**Figure 33:**
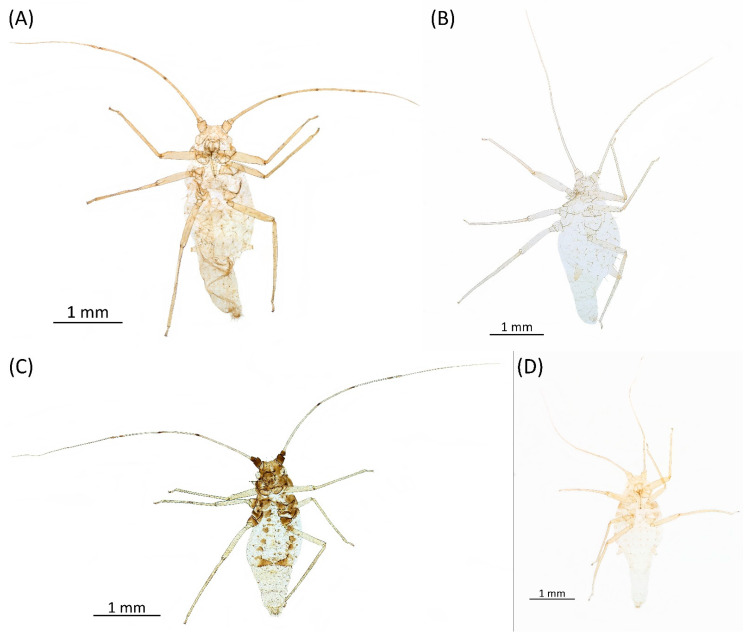
Oviparous females of the genus *Drepanaphis*: (**A**) *D. simpsoni*, (**B**) *D. spicata*, (**C**) *D. tissoti*, (**D**) *D. utahensis*.

**Figure 34:**
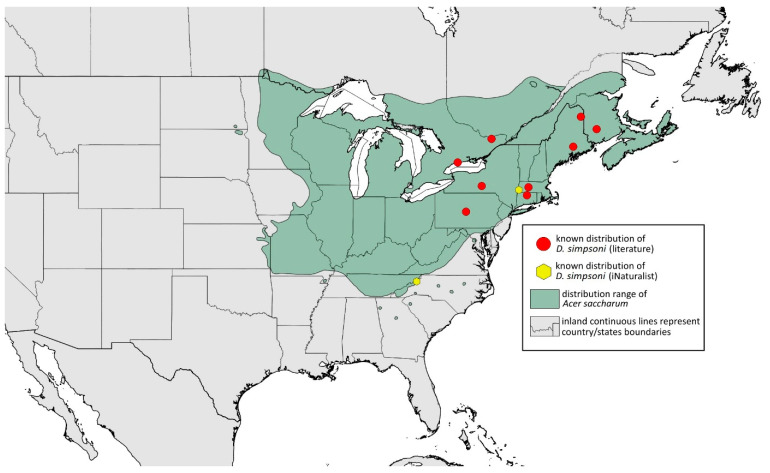
Known distribution of *Drepanaphis simpsoni* in North America, with distribution ranges of its host plants.

*Drepanaphis simpsoni* Smith, 1959: 52(6): 647 [26]
  = *Drepanaphis pallida* Richards, 1969: 101(1): 107 [28]
**Figure 1O, Figure 3O, Figure 4O, Figure 5O, Figure 11O, Figure 14J, Figure 24D, Figure 30C, Figure 33A and Figure 34; Table 2, Table 3 and Table 4**
  **Material examined:** Holotype. Drepanaphis simpsoni Smith, Holotype + Allotype C. F. S. Det: D. H. R. L. C. F. SMith//N. U. S. A. Pl. Acer saccharum, Loc. Presque Isle (Maine), Date 10.IX.1956, Leg. Simpson & H. R. L. 465, BM1984-340—two alate viv. fem., one alate viv. male. Paratype. Drepanaphis simpsoni Smith + kanzenis Smith, oviparae, Det. C. F. Smith//N. U. S. A. Pl. Acer saccharum, Loc. Presque Isle (Maine), Date 10.IX.1956, Leg. Simpson & H. R. L. 465, BM1984-340—three Viviparous ♀ ♀. Paratype. Drepanaphis simpsoni Smith, Acer saccharum//Presque Isle, Me., Oct. 9, 1958, Simpson//INHS, Insect Collection 1058916—one alate viv. fem.
  Additional material examined—Table S6.
**Alate viviparous female—re-description (n = 12)**
  **Colour. In life:** Head and thorax reddish brown, somewhat darker dorsally with short, fine, longitudinal, white wax stripes. Abdomen light yellow to white with DAT I reddish brown [27].
  **Pigmentation of mounted specimens:** Head, ANT I–II and pronotum brown (Figure 30C). Rest of thorax dark brown. ANT II–VI pale brown with darker apices on ANT III–V and dark area with primary rhinarium on ANT VI. Wings clear, pterostigma distinct, darkly pigmented, with small area inside without pigmentation (Figure 3O). DAT I dark; DAT II, III and IV pale brown on apices (Figure 1O). Cauda, subgenital and anal plate brown. Legs, abdomen and siphunculi pale. Fore femora darker on ends (Figure 4O).
  **Morphometric characters:** Head setae: two pairs of fronto-orbital setae, one pair of postero-dorsal setae, one pair of latero-dorsal setae on dorsal side, 0.01–0.03 mm long with pointed apices; two pairs of pointed frontal setae on ventral side, 0.04–0.05 mm long. ANT/BL 1.17–1.61; PT/BASE 4.62 -7.08. ANT III with 8–13 secondary rhinaria, BASE with 4 accessory rhinaria. URS with 4–6 accessory setae. Other ratios: ANT IV/ANT III 0.66–0.83; ANT V/ANT III 0.58–0.76; ANT VI/ANT III 0.98–1.33; URS/ANT III 0.1–0.12; URS/BASE 0.59–0.67; URS/SIPH 0.42–0.48; HT II/ANT III 0.13–0.17; HT II/BASE 0.77–1.0; TIBIA III/BL 0.42–0.56; SIPH/BL 0.07–0.11; SIPH/CAUDA 0.63–2.16. DAT I biggest, 0.17–0.22 mm long, DAT II 0.1–0.16 mm long, DAT III 0.08–0.16 mm long (Figure 11O), DAT IV smallest, 0.05–0.11 mm long. End of tubercles with setae 0.02 mm long. Dorsal setae 0.02–0.03 mm long with pointed apices. Siphunculi tubular (Figure 5O).
**Oviparous female—description (n = 3)**
  **Colour. In life:** Unknown.
  **Pigmentation of mounted specimens:** Head, thorax, siphunculi pale. ANT and legs pale brown, ANT II–VI with darker apices on ANT III–V and dark area with primary rhinarium on ANT VI. Cauda, subgenital and anal plate brown (Figure 33A).

**Morphometric characters:** Head setae: two pairs of fronto-orbital setae, one pair of postero-dorsal setae, one pair of latero-dorsal setae on dorsal side, 0.07–0.1 mm long with blunt apices; two pairs of pointed frontal setae on ventral side, 0.08–0.1 mm long. ANT/BL 1.04–1.17. Other antennal ratios: ANT VI/ANT III 0.93–1.82; PT/BASE 4.07–9.92; SIPH/BL 0.05–0.06; III FEMUR/BL 0.21–0.22; TIBIA III/BL 0.4–0.41; HT II/ANT VI 0.08–0.17; URS/ANT III 0.11–0.12; URS/BASE 0.57–0.75; URS/SIPH 0.53. ANT III without secondary rhinaria. URS with 6–9 accessory setae. Hind tibiae with 43 pseudosensoria more abundant in middle part of tibiae, closer to distal part. Dorsal setae 0.08–0.12 mm long, on ABD VIII slightly shorter. Siphunculi tubular.
**Alate male—re-description (n = 3)**
  **Colour. In life:** Unknown.
  **Pigmentation of mounted specimens:** Head, ANT I–II, thorax and siphunculi brown. ANT III–VI pale brown, ANT II–V with slightly darker apices on ends of segments and dark area with primary rhinarium on ANT VI. Wings clear, pterostigma palely pigmented, with large area inside without pigmentation. Cauda and anal plate brown. Abdomen pale with dark sclerotisation. Legs pale brown (Figure 24D).
  **Morphometric characters:** Head setae: two pairs of fronto-orbital setae, one pair of postero-dorsal setae, one pair of latero-dorsal setae on dorsal side, 0.01–0.03 mm long with pointed apices; two pairs of pointed frontal setae on ventral side, 0.03–0.04 mm long. ANT/BL 1.33–1.62. Other ratios: ANT VI/ANT III 0.99–1.14; PT/BASE 5.16–5.85; SIPH/BL 0.07–0.09; III FEMUR/BL 0.24–0.31; TIBIA III/BL 0.45–0.56; URS/ANT III 0.55–0.56; URS/SIPH 0.64–0.73. ANT III with 92–118 rhinaria, ANT IV with 62–82 rhinaria, ANT V with 30–38 rhinaria. URS with six accessory setae. DAT inconspicuous. Dorsal setae 0.02–0.03 mm long, with pointed apices. ABD II–VI with distinct spinal sclerites, each with 2–4 setae. Marginal sclerites with 1–3 setae. Siphunculi tubular. Genitalia with basal part of phallus rectangular, with broadly rounded apices (Figure 14J).
  **Remarks:** The analysis of type material of *D. pallida* confirmed its identity with *D. simpsoni*.
  **Host plant:** *Acer saccharum*.
  **Distribution:** Canada: New Brunswick (Fredericton); Ontario (New Castle, Ottawa). USA: Connecticut (Windsor); Maine (Orono, Presque Isle—locus typicus); Massachusetts (Amherst); New York (Ithaca); Pennsylvania (State College) (Figure 34) [26,27].

Additional distribution from iNaturalist (www.inaturalist.org, accessed on 12 June 2024): Massachusetts (Great Barrington); Tennessee (Roan Mountain).

#### 3.4.16. *Drepanaphis spicata* Smith, 1941

**Figure 35:**
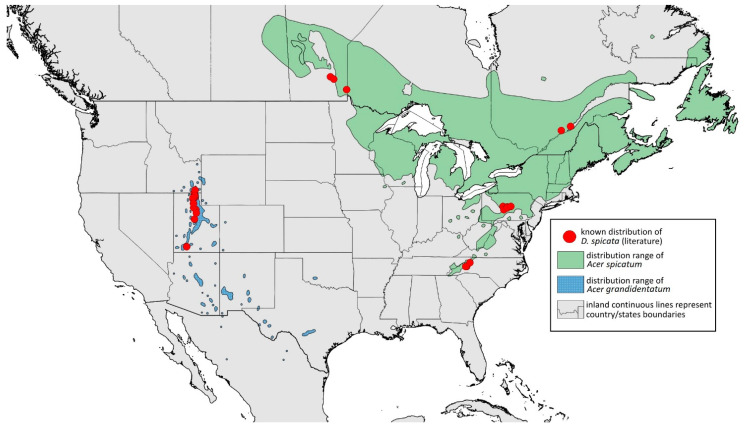
Known distribution of *Drepanaphis spicata* in North America, with distribution ranges of its host plants.

*Drepanaphis spicatum* Smith, 1941: 57(2): 228, 241 [23]
  = *Drepanaphis spicata* Palmer, 1952: 5:87 [51]
**Figure 1P, Figure 3P, Figure 4P, Figure 5P, Figure 11P, Figure 14K, Figure 24E, Figure 30D, Figure 33B and Figure 35; Table 2 and Table 4**
  **Material examined:** Holotype. Drepanaphis spicatum Smith Holotype Type No 55839. D.D.N.N.M.//N. C. 41-150, Acer spicatum, Mt. Mitchell, (Camp Alice), July 2, 1941, C. F. Smith, Remounted June 7,00—two alate viv. fem. (USNM). Paratype. Drepanaphis spicatum C. F. Smith//N. C. 41-188, Acer spicatum, On Park Way, Busic, N. C. 7-31-41, C. F. S.//INHS, Insect Collection 1058917—five alate viv. fem.
  Additional material examined—Table S6.
**Alate viviparous female—re-description (n = 19)**
  **Colour. In life:** Entire body powdery white except dark fuscous thoracic lobes, DAT III, siphunculi and brownish-yellow, U-shaped line more or less connecting DAT III to siphunculi. Fore femora dark [27].
  **Pigmentation of mounted specimens:** Head, ANT I–II and pronotum brown (Figure 30D). Rest of thorax dark brown. ANT II–VI pale brown with darker apices on ANT III–V and dark area with primary rhinarium on ANT VI. Wing veins clear, pterostigma distinct, very darkly pigmented, with small area inside without pigmentation (Figure 3P), radius veins dark brown. Abdomen pale with DAT III dark (Figure 1P) and dorsal sclerotisation dark. Cauda, subgenital and anal plate pale brown to brown. Fore femora brown, darker dorsally (Figure 4P). Middle femora pale brown, hind femora pale brown with dark stripes at margins.
  **Morphometric characters:** Head setae: two pairs of fronto-orbital setae 0.05–0.07 mm long, one pair of postero-dorsal setae, one pair of latero-dorsal setae on dorsal side, 0.03–0.05 mm long with pointed apices; one pair of pointed frontal setae on ventral side, 0.09–0.012 mm long. ANT/BL 1.77–2.35; PT/BASE 7.86–14.63. ANT III with 9–15 secondary rhinaria, BASE with 4 accessory rhinaria. URS with 6–8 accessory setae. Other ratios: ANT IV/ANT III 0.67–0.95; ANT V/ANT III 0.6–0.88; ANT VI/ANT III 1.19–2.06; URS/ANT III 0.08–0.1; URS/BASE 0.6–0.8; URS/SIPH 0.26–0.41; HT II/ANT III 0.08–0.11; HT II/BASE 0.63–0.85; TIBIA III/BL 0.54–0.73; SIPH/BL 0.1–0.18; SIPH/CAUDA 1.87–3.17. DAT III significant, 0.22–0.31 mm long (Figure 11P). Dorsal setae with pointed apices, 0.04–0.06 mm long, on small sclerites. Marginal sclerites with 3–6 setae. Siphunculi flask-shaped (Figure 5P).
**Oviparous female—description (n = 1)**
  **Colour. In life:** Unknown.
  **Pigmentation of mounted specimens:** Body in general pale brown, with slightly darker hind tibiae and ends of siphunculi (Figure 33B).
  **Morphometric characters:** Head setae: two pairs of fronto-orbital setae, one pair of postero-dorsal setae, one pair of latero-dorsal setae on dorsal side, 0.08–0.12 mm long with blunt apices; one pair of pointed frontal setae on ventral side, 0.11 mm long. ANT/BL 1.2. Other ratios: ANT VI/ANT III 1.19; PT/BASE 6.85; SIPH/BL 0.08–0.09; FEMUR III/BL 0.24–0.25; TIBIA III/BL 0.48–0.49; HT II/ANT VI 0.12; URS/ANT III 0.12; URS/BASE 0.77; URS/SIPH 0.43. URS with 10 accessory setae. Hind tibiae with 59–71 pseudosensoria distributed in central part of tibiae. Dorsal setae 0.1–0.13 mm long, on ABD VIII slightly shorter, to 0.09 mm long. Siphunculi flask-shaped.
**Alate male—re-description (n = 1)**
  **Colour. In life:** Unknown.
  **Pigmentation of mounted specimens:** Head, ANT I–II, thorax and siphunculi brown. ANT III–VI pale brown, ANT II–V with slightly darker apices on ends of segments and dark area with primary rhinarium on ANT VI. Wings clear, pterostigma distinct, darkly pigmented, with small area inside without pigmentation. Abdomen pale with dark sclerotisation. Cauda and anal plate brown. Legs pale, fore femora darker dorsally, hind femora with dark stripes on the apical parts (Figure 24E).
  **Morphometric characters:** Head setae: two pairs of fronto-orbital setae, one pair of postero-dorsal setae, one pair of latero-dorsal setae on dorsal side, 0.03–0.05 mm long with pointed apices; one pair of pointed frontal setae on ventral side, 0.07 mm long. ANT/BL 1.76–1.94. Other ratios: ANT VI/ANT III 1.58–1.24; PT/BASE 9.47–12.6; SIPH/BL 0.14; FEMUR III/BL 0.29; TIBIA III/BL 0.62; URS/ANT III 0.09; URS/SIPH 0.73. ANT III with 106–107 rhinaria, ANT IV with 46 rhinaria, ANT V with 23–25 rhinaria. URS with 12 accessory setae. Dorsal tubercles inconspicuous. Dorsal setae 0.04–0.06 mm long, with pointed apices. ABD I–VI with spinal sclerites and 2–4 setae. Marginal sclerites with 1–3 setae. Siphunculi flask-shaped. Genitalia with basal part of phallus short, robust and almost square-shaped (Figure 14K).
  **Remarks:** Smith and Knowlton [24] note in their article that individuals from Utah and Idaho are slightly different from other representatives of this species. They note the difference in abdominal dorsal tubercule III, usually longer, and with fewer and smaller dark areas around the hairs on the dorsal side of the abdomen.
  **Host plant:** *Acer spicatum*, specimens from Utah and Idaho known from *Acer grandidentatum*.
  **Distribution:** Canada: Manitoba (Caddy Lake, Camp Morton, Grand Beach); Quebec (Chute Panet, Saint-Nicolas). USA: Idaho (Franklin, Mink Creek); North Carolina (Buck Creek Gap, Grandfather Mountain, Mount Mitchell—locus typicus); Pennsylvania (Philipsburg, Pleasant Gap, Sprace Creek (Colerain Park), State College (Poe Paddy Laken Tussey Tower), Woodward); Utah (Big Cottonwood Canyon, Blacksmith Fork Canyon, City Creek Canyon, Logan Canyon, Mantua, Mount Nebo, Mount Sterling in Smithfield Canyon, Provo Canyon, Strawberry Creek, Weber Canyon) (Figure 35) [23,24,27].

#### 3.4.17. *Drepanaphis tissoti* Smith, 1944 stat. rev.

**Figure 36:**
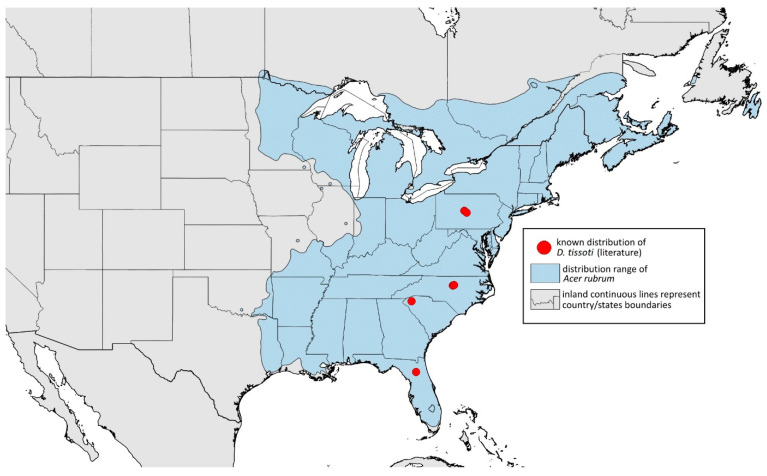
Known distribution of *Drepanaphis tissoti* in North America, with distribution ranges of its host plants.

*Drepanaphis tissoti* Smith, 1944: 27(3): 55 [25]
**Figure 1Q, Figure 2D, Figure 3Q, Figure 4Q, Figure 5Q, Figure 11Q, Figure 30E, Figure 33C and Figure 36; Table 2 and Table 3**
  **Material examined:** Holotype. Drepanaphis tissoti Smith, Det. CFSmith, Acer rubrum//Gainesville, Florida, Hatchetcreek, 5-5-1941, A. N. Tissot coll. F-2207-41. Paracotype. Drepanaphis tissoti Smith, Acer rubrum,//Gainesville, Florida, Hatchetcreek, 5-5-1941, A. N. Tissot coll. F-2207-41//Museum Paris MNHN 25154—one alate viv. fem. Paracotype. Drepanaphis tissoti Smith, Acer rubrum,//Gainesville, Florida, Hatchetcreek, 5-5-1941, A. N. Tissot coll. B. M.1967-340. F-2207-41//NHMUK 12821452—one alate viv. fem.
  Additional material examined—Table S6
**Alate viviparous female—re-description (n = 25)**
  **Colour. In life:** Black with pale legs, wings with noticeable dark spots at end of veins. Head with three, pronotum with two longitudinal white wax stripes. Mesonotum with two antero-lateral wax dots; two anteromedial dots usually connected to two postero-medial dots to make two longitudinal stripes. Metanotum with two wax dots laterally. Abdomen with many wax dots, most dense at posterior end [27].
  **Pigmentation of mounted specimens:** Head, thorax, ANT I–II dark brown (Figure 30E). ANT III–VI pale brown with darker apices on ANT III–V and dark area with primary rhinarium on ANT VI. Wings clear with dark pigmentation of ends of veins, radius veins dark brown. Pterostigma distinct, darkly pigmented, oval with small area inside without pigmentation (Figure 3Q). Abdomen pale with brown dorsal sclerotisation. Dorsal abdominal tubercles (Figure 1Q) and siphunculi dark brown. Cauda, subgenital and anal plate pale. Femora (Figure 4Q), tibiae and tarsi pale brown.
  **Morphometric characters:** Head setae: two pairs of fronto-orbital setae, one pair of postero-dorsal setae, one pair of latero-dorsal setae on dorsal side, 0.01–0.02 mm long with blunt apices. One pair of pointed frontal setae on ventral side, 0.05–0.06 mm long. ANT/BL 1.95–3.64; PT/BASE 7.2–16.8. ANT III with 8–11 secondary rhinaria, BASE with 5–11 accessory rhinaria (Figure 2D). URS with 6–9 accessory setae. Other ratios: ANT IV/ANT III 0.61–0.73; ANT V/ANT III 0.53–0.83; ANT VI/ANT III 1.18–2.72; URS/ANT III 0.11–0.13; URS/BASE 0.67–0.9; URS/SIPH 0.44–0.69; HT II/ANT III 0.1–0.13; HT II/BASE 0.65–0.85; TIBIA III/BL 0.57–0.88; SIPH/BL 0.09–0.18; SIPH/CAUDA 1.4–2.45. DAT I, II and IV inconspicuous. DAT III 0.15–0.19 mm long (Figure 11Q). Dorsal setae 0.01–0.02 mm long with blunt apices, on small sclerites. Siphunculi flask-shaped (Figure 5Q).
**Oviparous female—description (n = 1)**
  **Colour. In life:** Unknown.
  **Pigmentation of mounted specimens:** Head, ANT I–II, thorax, siphunculi dark brown. ANT and legs pale brown, ANT II–VI with darker apices on ANT III–V and dark area with primary rhinarium on ANT VI. Abdomen pale with dark sclerotisation. Cauda, subgenital and anal plate pale brown (Figure 33C).
  **Morphometric characters:** Head setae: two pairs of fronto-orbital setae, one pair of postero-dorsal setae, one pair of latero-dorsal setae on dorsal side, 0.06-.0.07 mm long with blunt apices; one pair of pointed frontal setae on ventral side, 0.09 mm long. ANT/BL 1.6. Other ratios: ANT VI/ANT III 1.78–1.82; PT/BASE 12.5–12.8; SIPH/BL 0.074–0.086; FEMUR III/BL 0.23; TIBIA III/BL 0.42; HT II/ANT VI 0.05–0.06; URS/ANT III 0.088; URS/BASE 0.67; URS/SIPH 0.44. URS with eight accessory setae. Hind tibiae with 30–31 pseudosensoria more abundant on the middle part of tibiae, closer to knee area. Dorsal setae 0.08–0.1 mm long, marginal sclerites with 1–4 setae each. Siphunculi flask-shaped.
  **Male:** Unknown.
  **Remarks:** Remaudière and Remaudière, based on personal communication from F.W. Quednau [29], establish the synonymisation of *D. tissoti* with *D. nigricans* without sufficient morphological study. However, the analysis and comparison of both species indicate differences (particularly the significant differences between oviparous females of these species). The main features that differentiate the alate viviparous females of these species are the number of secondary rhinaria on ANT III (in the case of *D. nigricans*, more than 11, in the case of *D. tissoti*, never more than 11) and the number of accessory rhinaria on BASE (*D. nigricans* always with 4, *D. tissoti* with 5–11). However, the features between oviparous females are also significant: hind tibiae with 53–62 pseudosensoria (*D. nigricans*) and 30–31 pseudosensoria (*D. tossoti*). Differences are also present in the respective ratios: ANT VI/ANT III, TIBIA III/BL, URS/BASE.
  **Host plants:** *Acer rubrum*, occasionally on *Acer saccharum*.
  **Distribution:** USA: Florida (Hatchet Creek near Gainesville—locus typicus); North Carolina (Chapel Hill, Hope Valley Forest); Pennsylvania (Black Moshannon State Park, State College); South Carolina (Easley) (Figure 36) [25,27].

#### 3.4.18. *Drepanaphis utahensis* Smith & Knowlton, 1943

**Figure 37:**
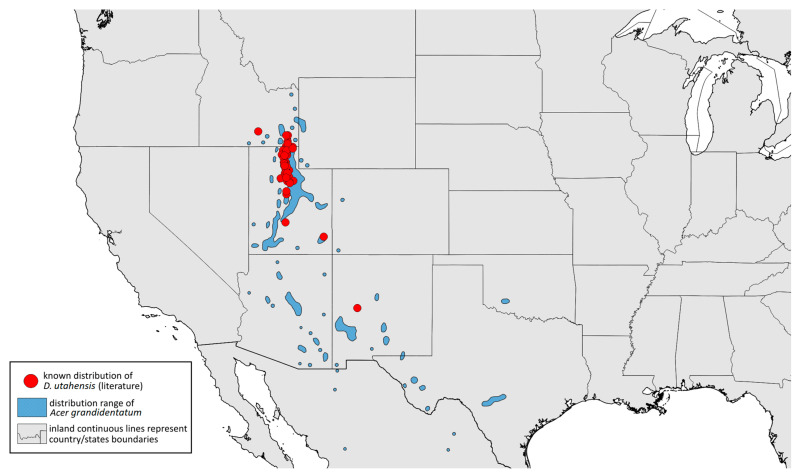
Known distribution of *Drepanaphis utahensis* in North America, with distribution ranges of its host plants.

*Drepanaphis utahensis* Smith & Knowlton, 1943: 59(2): 172, 174 [24]
**Figure 1R, Figure 3R, Figure 4R, Figure 5R, Figure 10C, Figure 11R, Figure 14L, Figure 25F, Figure 30F, Figure 33D and Figure 37; Table 2, Table 3 and Table 4**
  **Material examined:** Holotype. Drepanaphis utahensis K.-S.//Type Utah Aphids, Host maple, Drepanaphis utahensis K.-S. Brigham Canyon, Date 7-1-1937, C. F. Smith, C.K.S.—five alate viv. fem. (USNM). Paratype. Mt. Maple—Paratype., APHIDIDAE OF UTAH, Host Acer glabrum, Aphid Drepanaphis utahensis K-S, Locality Ogden Cany; Ut., May-20-1930, GEORGE F. KNOWLTON, Coll. very active. Heavy whitish pruinose, over head, thorax + abdomen. Ground color of abdomen green (not to dark)////INHS, Insect Collection 1058919—one alate viv. fem.
  Additional material examined—Table S6.
**Alate viviparous female—re-description (n = 26)**
  **Colour. In life:** Body yellow, head and pronotum edged in black, prescutum and thoracic lobes brown, DAT III dark; entire body frosted with white wax [27].
  **Pigmentation of mounted specimens:** Head, ANT I, pronotum and siphunculi brown (Figure 30F). Rest of thorax dark brown. ANT II–VI pale brown with slightly darker apices on ANT III–V and dark area with primary rhinarium on ANT VI. Wings clear with small area of dark brown pigmentation at end. Wings clear, pterostigma distinct, pigmented darker on ends, with small area inside without pigmentation (Figure 3R). Abdomen pale. DAT III dark (Figure 1R). Cauda, subgenital and anal plate pale brown. Legs pale brown. Fore femora darker on ends (Figure 4R).
  **Morphometric characters:** Head setae: two pairs of fronto-orbital setae, one pair of postero-dorsal setae, one pair of latero-dorsal setae on dorsal side, 0.03–0.04 mm long with pointed apices; two pairs of pointed frontal setae on ventral side 0.06–0.08 mm long. ANT/BL 0.94–2.05; PT/BASE 4.27–9.04. ANT III with 12–22 secondary rhinaria, BASE with 4 accessory rhinaria. URS with 6–10 accessory setae (Figure 10C). Other ratios: ANT IV/ANT III 0.57–0.88; ANT V/ANT III 0.5–0.87; ANT VI/ANT III 0.87–1.68; URS/ANT III 0.09–0.13; URS/BASE 0.49–0.73; URS/SIPH 0.33–0.46; HT II/ANT III 0.12–0.18; HT II/BASE 0.69–0.95; TIBIA III/BL 0.41–0.66; SIPH/BL 0.08–0.13; SIPH/CAUDA 1.51–2.36. DAT I, II and IV inconspicuous. DAT III distinct, 0.09–0.14 mm long (Figure 11R). Dorsal setae 0.03–0.04 mm long with pointed apices. Siphunculi tubular (Figure 5R).
**Oviparous female—description (n = 5)**
  **Colour. In life:** Unknown.
  **Pigmentation of mounted specimens:** Body generally pale brown with abdomen slightly lighter. Legs and dorsal sclerotisation slightly darker (Figure 33D).
  **Morphometric characters:** Head setae: two pairs of fronto-orbital setae, one pair of postero-dorsal setae 0.07–0.01 mm long, one pair of latero-dorsal setae on dorsal side 0.05–0.07 mm long with blunt apices; two pairs of pointed frontal setae on ventral side, 0.05–0.07 mm long. ANT/BL 0.88–1.04. Other ratios: ANT VI/ANT III 1.31–1.2; PT/BASE 5.92–7.77; SIPH/BL 0.05–0.066; FEMUR III/BL 0.19–0.21; TIBIA III/BL 0.36–0.42; HT II/ANT VI 0.1–0.12; URS/ANT III 0.12–0.17; URS/BASE 0.69–0.83; URS/SIPH 0.47–0.64. ANT III without secondary rhinaria. URS with 6–8 accessory setae. Hind tibiae with 13–52 pseudosensoria more abundant in middle part of tibiae. Dorsal setae 0.08–0.09 mm long. ABD I–II with large marginal sclerites. Siphunculi tubular.
**Alate male—re-description (n = 4)**
  **Colour. In life:** Unknown.
  **Pigmentation of mounted specimens:** Head, pronotum, ANT and siphunculi brown. Rest of thorax dark brown. Wings clear with closed, wide, brown pterostigma. Abdomen pale with dark sclerotisation. Cauda, anal plate and genitalia brown. Legs pale, hind femora with light brown smudge (Figure 24F).
  **Morphometric characters:** Head setae: two pairs of fronto-orbital setae, one pair of postero-dorsal setae, one pair of latero-dorsal setae on dorsal side, 0.02–0.03 mm long with pointed apices; two pairs of pointed frontal setae on ventral side, 0.03–0.06 mm long. ANT/BL 1.29–1.68. Other antennal ratios: ANT VI/ANT III 1.2–1.44; PT/BASE 6.14–7.3; SIPH/BL 0.09–0.11; FEMUR III/BL 0.26–0.3; TIBIA III/BL 0.51–0.64; URS/ANT III 0.11–0.12; URS/SIPH 0.47–0.53. ANT III with 78–83 rhinaria, ANT IV with 46–52 rhinaria, ANT V with 27–30 rhinaria. URS with eight accessory setae. DAT inconspicuous. Dorsal setae 0.02–0.03 mm long, with pointed apices. ABD II–V with spino-pleural sclerites with two setae each. Marginal sclerites with 3–6 setae. Siphunculi tubular. Genitalia with basal part of phallus short, robust, with broadly rounded apices (Figure 14L).
  **Remarks:** Archibald [61] reported an individual of this species from Nova Scotia on sugar maple, *A. saccharum* [27]. However, when analysing its range of occurrence, Archibald probably incorrectly identified this individual. The databases analysed also included records from Illinois and North Carolina. But the host plant was never *A. grandidentatum*, and we could not analyse these individuals, so we do not include their occurrence on the map. Smith and Knowlton [24] also mention that they had over 100 other slides from Utah and Idaho, but these specimens were brighter. However, they did not notice any other significant differences apart from colour. These individuals come from the following locations: Idaho (Boise (Minidoka National Forest), Franklin, Mink Creek); Utah (American Fork Canyon, Beaver Canyon, Big Cottonwood Canyon, Brigham Canyon, Eden, Emigration Canyon, Lakota, Mantua, Millville, Mt. Nebo, Oak Creek Canyon, Parley’s Canyon, Providence Canyon, Provo Canyon, Richmond, Sardine Canyon, Uinta).
  **Host plant:** *Acer grandidentatum*.
  **Distribution:** USA: Idaho (Bench^, Caribou-Targhee National Forest, Cub River Canyon); New Mexico (Cibola National Forest); Utah (Abajo Mountains, Blacksmith Fork Canyon, Bountiful (Skyline Drive), Brigham Canyon—locus typicus, City Creek Canyon, Cutler Dam, Daniels Canyon, Farmington Canyon, Honeyville, Huntsville, Liberty, Logan (Green Canyon), Mantua, Mount Nebo, Mount Timpanogos, Muller’s Park (Davis County), North Ogden, Ogden Canyon, Peterson, Pinecrest, Providence Canyon, Provo Canyon, Salt Lake City‴, Santaquin, Smithfield Canyon, Weber Canyon, Wellsville Canyon (also Mount Sterling), West Hodges Canyon) (Figure 37) [24,27]; International Barcode of Life project (iBOL) [^]; NMNH Extant Specimen Records (USNM, US) [‴].

### 3.5. Results of the Statistical Analysis

**Figure 38:**
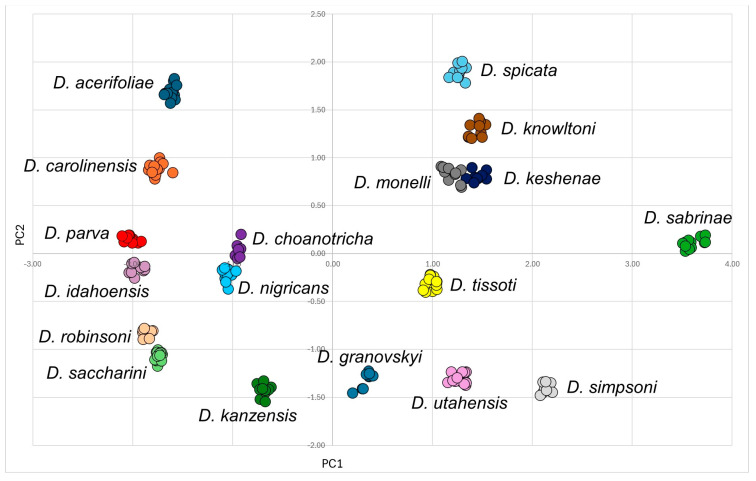
Plots of the first two components of a principal components analysis (PCA) for alate viviparous females of the genus *Drepanaphis*. Plots demonstrate the separation of individual species.

Based on the morphological and morphometric data, in the PCA results for the group consisting of the 213 alate viviparous females representing all species of the genus *Drepanaphis*, the first two axes of the PCA represent 70.8% of the total variance (the first three axes represent 80.9%). The first component (axis 1) is characterised mostly by the fore femora colour and the arrangement of conspicuous dorsal abdominal tubercles. The second component (axis 2) reflects the characteristics of the presence of stripes on the hind femora, dorsal sclerites and siphunculi colour (Figure 38; Table S3). In the case of all analysed morphs, qualitative features were decisive due to the fact that metric features often overlap, and there is no single clear feature that would allow for distinguishing individual species from each other.

**Figure 39:**
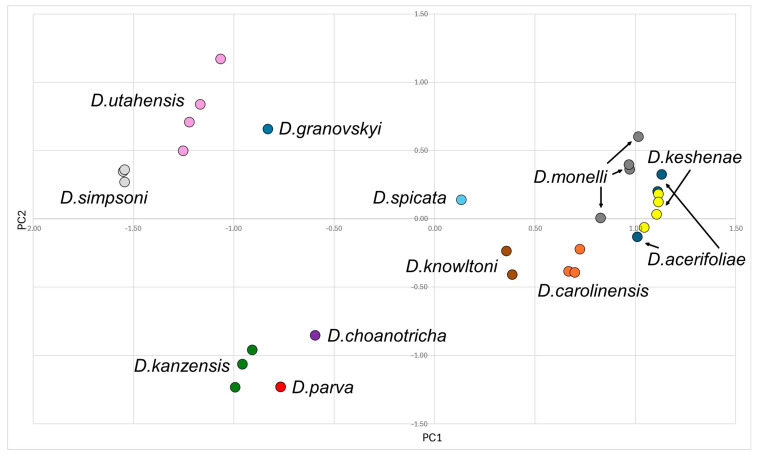
Plots of the first two components of a principal components analysis (PCA) for males of the genus *Drepanaphis* (without antennal segment ratio VI PT/BASE). Plots demonstrate the separation of individual species.

**Figure 40:**
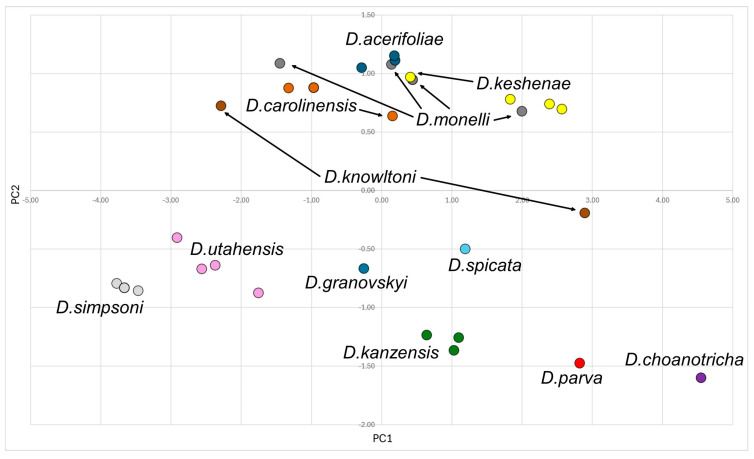
Plots of the first two components of a principal components analysis (PCA) for males of the genus *Drepanaphis* (with antennal segment ratio VI PT/BASE). Plots demonstrate the separation of individual species.

For 30 males representing 12 species of the genus *Drepanaphis*, the first two axes of the PCA represent 67.3% of the total variance (the first three axes represent 75.8%) in the variant where the antennal segment ratio VI PT/BASE variable is not used. When this characteristic is used, then the value increases to 86.9% (the first three axes represent 91.2%). The first component (axis 1) is characterised by the fore femora colour, the presence of the stripes on the hind femora and the antenna colour. The second component (axis 2) reflects mainly the length-to-height ratio in the middle part of the siphunculi, the number of frontal setae and the visible appearance of a third pair of tubercles on the abdomen segment (Figure 39; Table S4). In the analysis where the ratio of the length of the processus terminalis of the last antennal segment to that of its basal part was also used, this feature is dominant in the characterisation for axis 1 (Figure 40).

**Figure 41:**
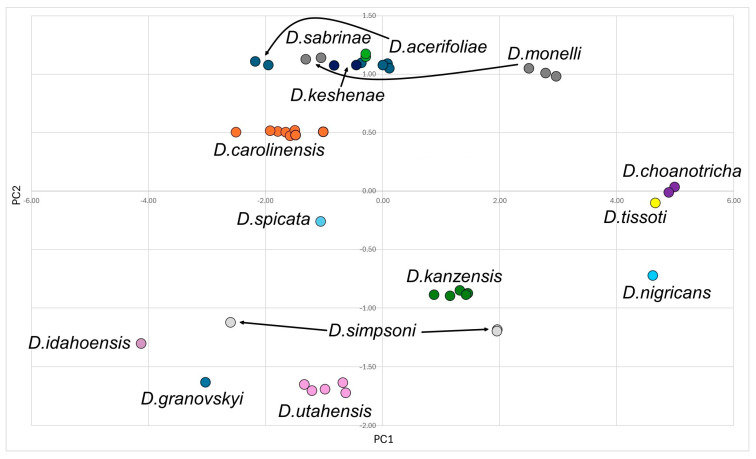
Plots of the first two components of a principal components analysis (PCA) for oviparous females of the genus *Drepanaphis*. Plots demonstrate the separation of individual species.

For 43 oviparous females representing 14 species of the genus *Drepanaphis*, the first two axes of the PCA represent 91.3% of the total variance (the first three axes represent 94.4%). The first component (axis 1) is characterised by the ratio of the length of the processus terminalis of the last antennal segment to that of its basal part. The second component (axis 2) reflects mainly the colour of antennae, fore tibiae, dorsal sclerites and siphunculi (Figure 41; Table S5).

The PCA results confirm the full species status of *D. tissoti* within the genus *Drepanaphis*. Moreover, according to previously proposed species groups by Smith and Dillery [27], most of these proposals are justified. In the genus under study, we can distinguish the “*acerifoliae*” group, characterised by four clearly visible pairs of dorsal abdominal tubercles in alate viviparous females. Originally, species such as *D. acerifoliae*, *D. carolinensis* and *D. sabrinae* were included here. However, *D. sabrinae* has a unique tubercle pattern, its siphunculi are light brown and unlike the other two species, which have four accessory rhinaria each, this species has five to six of them. This species is plotted for alate viviparous females at the far end of axis 1, but for oviparous females, it is shown close to *D. acerifoliae* and *D. carolinensis*. Species in the “*monelli*” group have only the third pair of tubercles visible and dark stripes on the hind femora. This group includes *D. keshenae*, *D. knowltoni*, *D. monelli* and *D. spicata*. Smith and Dillery [27] also proposed *D. kanzensis* here, although they also indicated some doubts about its position in this group. Based on the morphological analysis of this species, we propose not assigning it to any existing group, similar to the approach taken with *D. sabrinae*. The position of *D. kanzensis* outside the previously designated group is confirmed in the analyses of all generations—alate viviparous females and sexuales, oviparous females and males. In the case of sexual generation, species from the “*acerifoliae*” and “*monelli*” groups partially overlap, which is primarily due to the very similar metric ratio of the analysed characters and, especially in the case of males, the inability to measure some features in *D. acerifoliae* and *D. carolinensis* individuals (Figure 39, Figure 40 and Figure 41). For the correct analysis of the results, it is also important to mention that in oviparous females, the measurement values of the processus terminalis (ANT VI PT) for two individuals from *D. acerifoliae* and two from *D. monelli* were lower than those in the others, which resulted in some disturbance in the analyses and the separation of these individuals on the plot. This is the only reason for this situation.

The remaining groups coincide with the previous proposal. Therefore, we have a “*nigricans*” group, with *D. choanotricha*, *D. nigricans* and *D. tissoti*, mostly characterised by very long antennae; a “*parva*” group, where all species have a light-coloured front femora, i.e., *D. idahoensis*, *D. parva*, *D. robinsoni* sp. nov. and *D. saccharini*; and a fifth “*utahensis*” group, with *D. granovskyi*, *D. simpsoni* and *D. utahensis*, where all species have two pairs of frontal setae on the head.

## 4. Discussion

### 4.1. Morphological Groups within the Genus Drepanaphis

The most significant character of species belonging to the genus *Drepanaphis* is the presence of distinctive tubercles on dorsal abdominal tergites I–IV. The size and shape of dorsal abdominal tubercles have great importance in the systematics of this group of aphids. Smith and Dillery [27] pointed out the importance of this feature for distinguishing species belonging to five morpho-groups. However, in their original descriptions, they did not consider qualitative features such as the colour of the fore femora or the shape of the siphunculi. Additionally, when determining the groups, the authors paid great attention to the characteristics of the nymphs and the host plant associations, which may not provide sufficient information about the morphological similarities within the designated groups. They also did not consider the characteristics of the sexual generation, which were relatively poorly known. By analysing the morphological characters in the genus *Drepanaphis*, five distinct species groups can be distinguished. Species with four distinct pairs of dorsal abdominal tubercles, with the third pair being the largest, first femora dark around the edges and four accessory rhinaria are characteristic of the “*acerifoliae*” group, consisting of *D. acerifoliae* and *D. carolinensis*. The original division also included *D. sabrinae*, but due to different proportions of tubercles (the second and third pairs of equal length) and five accessory rhinaria, this species is not classified into any group. Additionally, *D. sabrinae* is the only species with an ultimate rostral segment 0.12–0.14 mm long. The second group of species morphologically similar to each other and distinguished by having only a third pair of tubercles is the “*monelli*” group (*D. keshenae*, *D. knowltoni*, *D. monelli* and *D. spicata*). Species in this group are very similar and are often incorrectly marked in museum collections; e.g., *D. knowltoni* was marked incorrectly as *D. monelli* and vice versa; *D. knowltoni* and *D. spicata* are also frequently confused species. In the case of this group, similarly to the “*acerifoliae*” group, another species, *D. kanzensis*, was originally included. Variability resulting from the colour of the fore femora and the lack of black stripes on the third pair of hind femora means that this species is also not classified into any group. A group of individuals characterised by relatively small body size, long antennae and a variable number of accessory rhinaria creates the “*nigricans*” group (*D. choanotricha*, *D. nigricans*, *D. tissoti*). Remaudière and Remaudière [29], after personal communication from Quednau, established the synonymisation of *D. tissoti* with *D. nigricans*, but our analysis indicates differences between them. A significant feature was the number of rhinaria, both secondary (*D. nigricans* with more than 11, *D. tissoti* never with more than 11) and accessory (*D. nigricans* always with 4, *D. tissoti* with 5–11). Species belonging to the “*parva*” group (*D. idahoensis*, *D. parva*, *D. robinsoni* sp. nov., *D. saccharini*) differ by pale fore femora. *Drepanaphis parva* and *D. robinsoni* sp. nov. have wing veins with slightly dusky areas. Apart from the similarity in the smudge on the wings, they differ in the presence of sclerotisation on the abdomen (Figure 21E,F) or the different shapes of the dorsal abdominal tubercles (Figure 11K,L). Representatives with two frontal setae in adults belong to the “*utahensis*” group (*D. granovskyi*, *D. simpsoni*, *D. utahensis*). *Drepanaphis granovskyi* and *D. utahensis* are light-coloured species with relatively small tubercles, which in the case of *D. simpsoni* are larger, and this species is the only one with the first pair of tubercles, the largest concerning the remaining pairs.

### 4.2. Morphometric Similarities in the Genus Drepanaphis

The phenomenon of morphological similarity in aphids is common and can be distinguished in many genera of hemipterans. An example is the genus *Aphis* L., where the species generally appear very similar due to convergence toward particular morphological types [62]. To distinguish some of them (*A. glycines*, *A. gossypii*, *A. rhamnicola*), it is necessary to use DNA barcoding because morphological comparative studies are insufficient [63]. While species in the *Drepanaphis* genus may initially appear different, primarily due to the varied arrangement of dorsal abdominal tubercles, and constitute a distinct group within the subfamily Drepanosiphinae [14], the morphometric features of most species overlap, making it impossible to establish clear differentiation ranges. The range of sizes of individuals in the genus is very wide, especially considering that the specimens analysed encompass forms from each season (from early spring to late autumn), which may vary substantially in body size. Additionally, the morphological plasticity of aphids [64] can affect a wide spectrum of ranges of dimensions. The morphometric feature that often carries significant taxonomic information is the length of the appropriate segments of the antennae. In some species of the genus *Drepanaphis*, the length of the processus terminalis of the last antennal segment may serve as a distinguishing feature, visible in well-preserved specimens. However, in the case of most mounted specimens, the last segment is often broken or destroyed, which can lead to erroneous conclusions regarding interspecies differences based on this feature [18]. Another qualitative feature is the number of rhinaria on antennal segment III, which should be relatively constant. In the case of some species, such as *D. choanotricha*, the number of rhinaria is small, which facilitates initial verification. However, the ranges of rhinaria numbers overlap for many species, rendering this feature less significant for identification purposes. The number of setae on the ultimate rostral segment is also a questionable diagnostic character.

Although SEM can provide excellent imaging of detailed morphological features in aphids [65], this method does not enable accurate counting of this character (Figure 10). Therefore, we included ranges of numerical intervals in the key rather than specific values in this case. Although quantitative features such as the number of accessory rhinaria on the base of antennal segment VI may differentiate species like *D. choanotricha*, *D. sabrinae* and *D. tissoti* (Figure 2), most morphometric features overlap. When verifying individuals in this genus, attention should primarily be given to qualitative features, which can aid in identifying living specimens. These features include dark-bordered wings in the case of *D. acerifoliae* and *D. keshenae* (Figure 8) and the colour of the fore femora. When analysing individuals using light microscopy, the qualitative features that hold the greatest diagnostic importance are the shape and size of the dorsal abdominal tubercles. Although in some species, their shape and size may be distorted by the incorrect positioning of individuals on the mounted specimen [18], this feature remains valuable, especially in morphologically very similar species.

### 4.3. Host Plant Ambiguity

After analysing specimens from various entomological collections, it was found that *Drepanaphis* species are associated with a much broader range of host plant species than previously believed. This applies in particular to species that were classified as monophagous. *Drepanaphis monelli* is the only species in this genus that feeds on *Aesculus glabra*; the remaining species feed on maples. Host plant associations were also used as a diagnostic feature in the key and differentiated the examined specimens. Among the analysed specimens of this species in the INHS collection, a series of specimens of *Drepanaphis monelli* were also found on *A. saccharum* and *A. saccharinum*. Following thorough analysis, the taxonomic identity of this species was confirmed, thereby rejecting the possibility of mislabelling the specimens. In this case, we suspect two phenomena: (I) accidental drift from a host plant to a neighbouring tree species or (II) a genuinely broader species range of the host plant. While we do not discount the possibility of drift, which is common in groups of small insects [66], it has been observed accidentally in other species within this genus, such as on ferns (*D. acerifoliae*) and mosses (*D. utahensis*). However, we are more inclined to claim that the number of host plant species is greater than expected, a conclusion supported by numerous other examples. *Drepanaphis kanzensis*, which mainly occurs on *A. saccharum* (confirmed by field research conducted in September 2022 in the USA), may also appear on *A. rubrum* or *A. saccharinum*. It is noteworthy that the sexual generation of this species primarily occurs on *A. saccharinum* rather than *A. saccharum*, contrary to common assumption. The question arises of whether the sexual generation in this genus is so difficult to collect because of the omission of host plants. An interesting example is the new species *D. robinsoni* sp. nov., initially misidentified as both *D. choanotricha*, associated with *A. saccharum*, and *D. parva*, which primarily feeds on *A. rubrum*. In this case, we face difficulties in determining the primary host plant of the new species. Although designating a single host plant facilitates initial verification and provides a reliable source of information about the species [67], the analysis of a representative number of specimens from the genus *Drepanaphis* reveals that they are more narrowly oligophagous than monophagous. This fact should be considered when using the identification key.

### 4.4. Distribution

Among all identified *Drepanaphis* species, *D. granovskyi*, *D. idahoensis* and *D. utahensis* stand out most prominently due to their distribution. They are the only ones associated exclusively with the western part of North America, and they all feed on *Acer grandidentatum*. However, morphological analysis indicates that *D. idahoensis* is less similar to other species. A similar distinction can be observed with *D. knowltoni*, which also inhabits the same geographical area and feeds on *A. grandidentatum* but exhibits distinct morphological characteristics. The case of *D. spicata* is also noteworthy. Representatives of this species occur in the northernmost part of the continent among all those analysed (with only *D. acerifoliae* also recorded above latitude 49°). Similar to *D. knowltoni*, *D. spicata* shows range fragmentation, although further field confirmation of these findings is necessary.

The ranges of the other species overlap, corresponding to the natural ranges of their host plants. There are strong indications that the original habitat of the first representatives of this genus was the eastern part of North America, and their evolution is closely linked to the spread of trees within the *Acer* genus [68].

## 5. Conclusions

The study of the genus *Drepanaphis* highlights its taxonomic complexity and the diverse host plant associations among its species. Morphological analyses reveal distinctiveness even among closely related species, underscoring the importance of comprehensive revision and accurate species identification tools. The geographical distribution patterns suggest evolutionary ties to the *Acer* genus, particularly in eastern North America.

## Figures and Tables

**Table 1:** Measurements (in mm) of alate viviparous females of *Drepanaphis* (part 1).

Character	*D. acerifoliae* n = 26	*D. carolinensis* n = 16	*D. choanotricha* n = 13	*D. granovskyi* n = 13	*D. idahoensis* n = 10	*D. kanzensis* n = 17	*D. keshenae* n = 11	*D. knowltoni* n = 24	*D. monelli* n = 11
BL	1.55–2.68	1.57–2.5	1.15–1.63	1.37–2.21	1.41–2.19	1.24–2.83	1.05–2.43	1.4–2.46	1.58–2.46
HW	0.28–0.4	0.28–0.39	0.2–0.27	0.21–0.31	0.22–0.3	0.2–0.38	0.25–0.35	0.25–0.35	0.28–0.35
ANT I-VI	3.19–5.29	3.02–4.11	2.04–4.1	1.77–2.94	3.17–4.68	3.26–4.7	3–4.75	3.54–4.8	3.34–4.76
ANT III	0.71–1.11	0.75–1.01	0.56–0.76	0.53–0.77	0.82–1.03	0.77–1.17	0.7–1.1	0.68–1.16	0.83–1.1
ANT IV	0.47–0.90	0.53–0.73	0.36–0.6	0.32–0.52	0.65–0.79	0.51–0.9	0.45–0.86	0.49–0.8	0.67–0.8
ANT V	0.5–0.87	0.53–0.7	0.41–0.57	0.32–0.53	0.62–0.92	0.51–0.87	0.47–0.85	0.49–0.85	0.66–0.8
ANT VI	1.06–1.76	0.71–1.36	1.35–2.11	0.44–1.03	0.83–1.88	0.97–2	1.04–1.8	1.3–2.1	0.97–2.06
BASE	0.11–0.16	0.13–0.18	0.11–0.14	0.08–0.14	0.12–0.17	0.12–0.19	0.1–0.15	0.11–0.17	0.14–0.16
PT	0.94–1.61	0.53–1.21	1.21–1.99	0.35–0.9	0.70–1.72	0.82–1.84	0.93–1.66	1.16–1.94	0.83–1.92
FEMUR I length	0.51–0.85	0.54–0.72	0.31–0.54	0.34–0.59	0.54–0.79	0.53–0.78	0.47–0.79	0.48–0.87	0.62–0.77
FEMUR I width	0.11–0.18	0.1–0.15	0.05–0.08	0.09–0.15	0.1–0.13	0.09–0.37	0.1–0.15	0.1–0.16	0.1–0.16
FEMUR III	0.42–0.76	0.43–0.65	0.31–0.48	0.31–0.54	0.49–0.7	0.45–0.80	0.37–0.66	0.41–0.8	0.52–0.7
TIBIA III	0.84–1.54	0.96–1.28	0.71–0.99	0.65–1.1	1.1–1.40	1–1.5	0.9–1.40	1–1.69	1.2–1.4
HT II	0.07–0.14	0.09–0.14	0.08–0.09	0.07–0.12	0.08–0.12	0.1–0.13	0.08–0.12	0.09–0.12	0.09–0.11
URS	0.09–0.11	0.09–0.10	0.09–0.1	0.07–0.09	0.08–0.10	0.08–0.1	0.08–0.09	0.07–0.1	0.1–0.12
SIPH	0.17–0.38	0.16–0.26	0.12–0.19	0.13–0.24	0.18–0.28	0.15–0.26	0.14–0.3	0.2–0.39	0.21–0.3
CAUDA	0.08–0.13	0.09–0.17	0.04–0.08	0.09–0.14	0.09–0.12	0.09–0.18	0.07–0.13	0.07–0.13	0.09–0.18

**Table 2:** Measurements (in mm) of oviparous females of *Drepanaphis*.

Character	*Drepanaphis*													
	*acerifoliae* n = 6	*carolinensis* n = 8	*choanotricha* n = 2	*granovskyi* n = 1	*idahoensis* n = 1	*kanzensis* n = 5	*keshenae* n = 2	*monelli* n = 5	*nigricans* n = 1	*sabrinae* n = 2	*simpsoni* n = 3	*spicata* n = 1	*tissoti* n = 1	*utahensis* n = 5
BL	2.74–3.08	1.82–2.81	1.77–2	2.15	2.12	2.44–3.32	2.22–2.3	1.99–2.7	2.14	2.42–2.44	2.78–2.85	2.87	2.43	2.64–3.24
HW	0.36–0.41	0.29–0.38	0.28–0.3	0.3	–	0.31–0.4	0.3–0.36	0.28–0.38	0.31	0.35–0.36	0.35–0.38	–	0.3	0.3–0.4
ANT I-VI	2.65–3.1	2.47–2.75	3.29–3.41	1.92–1.96	2.6–2.63	2.17–3.61	1.61–2.55	1.38–1.83	3.62–3.67	3.24–3.37	2.11–.3.3	3.44	3.87–3.92	2.56–3.24
ANT III	0.59–0.7	0.52–0.64	0.61–0.68	0.41–0.44	0.73–0.74	0.7–0.84	0.49–0.51	0.62–0.8	0.71–0.72	0.74–0.8	0.72–0.78	0.86–0.87	0.91	0.52–0.74
ANT IV	0.42–0.53	0.42–0.46	0.41–0.45	0.32–0.33	0.5–0.51	0.51–0.62	0.36–0.42	0.49–0.6	0.47–0.5	0.51–0.58	0.54–0.61	0.66–0.68	0.57–0.58	0.46–0.52
ANT V	0.46–0.58	0.43–0.49	0.47–0.49	0.35	0.58	0.56–0.61	0.41–0.45	0.57–0.64	0.6	0.55–0.59	0.51–0.67	0.66–0.7	0.6	0.5–0.55
ANT VI	0.88–1.2	0.86–1.05	1.59–1.72	0.66–0.75	0.64–0.67	1.18–1.52	0.89–1.06	1.17–1.7	1.65–1.74	1.2–1.31	0.71–1.31	1.02	1.62–1.66	0.9–1.16
BASE	0.12–0.14	0.13–0.15	0.12	0.11	0.12–0.13	0.13–0.15	0.11–0.12	0.13–0.16	0.12–0.13	0.14–0.16	0.12–0.14	0.13	0.12	0.12–0.14
PT	0.75–1.08	0.72–0.91	1.47–1.6	0.55–0.64	0.51–0.55	1.04–1.39	0.78–0.94	1.01–1.55	1.53–1.61	1.06–1.15	0.57–1.19	0.89	1.5–1.54	0.77–1.02
URS	0.1–0.11	0.09–0.1	0.09–0.1	0.08	0.1	0.08–0.09	0.08–0.12	0.11–0.12	0.1	0.12	0.08–0.09	0.1	0.08	0.09–0.1
FEMUR III	0.59–0.65	0.46–0.6	0.5–0.54	0.42–0.45	0.53–0.55	0.64–0.72	0.51–0.52	0.55–0.72	0.55–0.59	0.58–0.59	0.61–0.62	0.68–0.73	0.56–0.57	0.53–0.64
TIBIA III	1.08–1.26	0.91–1.12	0.9–0.95	0.84	1.08–1.09	1.21–1.38	1–.1.03	1.1–1.37	1.1	1.12–1.15	1.12–1.16	1.39–1.41	1.02	1.03–1.23
HT II	0.12–0.13	0.1–0.13	0.09–0.1	0.09	0.09–0.1	0.1–0.13	0.11–0.12	0.11–0.12	0.1	0.11–0.12	0.11–0.12	0.11–0.12	0.09–0.1	0.11–0.12
SIPH	0.2–0.25	0.13–0.19	0.21–0.24	0.17–0.2	0.17–0.19	0.17–0.19	0.14–0.17	0.2–0.27	0.18–0.19	0.2–0.24	0.15–0.18	0.23–0.27	0.18–0.21	0.14–0.21

**Table 3:** Measurements (in mm) of males of *Drepanaphis*.

Character	*Drepanaphis*											
	*acerifoliae* n = 3	*carolinensis* n = 3	*choanotricha* n = 1	*granovskyi* n = 1	*kanzensis* n = 3	*keshenae* n = 4	*knowltoni* n = 2	*monelli* n = 4	*parva* n = 1	*simpsoni* n = 3	*spicata* n = 1	*utahensis* n = 4
BL	2.09–2.21	1.75–2.26	1.44	1.82	1.85–2.44	1.67–2.11	2.01–2.1	1.96–2.16	2.48	1.62–2.19	2.82	1.7–2.47
HW	0.33–0.38	0.33–0.37	0.24	0.28	0.33–0.37	0.33–0.34	0.28–0.34	0.3–0.35	–	0.28–0.36	0.38	0.27–0.35
ANT I-VI	2.33–3.5	2.82–3.51	3.37–3.4	1.74–1.75	3.04–3.89	3.37–3.81	3.45–3.97	3.73–4.28	4.54–4.79	2.39–3.04	4.97–5.48	2.74–3.2
ANT III	0.72–0.94	0.73–1	0.69–0.71	0.71–0.73	0.86–1.05	0.77–1.05	0.92–1.0	0.96–1.02	1.12–1.1	0.66–0.87	1.27–1.29	0.75–0.82
ANT IV	0.59–0.64	0.5–0.67	0.43–0.44	0.41–0.42	0.53–0.71	0.53–0.64	0.6–0.64	0.66–0.78	0.79–0.81	0.47–0.63	0.98–1.0	0.46–0.62
ANT V	0.55–0.65	0.42–0.61	0.41–0.43	0.37–0.38	0.48–0.66	0.51–0.6	0.63–0.65	0.63–0.75	0.8–0.82	0.37–0.49	0.94	0.47–0.58
ANT VI	1.21	1.01–1.08	1.67–1.73	–	0.96–1.73	1.28–1.49	1.03–1.64	1.3–1.68	1.65–1.88	0.73–0.9	1.57–2.04	0.9–1.14
BASE	0.13–0.14	0.12–0.15	0.12	0.1–0.11	0.12–0.13	0.11–0.13	0.13–0.14	0.13–0.16	0.13	0.11–0.14	0.15	0.12–0.14
PT	1.08	0.88–0.95	1.55–1.61	–	0.83–1.61	1.17–1.36	0.89–1.51	1.15–1.55	1.52–1.75	0.62–0.76	1.42–1.89	0.78–1.0
URS	0.09	0.08–0.09	0.09	0.08	0.08	0.09–0.13	0.09–0.1	0.11–0.12	0.09	0.08–0.09	0.11	0.09
FEMUR III	0.55–0.63	0.46–0.63	0.41–0.44	–	0.56–0.7	0.49–0.65	0.58–0.65	0.63–0.67	0.73–0.74	0.43–0.59	0.82	0.48–0.64
TIBIA III	1.14–1.28	1.01–1.2	0.83–0.85	–	1.1–1.35	1.01–1.32	1.24–1.37	1.23–1.41	1.52	0.81–1.09	1.75	1.06–1.27
HT II	0.09–0.12	0.1–0.12	0.09	–	0.1–0.12	0.1–0.11	0.1–0.11	0.1–0.12	0.12	0.1–0.11	0.12	0.1–0.12
SIPH	0.23–0.27	0.16–0.23	0.17	0.16–0.17	0.19–0.23	0.17–0.28	0.25–0.26	0.25–0.28	0.19–0.23	0.15–0.16	0.39–0.4	0.17–0.23

## Data Availability

The original contributions presented in the study are included in the article and Supplementary Materials, further inquiries can be directed to the corresponding author/s.

## Supplementary Materials

The following supporting information can be downloaded at: https://www.mdpi.com/article/10.3390/insects15070553/s1, Table S1: Metric data and morphological characters for alate viviparous females of the genus *Drepanaphis*; Table S2: Metric data and morphological characters for males and oviparous females of the genus *Drepanaphis*; Table S3: Results of statistical analysis for alate viviparous females of the genus *Drepanaphis*; Table S4: Results of statistical analysis for males of the genus *Drepanaphis*; Table S5: Results of statistical analysis for oviparous females of the genus *Drepanaphis*; Table S6: Examined material of the *Drepanaphis* species.
